# Synthesis of Boronated Amidines by Addition of Amines to Nitrilium Derivative of Cobalt Bis(Dicarbollide) †

**DOI:** 10.3390/molecules26216544

**Published:** 2021-10-29

**Authors:** Ekaterina V. Bogdanova, Marina Yu. Stogniy, Kyrill Yu. Suponitsky, Igor B. Sivaev, Vladimir I. Bregadze

**Affiliations:** 1 A.N. Nesmeyanov Institute of Organoelement Compounds, Russian Academy of Sciences, 28 Vavilov Street, 119991 Moscow, Russia; bogdanovakatte@mail.ru (E.V.B.); kirshik@yahoo.com (K.Y.S.); sivaev@ineos.ac.ru (I.B.S.); bre@ineos.ac.ru (V.I.B.); 2 M.V. Lomonosov Institute of Fine Chemical Technology, MIREA—Russian Technological University, 86 Vernadsky Av., 119571 Moscow, Russia; 3 Basic Department of Chemistry of Innovative Materials and Technologies, G.V. Plekhanov Russian University of Economics, 36 Stremyannyi Line, 117997 Moscow, Russia

**Keywords:** cobalt bis(dicarbollide), nitrilium derivatives, amidines, nucleophilic addition reactions, synthesis, structure

## Abstract

A series of novel cobalt bis(dicarbollide) based amidines were synthesized by the nucleophilic addition of primary and secondary amines to highly activated B-N^+^≡C–R triple bond of the propionitrilium derivative [8-EtC≡N-3,3′-Co(1,2-C_2_B_9_H_10_)(1′,2′-C_2_B_9_H_11_)]. The reactions with primary amines result in the formation of mixtures of *E* and *Z* isomers of amidines, whereas the reactions with secondary amines lead selectively to the *E*-isomers. The crystal molecular structures of *E*-[8-EtC(NMe_2_)=HN-3,3′-Co(1,2-C_2_B_9_H_10_)(1′,2′-C_2_B_9_H_11_)], *E*-[8-EtC(NEt_2_)=HN-3,3′-Co(1,2- C_2_B_9_H_10_)(1′,2′-C_2_B_9_H_11_)] and *E*-[8-EtC(NC_5_H_10_)=HN-3,3′-Co(1,2-C_2_B_9_H_10_)(1′,2′-C_2_B_9_H_11_)] were determined by single crystal X-ray diffraction.

## 1. Introduction

Cobalt bis(dicarbollide) [3,3′-Co(1,2-C_2_B_9_H_11_)_2_]^−^ is the most available and the most stable of metallacarboranes [1,2,3,4], which attracts considerable attention of researchers due to possibility of its use in a variety of fields, from the development of new materials [5,6,7,8,9,10,11,12] to medicine [13,14,15,16,17,18,19,20,21,22,23,24,25,26,27,28]. Therefore, the development of new convenient methods for the functionalization of cobalt bis(dicarbollide) cobalt is an important task [3,4]. One of the convenient methods for the functionalization of the cobalt bis(dicarbollide) anion is the ring opening of its cyclic oxonium derivatives with various nucleophiles [29]. Another promising method is the addition of nucleophiles to the nitrilium derivatives of cobalt bis(dicarbollide). Nucleophilic addition reactions to the highly polarized –N^+^≡CR triple bonds in aryl- and alkylnitrilium salts [30] and nitrile complexes of transition metals [31,32,33,34,35] are widely used in organic synthesis and have large potential in the modification of polyhedral boron hydrides [36]. Thus, the nitrilium derivatives of the *closo*-decaborate anion react with water [37,38], alcohols [39], amines [40,41], hydrazines [42], hydrazones [42] and oximes [43,44], as well as with various carbanions [45,46] to form the corresponding addition products. Like organic nitrilium salts, they are able to participate in 1,3-dipolar cycloaddition reactions with azides and nitrones leading to the corresponding boronated tetrazoles [47] and 2,3-dihydro-1,2,4-oxadiazoles [48], respectively. Several examples of nucleophile addition reactions to the activated triple bond of the nitrilium derivatives of cobalt bis(dicarbollide) [8-RC≡N-3,3′-Co(1,2-C_2_B_9_H_10_)(1′,2′-C_2_B_9_H_11_)_2_] (R = Me, Ph) were also reported [49,50].

Recently, we initiated a systematic study of nucleophilic addition reactions to the activated triple bond of nitrilium derivatives of cobalt bis(dicarbollide) and described the synthesis of a series of imidates and thioimidates based thereof [51]. In this contribution, we report on the synthesis of boronated amidines by nucleophilic addition of primary and secondary amines to the propionitrilium derivative of cobalt bis(dicarbollide).

## 2. Results and Discussion

Amidines, that are the nitrogen analogues of carboxylic acids and esters, are well known and studied class of organic compounds [52,53,54,55,56]. Amidine derivatives are commonly used for the synthesis of many important heterocycles such as imidazoles, triazoles, thiazoles, oxadiazoles, pyrimidines, pyridines and triazines. The amidine moiety is a key pharmacophore in many biologically active compounds [57,58,59,60,61,62,63]. 1,8-Diazabicyclo[5.4.0]undec-7-ene (DBU) and some other amidines are used as organocatalysts in organic synthesis [64,65,66,67,68].

The direct addition of amines to nitriles is a straightforward and atom-economical approach to the synthesis of amidines. However, this approach is complicated by the need to activate organic nitriles by introducing electron-withdrawing substituents or by using harsh reaction conditions [52,53,54]. The use of various Lewis acids (AlCl_3_, ZnCl_2_, CaCl_2_, SmI_2_, TiCl_4_ or SnCl_4_) or organoaluminum compounds facilitates the addition of amines to organic nitriles to form amidines, but requires their stoichiometric equivalents along with nitrile and amine substrates. Of particular interest are the reactions of nitriles with amines in the presence of transition metal complexes, which can be used both in a stoichiometric ratio to study the reactions of coordinated nitriles [31,32], and in catalytic amounts in organic synthesis [69,70,71]. Synthesis of boronated amidines by the reaction of nucleophilic addition of amines to alkylnitrilium derivatives was first described for arachno-decaborate [72] and closo-decaborate [40,41,73,74,75,76] anions. Later, amidines on the base of nido-carborane [77,78,79] and the closo-dodecaborate anion [80] were synthesized. The reactions of [(8-RC≡N-3,3′-Co(1,2-C_2_B_9_H_10_)(1′,2′-C_2_B_9_H_11_] (R = Me, Ph) with n-butylamine and diethylamine resulting in the corresponding amidines were reported as well [49]. This prompted us to study in more detail the reactions of nitrilium derivatives of cobalt bis(dicarbollide) with various primary and secondary amines. The propionitrilium derivative of cobalt bis(dicarbollide) [51] was chosen for this study due to clear identification of ethyl group in NMR spectra and, first of all, in ^1^H NMR.

### 2.1. Nucleophilic Addition of Primary Amines

**Scheme 1:**
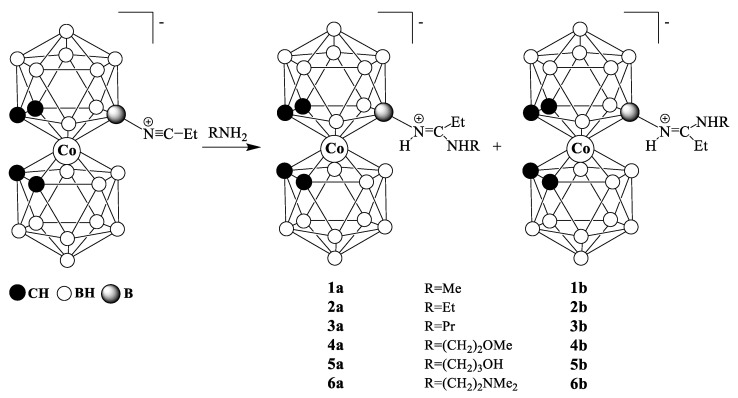
Nucleophilic addition reactions of primary amines to the propionitrilium derivative of cobalt bis(dicarbollide).

The nucleophilic addition reactions of primary amines (methylamine, ethylamine, propylamine, but also 3-amino-1-propanol, 2-methoxyethylamine, ethylenediamine and N,N-dimethylethylenediamine) to highly activated triple bond B-N^+^≡C-R of [8-EtC≡N-3,3′-Co(1,2-C_2_B_9_H_10_)(1′,2′-C_2_B_9_H_11_)] were studied. The reactions were carried out in acetonitrile solution in air at room temperature. The nucleophilic addition of amines occurs very fast and is completed in 5–10 min. In all cases, except for the reaction with ethylenediamine, the corresponding cobalt bis(dicarbollide) amidines **1**–**6** were obtained (Scheme 1). Purification of the products (if required) was carried out by column chromatography on silica.

Although 2-aminoethanol NH_2_CH_2_CH_2_OH, as ambidentate nucleophile, can be added to nitriles via nitrogen or oxygen atoms, it was reasonable to assume that the addition will occur through the more nucleophilic N-center. Indeed, we found that the reaction of [8-EtC≡N-3,3′-Co(1,2-C_2_B_9_H_10_)(1′,2′-C_2_B_9_H_11_)] with 2-aminoethanol leads exclusively to amidine **5**, rather than the corresponding amidate. It should be noted that the addition reactions of amino alcohols to the coordinated propionitrile in the platinum(IV) complex trans-[PtCl_4_(EtCN)_2_] proceed in a similar way [81].

In the case of ethylenediamine, the reaction with the propionitrilium derivative of cobalt bis(dicarbollide) lead to the destruction of the metallacarborane complex with the formation of a mixture of *nido*-carborane containing products. It should be noted that the reaction of the propionitrilium derivative of *nido*-carborane 10-EtC≡N-7,8-C_2_B_9_H_11_ with ethylenediamine led to the formation of ammonium derivative of *nido*-carborane 10-H_3_N-7,8-C_2_B_9_H_11_ instead of the expected amidine [77].

Amidines **1**–**6** were obtained as mixtures of *E* and *Z* isomers in nearly quantitative yields. The formation of *E* and *Z* isomers was earlier observed for cobalt bis(dicarbollide) based imidates and thioimidates [51]. For the imidates with short alkoxy substituent such as methoxy and ethoxy, it was possible to separate isomers by column chromatography on silica. However, in the case of the isopropoxy and butoxy substituents, as well as for the thioimidates, fast mutual isomerization of individual isomers in solution was observed.

**Figure 1:**
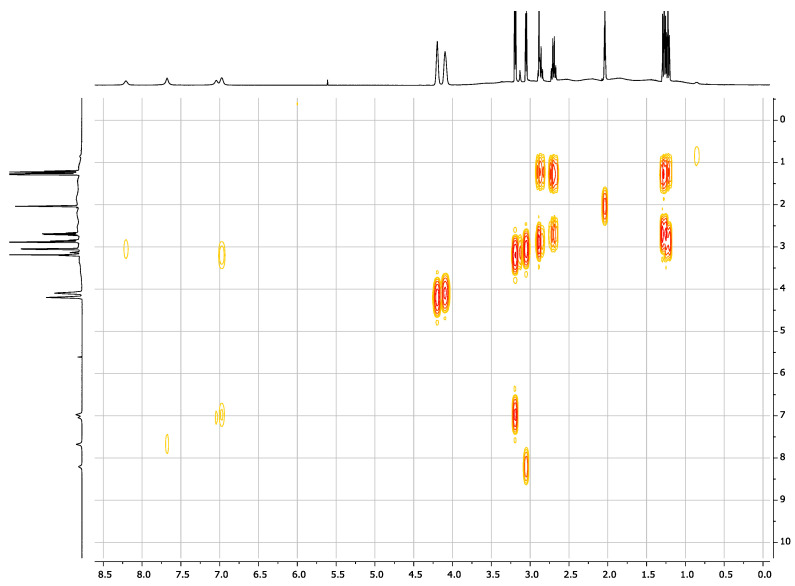
(HH)gCOSY NMR spectrum of amidines **1** in acetone-d_6_. The ratio of **1a**:**1b** is 1:1.3.

In the case of amidines, the formation of mixtures of *E* and *Z* isomers of **1**–**6** was detected using thin-layer chromatography, but all attempts to separate them by column chromatography on silica were failed. This can be explained by fast isomerization of pure isomers in solution with the formation of equilibrium mixtures of *E* and *Z* isomers, which is typical for organic amidines [82]. In all mixtures of isomers we observed an excess of *Z* isomers over *E* isomers with the *E*:*Z* ratio varying from 1:1.2 to 1:2.3 depending on the amine used (the measurements were performed by comparing the integrated intensities of signals of the same groups for different isomers in the ^1^H NMR spectra). A noticeable difference in the chemical shifts of the signals of the same groups for the *E* and *Z* isomers in the ^1^H and ^13^C NMR spectra made is possible to assign them to individual isomers using the (HH)gCOSY and (HC)HSQC NMR methods (Figure 1) as well as the spectral data of the amidines **7**–**10** obtained by the reactions with secondary amines (See below).

The ^1^H NMR spectra of *E* and *Z* isomers of amidines **1**–**6** are significantly different. The most sensitive to the isomer geometry are signals of atoms at the double bond. First of all, these are the signals of N*H* protons. In most of cases for the *E-*isomers the signal of the N*H*R hydrogen is in a lower field than the signal of the N*H*=C hydrogen, whereas for *Z-*isomers the position of these signals is opposite. The characteristic quartet form the C*H*_2_ group of the ethyl substituent in the ^1^H NMR spectra of the *E-*isomers is observed at 2.84–2.89 ppm, whereas for the *Z-*isomers this signal is high-field shifted and appears at 2.68–2.70 ppm. The signal from the NH–C*H*_2_– group generally appear in some higher field for the *E* isomers than for the *Z-*isomer (for example the signal of NH-C*H*_3_ appears at 3.06 ppm for **1a** and at 3.19 ppm for **1b**). 

The ^13^C NMR spectra of amidines **1**–**6** are much less sensitive to the isomer geometry and in many cases the signals of the same groups for different isomers coincide with each other, for example, the signals of the NH=*C* group, which appear in low field at ~167–169 ppm. Another example is the signals of the *C*H_2_ group of the ethyl substituent that are observed in higher field for the *E-*isomers (at ~24 ppm) in comparing with those for the *Z-*isomers (~25 ppm). 

In the ^11^B NMR spectra the most sensitive are the signals of the substituted boron atom. They appear at ~−12.5 for the *E-*isomers and at ~−10.5 ppm for the *Z-*isomers. The IR spectra of amidines **1**–**6** contain the characteristic absorption bands of the NH and N=C stretching as 3290–3380 and 1630–1640 cm^−1^, respectively.

Although the formation of mixtures of the *E* and *Z* isomers for organic amidines is well known [82], the presence of *E*- and *Z*-isomers in solutions of boronated amidines prepared by addition of primary amines to nitrilium derivatives of polyhedral boron hydrides was reported only for the *nido*-decaborane [72] and *nido*-carborane [77,79] based amidines. This could be an indication that for the other boronated amidines only one isomer is present in solution, or that the interconversion between the *E-*and *Z*-isomers is fast on the NMR time scale. However, in the solid state all these amidines have *Z*-configuration [40,49,75,76]. On the other hand, earlier, when studying the addition of primary amines to the coordinated nitrile ligands in platinum(II) complexes *cis*- and *trans*-[PtCl_2_(NCR)_2_], it was found that the reactions with the acetonitrile complexes (R = Me) produce exclusively the *Z*-amidines [83], while the reactions with the benzonitrile complexes (R = Ph) result in mixtures of the *E* and *Z* isomers [84]. Again, in a solid state the amidine ligands in these complexes have *Z*-configuration. Thus, the equilibrium between the *E* and *Z* isomers of amidines in solution largely depends on the substituents in the amidine fragment.

### 2.2. Nucleophilic Addition of Secondary Amines

**Scheme 2:**
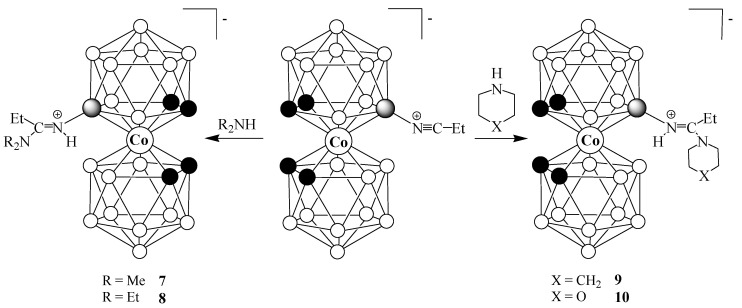
Nucleophilic addition of secondary amines to the propionitrilium derivative of cobalt bis(dicarbollide).

In contrast to the primary amines, the nucleophilic addition of secondary amines (dimethylamine, diethylamine, piperidine and morpholine) to the activated triple bond of [8-EtC≡N-3,3′-Co(1,2-C_2_B_9_H_10_)(1′,2′-C_2_B_9_H_11_)] results in the formation of exclusively *E* isomers of amidines **7**–**10** (Scheme 2). The formation of only *E* isomers was previously observed for the nucleophilic addition of secondary amines to the propionitrilium derivative of *nido*-carborane, whereas the reactions with primary amines resulted in mixtures of *E* and *Z* isomers [56].

**Figure 2:**
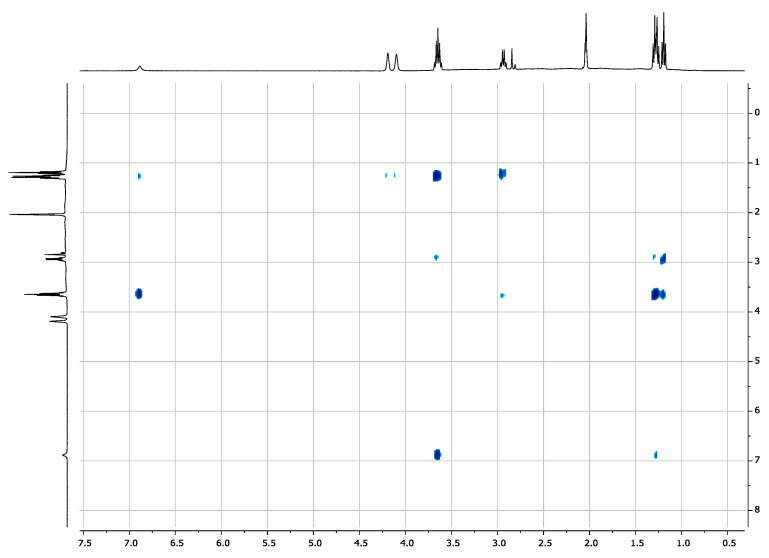
(HH)NOESY NMR spectrum of amidine **8** in acetone-d_6_ with diagonal peak suppression.

The assignment of the *E* isomers of amidines **7**–**10** was performed using the (HH)NOESY NMR method. The presence cross-peaks between the signals of the N*H* and OC*H*_2_ hydrogens clearly indicates the formation of the *E* isomers (Figure 2). 

The NMR spectral data of the *E* isomers of amidines **7**–**10** allowed us to verify the assignment of spectral signals made for the mixtures of *E* and *Z* isomers of amidines **1**–**6**. Thus, in the ^11^B NMR spectra of amidines **7**–**10** the singlet from substituted boron atom appears at ~13.0 ppm that corresponds to the chemical shift of this signal in the *E* isomers of compounds **1**–**6**. The comparison of other spectral data is also in good agreement. In the ^1^H NMR spectrum of amidine 7 the signals of two non-equivalent methyl groups of the dimethylamino fragment N(C*H*_3_)_2_ are observed as two singlets at 3.38 and 3.26 ppm, whereas for amidine 8 the signals of the diethylamino N(C*H*_2_C*H*_3_)_2_ fragment appear as a multiplet at 3.66 ppm and two triplets at 1.29 and 1.27 ppm. Similarly, in the ^13^C NMR spectrum of 7 there are two signals from the N(*C*H_3_)_2_ fragment at 39.9 and 37.8 ppm, whereas the signals from the N(*C*H_2_*C*H_3_)_2_ fragment of 8 are represented by two peaks in lower field at 45.0 and 41.9 ppm and two peaks in high field at 13.1 and 11.1 ppm. Such non-equivalence of alkyl groups at the nitrogen atom is due to restricted rotation around the C=N bond in the amidine fragment.

### 2.3. X-ray Diffraction Study

**Figure 3:**
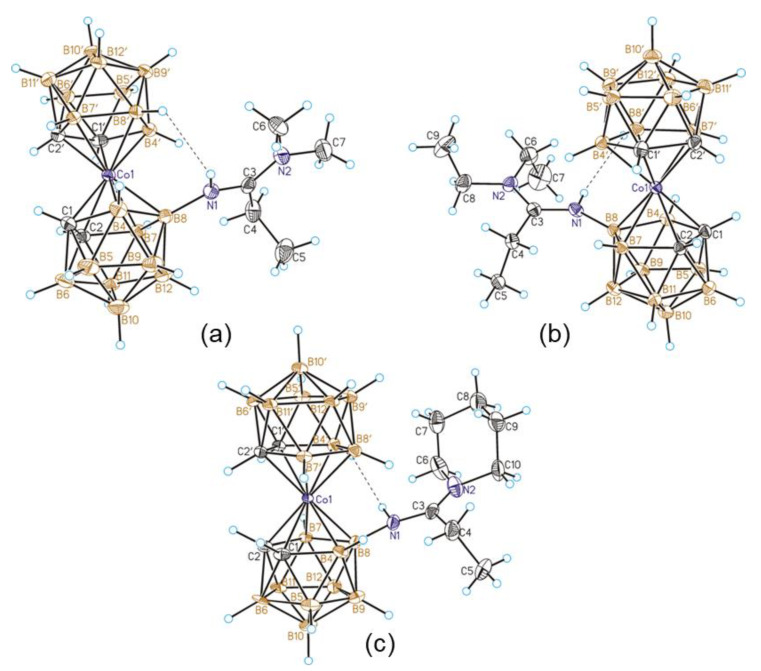
(**a**) The crystal molecular structures of *E*-[8-EtC(NMe_2_)=HN-3,3′-Co(1,2-C_2_B_9_H_10_)(1′,2′-C_2_B_9_H_11_)] (**7**); (**b**) *E*-[8-EtC(NEt_2_)=HN-3,3′-Co(1,2-C_2_B_9_H_10_)(1′,2′-C_2_B_9_H_11_)] (**8**) and (**c**) *E*-[8-EtC(NC_5_H_10_)=HN-3,3′-Co(1,2-C_2_B_9_H_10_)(1′,2′-C_2_B_9_H_11_)] (**9**).

Crystals of amidines 7–9, suitable for X-ray diffraction studies, were grown by slow evaporation of solutions in acetone-hexane or acetonitrile-hexane. The amidine fragments in all compounds have the *E* configuration with a nearly planar structure (Figure 3). The lengths of the B(8)–N(1), N(1)–C(3), C(3)–N(2) and C(3)–C(4) bonds are 1.518–1.522, 1.312–1.319, 1.327–1.330 and 1.491–1.498 Å, respectively, which are close to the values found earlier in [8-MeC(NEt_2_)=HN-3,3′-Co(1,2-C_2_B_9_H_10_)(1′,2′-C_2_B_9_H_11_)] [49]. The *E*-configuration was found also in amidines prepared by addition of secondary amines to nitrilium derivatives of other polyhedral boron hydrides ((Bu_4_N)[2- B_10_H_9_NH=C(N(CH_2_CH_2_)_2_O)Me] [41], (Bu_4_N)[2-B_10_H_9_NH=C(N(CH_2_)_5_)Me] [41], [2- B_10_H_9_NH=C(N(Me)(CH_2_)_3_NHMe_2_)Me] [73] and [6,9-B_10_H_12_(NH=C(NBu_2_)Me)_2_] [72]), as well as in the related amidine complexes of transition metals (*cis-* [PtCl_2_(N≡CPh)(NH=C(N(^t^Bu)CH_2_CH_2_NH^t^Bu))] [85], *cis-*[PtCl_2_(NH=(NMe_2_)Me)_2_] [86], *trans*-[PtCl_2_(NCMe)(NH=C(NMeBu*^t^*)Me)] [83], *cis*-[PtCl_2_(NH=C(NEt_2_)Me)_2_] [87], [Pd(NH=C(NEt_2_)Me)_4_][BF_4_]_2_ [87], [Cp*Ir(η^3^-CH_2_CHCHPh)(NH=C(NMe_2_)Me)](OTf) [88] and [(Quin)_2_Zn(NH=C(NC_4_H_8_)Me)] [89]).

The dicarbollide ligands in all structures are slightly (by 38.5–41.7°) rotated relative to each other, adopting the *cisoid* conformation. The substituents in compounds 7 and 8 are rotated in such a way that they form short NH···HB contacts of ~ 2.23–2.24 Å length with the B(8′)H group of the opposite unsubstituted dicarbollide ligand, whereas in the structure of 9 the short NH···HB contact of 1.76 Å is formed with the B(4′)H group of the opposite dicarbollide ligand. These distances are less than the sum of the van der Waals radii of two hydrogen atoms (2.4 Å), which is indicative of the weak N–H^δ+^···H^δ-^–B dihydrogen bonding that is commonly observed in compounds containing boron and nitrogen atoms [90,91,92,93,94,95,96,97,98,99,100,101]. It was shown that in the case of the imidate derivative of cobalt bis(dicarbollide) [8-EtC(O*^i^*Pr)=HN-3,3′-Co(1,2-C_2_B_9_H_10_)(1′,2′-C_2_B_9_H_11_)], the presence of even weaker NH···HB contact (2.31 Å) leads to additional stabilization of the *cisoid* conformation by ~2 kcal mol^−1^ [51]. Therefore, for the amidines, a more significant stabilization of the *cisoid* conformation can be expected, especially in the case of compound 9.

## 3. Conclusions

In this work, the nucleophilic addition reactions of primary and secondary amines to highly activated B–N^+^≡C–R triple bond of the propionitrilium derivative of cobalt bis(dicarbollide) anion [8-EtC≡N-3,3′-Co(1,2-C_2_B_9_H_10_)(1′,2′-C_2_B_9_H_11_)] were studied. As a result, a series of new metallacarborane-based amidines was synthesized. It was found out that the reactions with primary amines result in the formation of mixtures of the *E-* and *Z-*isomers, whereas the reactions with secondary amines leads selectively to the *E-* isomers. The crystal molecular structures of *E*-[8-EtC(NMe_2_)=HN-3,3′-Co(1,2- C_2_B_9_H_10_)(1′,2′-C_2_B_9_H_11_)], *E*-[8-EtC(NEt_2_)=HN-3,3′-Co(1,2-C_2_B_9_H_10_)(1′,2′-C_2_B_9_H_11_)] and *E*-[8- EtC(NC_5_H_10_)=HN-3,3′-Co(1,2-C_2_B_9_H_10_)(1′,2′-C_2_B_9_H_11_)] were determined by single crystal *X*-ray diffraction.

## 4. Experimental

### 4.1. Materials and Methods

The propionitrilium derivative of cobalt bis(dicarbollide) was prepared according to the literature procedure [51]. Methylamine, ethylamine and dimethylamine were generated from their concentrated aqueous solutions by the addition of K_2_CO_3_ at –5 °C. Propylamine, diethylamine, ethylenediamine, *N*,*N*-dimethylethylenediamine, 2-methoxyethylamine and 3-amino-1-propanol were purchased from Acros Organics and used without purification. Piperidine and morpholine were commercially analytical grade reagents and used without further treatment. Acetonitrile was dried using standard procedures [102]. All reactions were carried out in air. The reaction progress was monitored by thin-layer chromatography (Merck F254 silica gel on aluminum plates) and visualized using 0.5% PdCl_2_ in 1% HCl in aq. MeOH (1:10). Acros Organics silica gel (0.060–0.200 mm) was used for column chromatography. The NMR spectra at 400.1 MHz (^1^H), 128.4 MHz (^11^B) and 100.0 MHz (^13^C) were recorded in acetone-*d*_6_ with Varian Inova 400 spectrometer. The residual signal of the NMR solvent relative to tetramethylsilane was taken as the internal reference for ^1^H and ^13^C NMR spectra. ^11^B NMR spectra were referenced using BF_3_^.^Et_2_O as external standard. Infrared spectra were recorded on IR Prestige-21 (SHIMADZU) instrument. High resolution mass spectra (HRMS) were measured using Bruker micrOTOF II instrument with electrospray ionization (ESI). The measurements were performed in positive ion mode (interface capillary voltage—4500 V). A syringe injection was used for solutions in acetonitrile (flow rate 3 mL/min). Nitrogen was applied as a dry gas; interface temperature was set at 180 °C. 

### 4.2. General Procedure for Synthesis of Compounds **1**–**10**

To a solution of propionitrilium derivative of cobalt bis(dicarbollide) (0.20 g, 0.53 mmol) in acetonitrile (10 mL) amine (1–2 mL) was added and the solution was stirred for about 10 min at room temperature. The reaction mixture was evaporated to dryness in vacuum. The desired products were isolated by column chromatography on silica with CH_2_Cl_2_ or ethyl acetate as an eluent to give orange solids of **1**–**10**.

**[8-EtC(NHMe)=HN-3,3′-Co(1,2-C_2_B_9_H_10_)(1′,2′-C_2_B_9_H_11_)] (1a,1b)**

Yield 0.18 g (85%) (ratio **1a**:**1b** = 1:1.3)

**1a.**^1^H NMR (ppm): *δ* 8.20 (1H, s, N*H*CH_3_), 7.05 (1H, s, N*H*=C), 4.20 (2H, s, C*H*_carb_), 4.10 (2H, s, C*H*_carb_), 3.06 (3H, d, *J* = 4.9 Hz, NHC*H*_3_), 2.87 (2H, q, *J* = 7.5 Hz, C*H*_2_), 1.23 (3H, t, *J* = 7.5 Hz, C*H*_3_), 4.0–0.6 (17H, br s, B*H*). ^13^C NMR (ppm): *δ* 168.8 (NH=*C*), 52.7 (*C*H_carb_), 49.5 (*C*H_carb_), 27.5 (NH*C*H_3_), 24.0 (*C*H_2_), 10.9 (*C*H_3_).

**1b.**^1^H NMR (ppm): 7.68 (1H, s, N*H*=C), 6.98 (1H, s, N*H*CH_3_), 4.20 (2H, s, C*H*_carb_), 4.10 (2H, s, C*H*_carb_), 3.19 (3H, d, *J* = 5.1 Hz, NHC*H*_3_), 2.70 (2H, q, *J* = 7.6 Hz, C*H*_2_), 1.28 (3H, t, *J* = 7.6 Hz, C*H*_3_), 4.0–0.6 (17H, br s, B*H*). ^13^C NMR (ppm): *δ* 168.8 (NH=*C*), 52.4 (*C*H_carb_), 49.2 (*C*H_carb_), 29.6 (NH*C*H_3_), 24.9 (*C*H_2_), 9.8 (*C*H_3_).

^11^B NMR (ppm): *δ* 12.7 (s, **1a**), 10.6 (s, **1b**), 8.5 (d, *J* = 119 Hz), 2.9 (d, *J* = 144 Hz), −1.4 (d, *J* = 146 Hz), −4.3 (d, *J* = 146 Hz), -5.3 (d, *J* = 139 Hz), −6.7 (d, *J* = 144 Hz), −8.0 (d, *J* = 174 Hz), −16.2 (d, *J* = 156 Hz), −18.6 (d, *J* = 158 Hz), −21.6 (d, *J* = 156 Hz), −25.6 (d, *J* = 143 Hz). IR (film, cm^−1^): 3376 (ν_N-H_), 3294 (ν_N-H_), 3148 (ν_C-H_), 3040 (ν_C-H_), 2981 (ν_C-H_), 2945 (ν_C-H_), 2924 (ν_C-H_), 2570 (ν_B-H_), 2555 (ν_B-H_), 1640 (ν_N=C_), 1562, 1458, 1415, 1308, 1252. Supplementary Materials HRMS: *m/z* for C_8_H_31_B_18_CoN_2_: calcd 427.3947 [M+NH_4_]^+^, obsd 427.3930 [M+NH_4_]^+^.

**[8-EtC(NHEt)=HN-3,3′-Co(1,2-C_2_B_9_H_10_)(1′,2′-C_2_B_9_H_11_)] (2a,2b)**

Yield 0.20 g (88%) (ratio **2a**:**2b** = 1:1.8)

**2a**. ^1^H NMR (ppm): *δ* 8.08 (1H, s, N*H*CH_2_CH_3_), 7.07 (1H, s, N*H*=C), 4.19 (2H, s, C*H*_carb_), 4.08 (2H, s, C*H*_carb_), 3.46 (2H, m, NHC*H*_2_CH_3_), 2.84 (2H, q, *J* = 7.6 Hz, C*H*_2_), 1.29 (3H, t, *J* = 7.2 Hz, NHCH_2_C*H*_3_), 1.23 (3H, t, *J* = 7.6 Hz, C*H*_3_), 4.0–0.7 (17H, br s, B*H*). ^13^C NMR (ppm): *δ* 167.8 (NH=*C*), 52.3 (*C*H_carb_), 49.2 (*C*H_carb_), 36.3 (N*C*H_2_CH_3_), 24.0 (*C*H_2_), 12.7 (NCH_2_*C*H_3_), 11.4 (*C*H_3_). 

**2b.**^1^H NMR (ppm): 7.68 (1H, s, N*H*=C), 6.90 (1H, s, N*H*CH_2_CH_3_), 4.21 (2H, s, C*H*_carb_), 4.11 (2H, s, C*H*_carb_), 3.58 (2H, m, NHC*H*_2_CH_3_), 2.69 (2H, q, *J* = 7.6 Hz, C*H*_2_), 1.30 (3H, t, *J* = 7.2 Hz, NHCH_2_C*H*_3_), 1.28 (3H, t, *J* = 7.6 Hz, C*H*_3_), 4.0–0.7 (17H, br s, B*H*). ^13^C NMR (ppm): *δ* 167.6 (NH=*C*), 52.8 (*C*H_carb_), 49.4 (*C*H_carb_), 38.4 (N*C*H_2_CH_3_), 24.8 (*C*H_2_, t, *J* = 3.7 Hz), 14.4 (NCH_2_*C*H_3_), 10.4 (*C*H_3_).

^11^B NMR (ppm): *δ* 12.7 (s, **2a**), 10.5 (s, **2b**), 8.4 (d, *J* = 131 Hz), 3.1 (d, *J* = 136 Hz), −1.4 (d, *J* = 144 Hz), −4.3 (d, *J* = 119 Hz), −5.2 (d, *J* = 138 Hz), −6.7 (d, *J* = 185 Hz), −8.1 (d, *J* = 169 Hz), −16.1 (d, *J* = 152 Hz), −18.6 (d, *J* = 157 Hz), −21.6 (d, *J* = 160 Hz), −24.9 (d, *J* = 146 Hz). IR (film, cm^−1^): 3371 (ν_N-H_), 3314 (ν_N-H_), 3041 (ν_C-H_), 2982 (ν_C-H_), 2940 (ν_C-H_), 2894 (ν_C-H_), 2555 (br ν_B-H_), 1635 (ν_N=C_), 1559, 1452, 1417, 1388, 1344, 1245. Supplementary Materials HRMS: *m/z* for C_9_H_33_B_18_CoN_2_: calcd 446.3658 [M+Na]^+^, obsd 446.3647 [M+Na]^+^.

**[8-EtC(NHPr)=HN-3,3′-Co(1,2-C_2_B_9_H_10_)(1′,2′-C_2_B_9_H_11_)] (3a,3b)**

Yield 0.20 g (96%) (ratio **3a**:**3b** = 1:2.3)

**3a.**^1^H NMR (ppm): *δ* 8.10 (1H, s, N*H*CH_2_CH_2_CH_3_), 7.11 (1H, s, N*H*=C), 4.18 (2H, s, C*H*_carb_), 4.07 (2H, s, C*H*_carb_), 3.37 (2H, q, *J* = 7.0 Hz, NHC*H*_2_CH_2_CH_3_), 2.84 (2H, q, *J* = 7.6 Hz, C*H*_2_), 1.74 (2H, m, NHCH_2_C*H*_2_CH_3_), 1.23 (3H, t, *J* = 7.6 Hz, C*H*_3_), 0.96 (3H, t, *J* = 7.0 Hz, NHCH_2_CH_2_C*H*_3_), 3.9–0.6 (17H, br s, B*H*). ^13^C NMR (ppm): *δ* 169.3 (NH=*C*), 52.4 (*C*H_carb_), 49.3 (*C*H_carb_), 43.0 (NH*C*H_2_CH_2_CH_3_), 24.0 (*C*H_2_), 21.1(NHCH_2_*C*H_2_CH_3_), 11.5 (*C*H_3_), 10.7 (NHCH_2_CH_2_*C*H_3_).

**3b.** ^1^H NMR (ppm): 7.70 (1H, s, N*H*=C), 6.91 (1H, s, N*H*CH_2_CH_2_CH_3_), 4.21 (2H, s, C*H*_carb_), 4.11 (2H, s, C*H*_carb_), 3.50 (2H, q, *J* = 6.9 Hz, NHC*H*_2_CH_2_CH_3_), 2.69 (2H, q, *J* = 7.6 Hz, C*H*_2_), 1.71 (2H, m, NHCH_2_C*H*_2_CH_3_), 1.28 (3H, t, *J* = 7.6 Hz, C*H*_3_), 1.03 (3H, t, *J* = 7.0 Hz, NHCH_2_CH_2_C*H*_3_), 3.9–0.6 (17H, br s, B*H*). ^13^C NMR (ppm): *δ* 168.0 (NH=*C*), 52.8 (*C*H_carb_), 49.4 (*C*H_carb_), 45.0 (NH*C*H_2_CH_2_CH_3_), 24.9 (*C*H_2_), 22.8 (NHCH_2_*C*H_2_CH_3_), 10.5 (*C*H_3_), 10.3 (NHCH_2_CH_2_*C*H_3_).

^11^B NMR (ppm): *δ* 12.7 (s, **3a**), 10.5 (s, **3b**), 8.4 (d, *J* = 129 Hz), 3.1 (d, *J* = 134 Hz), −1.4 (d, *J* = 141 Hz), −4.4 (d, *J* = 127 Hz), −5.2 (d, *J* = 147 Hz), −6.7 (d, *J* = 186 Hz), −8.1 (d, *J* = 188 Hz), −16.1 (d, *J* = 157 Hz), −18.6 (d, *J* = 158 Hz), −21.6 (d, *J* = 164 Hz), −24.8 (d, *J* = 138 Hz). IR (film, cm^−1^): 3370 (ν_N-H_), 3324 (ν_N-H_), 3133 (ν_C-H_), 3042 (ν_C-H_), 2968 (ν_C-H_), 2937 (ν_C-H_), 2878 (ν_C-H_), 2588 (ν_B-H_), 2564 (ν_B-H_), 2530 (ν_B-H_), 1629 (ν_N=C_), 1555, 1464, 1418, 1387, 1344, 1249. Supplementary Materials HRMS: *m/z* for C_10_H_35_B_18_CoN_2_: calcd 455.4262 [M+NH_4_]^+^, obsd 455.4242 [M+NH_4_]^+^.

**[8-EtC(NHCH_2_CH_2_OMe)=HN-3,3′-Co(1,2-C_2_B_9_H_10_)(1′,2′-C_2_B_9_H_11_)] (4a,4b)**

Yield 0.22 g (93%) (ratio **4a**:**4b** = 1:2.0)

**4a.**^1^H NMR (ppm): *δ* 8.10 (1H, s, N*H*CH_2_CH_2_O*Me*), 7.86 (1H, s, N*H*=C), 4.16 (2H, s, C*H*_carb_), 4.09 (2H, s, C*H*_carb_), 3.61 (4H, m, NHC*H*_2_C*H*_2_O*Me*), 3.36 (3H, s, O*Me*), 2.88 (2H, q, *J* = 7.6 Hz, C*H*_2_), 1.23 (3H, t, *J* = 7.6 Hz, C*H*_3_), 4.0–0.9 (17H, br s, B*H*). ^13^C NMR (ppm): *δ* 167.9 (NH=*C*), 71.4 (NHCH_2_*C*H_2_O*Me*), 58.2 (NHCH_2_CH_2_O*Me*), 52.3 (*C*H_carb_), 49.3 (*C*H_carb_), 42.6 (NH*C*H_2_CH_2_O*Me*), 24.1 (*C*H_2_), 11.1 (*C*H_3_).

**4b.** ^1^H NMR (ppm): 7.68 (1H, s, N*H*=C), 7.28 (1H, s, N*H*CH_2_CH_2_O*Me*), 4.19 (2H, s, C*H*_carb_), 4.09 (2H, s, C*H*_carb_), 3.66 (2H, m, NHC*H*_2_CH_2_O*Me*), 3.62 (2H, m, NHCH_2_C*H*_2_O*Me*), 3.37 (3H, s, O*Me*), 2.70 (2H, q, *J* = 7.6 Hz, C*H*_2_), 1.27 (3H, t, *J* = 7.6 Hz, C*H*_3_), 4.0–0.9 (17H, br s, B*H*). ^13^C NMR (ppm): *δ* 167.9 (NH=*C*), 70.1 (NHCH_2_*C*H_2_O*Me*), 58.1 (NHCH_2_CH_2_O*Me*), 52.7 (*C*H_carb_), 49.5 (*C*H_carb_), 43.2 (NH*C*H_2_CH_2_O*Me*), 25.1 (*C*H_2_), 10.2 (*C*H_3_).

^11^B NMR (ppm): *δ* 12.7 (s, **4a**), 10.9 (s, **4b**), 8.3 (d, *J* = 123 Hz), 2.7 (d, *J* = 142 Hz), −1.3 (d, *J* = 138 Hz), −4.5 (d, *J* = 143 Hz), −5.4 (d, *J* = 144 Hz), −6.6 (d, *J* = 161 Hz), −8.0 (d, *J* = 176 Hz), −16.3 (d, *J* = 155 Hz), −18.8 (d, *J* = 158 Hz), −21.8 (d, *J* = 170 Hz), −25.1 (d, *J* = 177 Hz). IR (film, cm^−1^): 3354 (br ν_N-H_), 3041 (ν_C-H_), 2985 (ν_C-H_), 2934 (ν_C-H_), 2896 (ν_C-H_), 2834 (ν_C-H_), 2557 (br ν_B-H_), 1638 (ν_N=C_), 1558, 1456, 1417, 1388, 1249. Supplementary Materials HRMS: *m/z* for C_10_H_35_B_18_CoN_2_O: calcd 472.4169 [M+NH_4_]^+^, obsd 472.4160 [M+NH_4_]^+^.

**[8-EtC(NHCH_2_CH_2_CH_2_OH)=HN-3,3′-Co(1,2-C_2_B_9_H_10_)(1′,2′-C_2_B_9_H_11_)] (5a,5b)**

Yield 0.22 g (91%) (ratio **5a**:**5b** = 1:1.7)

**5a.**^1^H NMR (ppm): *δ* 8.15 (1H, s, N*H*CH_2_CH_2_CH_2_OH), 7.45 (1H, s, N*H*=C), 4.16 (2H, s, C*H*_carb_), 4.06 (2H, s, C*H*_carb_), 3.65 (2H, m, NHCH_2_CH_2_C*H*_2_OH), 3.53 (2H, q, *J* = 6.3 Hz, NHC*H*_2_CH_2_CH_2_OH), 2.87 (2H, q, *J* = 7.5 Hz, C*H*_2_), 1.85 (2H, m, *J* = 6.4 Hz, NHCH_2_C*H*_2_CH_2_OH), 1.23 (3H, t, *J* = 7.5 Hz, C*H*_3_), 3.9–0.7 (17H, br s, B*H*). ^13^C NMR (ppm): *δ* 168.5 (NH=*C*), 58.5 (NHCH_2_CH_2_*C*H_2_OH), 52.7 (*C*H_carb_), 49.2 (*C*H_carb_), 38.6 (NH*C*H_2_CH_2_CH_2_OH), 30.6 (NHCH_2_*C*H_2_CH_2_OH), 24.2 (*C*H_2_), 11.1 (*C*H_3_).

**5b.** ^1^H NMR (ppm): 7.66 (1H, s, N*H*=C), 7.05 (1H, s, N*H*CH_2_CH_2_CH_2_OH), 4.20 (2H, s, C*H*_carb_), 4.10 (2H, s, C*H*_carb_), 3.70 (2H, m, NHCH_2_CH_2_C*H*_2_OH), 3.64 (2H, m, NHC*H*_2_CH_2_CH_2_OH), 2.70 (2H, q, *J* = 7.6 Hz, C*H*_2_), 1.85 (2H, m, *J* = 6.4 Hz, NHCH_2_C*H*_2_CH_2_OH), 1.28 (3H, t, *J* = 7.5 Hz, C*H*_3_), 3.9–0.7 (17H, br s, B*H*). ^13^C NMR (ppm): *δ* 167.2 (NH=*C*), 58.3 (NHCH_2_CH_2_*C*H_2_OH), 52.7 (*C*H_carb_), 49.4 (*C*H_carb_), 40.8 (NH*C*H_2_CH_2_CH_2_OH), 32.1 (NHCH_2_*C*H_2_CH_2_OH), 25.0 (*C*H_2_), 10.4 (*C*H_3_).

^11^B NMR (ppm): *δ* 12.8 (s, **5a**), 10.6 (s, **5b**), 8.5 (d, *J* = 135 Hz), 3.1 (d, *J* = 139 Hz), −1.3 (d, *J* = 146 Hz), −4.3 (d, *J* = 149 Hz), −5.3 (d, *J* = 151 Hz), −6.7 (d, *J* = 185 Hz), −8.1 (d, *J* = 186 Hz), −16.2 (d, *J* = 160 Hz), −18.7 (d, *J* = 164 Hz), −21.7 (d, *J* = 162 Hz), −25.0 (d). IR (film, cm^−1^): 3370 (br ν_N-H_), 3325 (br ν_N-H_), 3130 (ν_C-H_), 3041 (ν_C-H_), 2944 (ν_C-H_), 2885 (ν_C-H_), 2564 (br ν_B-H_), 2536 (br ν_B-H_), 1635 (ν_N=C_), 1559, 1467, 1419, 1387, 1349, 1252. Supplementary Materials HRMS: *m/z* for C_10_H_35_B_18_CoN_2_O: calcd 472.4169 [M+NH_4_]^+^, obsd 472.4155 [M+NH_4_]^+^.

**[8-EtC(NHCH_2_CH_2_NMe_2_)=HN-3,3′-Co(1,2-C_2_B_9_H_10_)(1′,2′-C_2_B_9_H_11_)]** (**6a**,**6b**)

Yield 0.22 g (89%) (ratio **6a**:**6b** = 1:2.0)

**6a.**^1^H NMR (ppm): *δ* 10.16 (1H, s, N*H*=C), 7.94 (1H, s, N*H*CH_2_CH_2_NMe_2_), 4.14 (2H, s, C*H*_carb_), 4.08 (2H, s, C*H*_carb_), 3.47 (2H, m, NHC*H*_2_CH_2_NMe_2_), 2.89 (2H, q, *J* = 7.6 Hz, C*H*_2_), 2.58 (2H, m, NHCH_2_C*H*_2_NMe_2_), 2.31 (6H, s, N*Me*_2_), 1.22 (3H, t, *J* = 7.6 Hz, C*H*_3_), 4.0–0.8 (17H, br s, B*H*). ^13^C NMR (ppm): *δ* 167.1 (NH=*C*), 59.3 (NHCH_2_*C*H_2_NMe_2_), 52.2 (*C*H_carb_), 49.2 (*C*H_carb_), 45.3 (N*Me*_2_), 41.9 (NH*C*H_2_CH_2_NMe_2_), 24.2 (*C*H_2_), 11.0 (*C*H_3_).

**6b.** ^1^H NMR (ppm): *δ* 7.78 (1H, s, N*H*=C), 7.53 (1H, s, N*H*CH_2_CH_2_NMe_2_), 4.18 (2H, s, C*H*_carb_), 4.08 (2H, s, C*H*_carb_), 3.54 (2H, m, NHC*H*_2_CH_2_NMe_2_), 2.68 (2H, q, *J* = 7.6 Hz, C*H*_2_), 2.57 (2H, m, NHCH_2_C*H*_2_NMe_2_), 2.27 (6H, s, N*Me*_2_), 1.27 (3H, t, *J* = 7.6 Hz, C*H*_3_), 4.0–0.8 (17H, br s, B*H*). ^13^C NMR (ppm): *δ* 167.1 (NH=*C*), 56.2 (NHCH_2_*C*H_2_NMe_2_), 52.6 (*C*H_carb_), 49.4 (*C*H_carb_), 43.9 (N*Me*_2_), 40.4 (NH*C*H_2_CH_2_NMe_2_), 25.6 (*C*H_2_), 9.9 (*C*H_3_).

^11^B NMR (ppm): *δ* 12.7 (s, **6a**), 10.9 (s, **6b**), 8.3 (d, *J* = 123 Hz), 2.7 (d, *J* = 142 Hz), −1.3 (d, *J* = 142 Hz), −4.5 (d, *J* = 143 Hz), −5.4 (d, *J* = 144 Hz), −6.6 (d, *J* = 161 Hz), −8.0 (d, *J* = 176 Hz), −16.3 (d, *J* = 154 Hz), −18.8 (d, *J* = 160 Hz), −21.8 (d, *J* = 168 Hz), −25.1 (d, *J* = 164 Hz). IR (film, cm^−1^): 3309 (br ν_N-H_), 3041 (ν_C-H_), 2977 (ν_C-H_), 2948 (ν_C-H_), 2863 (ν_C-H_), 2828 (ν_C-H_), 2779 (ν_C-H_), 2568 (br ν_B-H_), 1629 (ν_N=C_), 1550, 1507, 1465, 1457, 1250. ESI HRMS: *m/z* for C_11_H_38_B_18_CoN_3_: calcd 489.4082 [M+Na]^+^, obsd 489.4085 [M+Na]^+^.

**[8-EtC(NMe_2_)=HN-3,3′-Co(1,2-C_2_B_9_H_10_)(1′,2′-C_2_B_9_H_11_)]** (**7**)

Yield 0.20 g (88%). ^1^H NMR (ppm): *δ* 6.93 (1H, s, N*H*), 4.17 (2H, s, C*H*_carb_), 4.08 (2H, s, C*H*_carb_), 3.38 (3H, s, NC*H*_3_), 3.26 (3H, s, NC*H*_3_), 2.95 (2H, q, *J* = 7.5 Hz, C*H*_2_), 1.18 (3H, t, *J* = 7.5 Hz, C*H*_3_), 3.9–0.7 (17H, br s, B*H*). ^13^C NMR (ppm): *δ* 168.5 (NH=*C*), 52.4 (*C*H_carb_), 49.2 (*C*H_carb_), 39.9 (N*C*H_3_), 37.8 (N*C*H_3_), 22.0 (*C*H_2_), 10.4 (*C*H_3_). ^11^B NMR (ppm): *δ* 13.1 (1B, s), 8.7 (1B, d, *J* = 142 Hz), 2.6 (1B, d, *J* = 144 Hz), −1.3 (1B, d, *J* = 146 Hz), −4.2 (2B, d, *J* = 169 Hz), −5.5 (2B, d, *J* = 135 Hz), −6.4 (2B, d, *J* = 132 Hz), −7.7 (2B, d, *J* = 163 Hz), −16.3 (2B, d, *J* = 162 Hz), −18.5 (2B, d, *J* = 163 Hz), −21.6 (1B, d, *J* = 167 Hz), −25.6 (1B, d, *J* = 169 Hz). IR (film, cm^−1^): 3385 (ν_N-H_), 3039 (ν_C-H_),, 2987 (ν_C-H_), 2944 (ν_C-H_), 2924 (ν_C-H_), 2854 (ν_C-H_), 2605 (ν_B-H_), 2576 (ν_B-H_), 2555 (ν_B-H_), 1615 (ν_N=C_), 1512, 1458, 1434, 1380, 1245. Supplementary Materials HRMS: *m/z* for C_9_H_33_B_18_CoN_2_: calcd 440.4135 [M+NH_4_]^+^, obsd 440.4121 [M+NH_4_]^+^.

**[8-EtC(NEt_2_)=HN-3,3′-Co(1,2-C_2_B_9_H_10_)(1′,2′-C_2_B_9_H_11_)]** (**8**)

Yield 0.22 g (91%). ^1^H NMR (ppm): *δ* 6.88 (1H, s, N*H*), 4.19 (2H, s, C*H*_carb_), 4.10 (2H, s, C*H*_carb_), 3.66 (4H, m, NC*H*_2_CH_3_), 2.94 (2H, q, *J* = 7.5 Hz, C*H*_2_), 1.29 (3H, t, *J* = 7.2 Hz, NCH_2_C*H*_3_), 1.27 (3H, t, *J* = 7.2 Hz, NCH_2_C*H*_3_), 1.19 (3H, t, *J* = 7.5 Hz, C*H*_3_), 3.9–0.7 (17H, br s, B*H*). ^13^C NMR (ppm): *δ* 167.7 (NH=*C*), 52.4 (*C*H_carb_), 49.2 (*C*H_carb_), 45.0 (N*C*H_2_CH_3_), 41.9 (N*C*H_2_CH_3_), 21.1 (*C*H_2_), 13.1 (NCH_2_*C*H_3_), 11.6 (*C*H_3_), 11.1 (NCH_2_*C*H_3_). ^11^B NMR (ppm): *δ* 13.2 (1B, s), 8.6 (1B, d, *J* = 141 Hz), 2.5 (1B, d, *J* = 144 Hz), −1.3 (1B, d, *J* = 144 Hz), −4.2 (2B, d, *J* = 152 Hz), −5.4 (2B, d, *J* = 139 Hz), −6.4 (2B, d, *J* = 142 Hz), −7.6 (2B, d, *J* = 141 Hz), −16.3 (2B, d, *J* = 160 Hz), −18.4 (2B, d, *J* = 170 Hz), −21.6 (1B, d, *J* = 163 Hz), −25.6 (1B, d, *J* = 175 Hz). IR (film, cm^−1^): 3389 (ν_N-H_), 3043 (ν_C-H_), 2975 (ν_C-H_), 2943 (ν_C-H_), 2608 (ν_B-H_), 2574 (ν_B-H_), 2545 (ν_B-H_), 1606 (ν_N=C_), 1507, 1496, 1450, 1383, 1358, 1238. Supplementary Materials HRMS: *m/z* for C_11_H_37_B_18_CoN_2_: calcd 470.4377 [M+NH_4_]^+^, obsd 470.4354 [M+NH_4_]^+^.

**[8-EtC(NC_5_H_10_)=HN-3,3′-Co(1,2-C_2_B_9_H_10_)(1′,2′-C_2_B_9_H_11_)]** (**9**)

Yield 0.23 g (94%). ^1^H NMR (ppm): *δ* 7.01 (1H, s, N*H*), 4.17 (2H, s, C*H*_carb_), 4.08 (2H, s, C*H*_carb_), 3.73 (4H, m, NC*H*_2_), 3.02 (2H, q, *J* = 7.5 Hz, C*H*_2_), 1.74 (6H, m, -C*H*_2_-), 1.16 (3H, t, *J* = 7.5 Hz, C*H*_3_), 4.0–0.7 (17H, br s, B*H*). ^13^C NMR (ppm): *δ* 173.1 (NH=*C*), 52.2 (*C*H_carb_), 49.4 (*C*H_carb_), 49.2 (N*C*H_2_), 46.3 (N*C*H_2_), 26.5 (*C*H_2_), 25.3 (*C*H_2_), 23.5 (*C*H_2_), 21.5 (*C*H_2_), 11.3 (*C*H_3_). ^11^B NMR (ppm): *δ* 13.2 (1B, s), 8.6 (1B, d, *J* = 141 Hz), 2.5 (1B, d, *J* = 144 Hz), −1.2 (1B, d, *J* = 145 Hz), −4.3 (2B, d, *J* = 153 Hz), −5.4 (2B, d, *J* = 117 Hz), −6.0 (2B, d, *J* = 119 Hz), −7.8 (2B, d, *J* = 152 Hz), −16.4 (2B, d, *J* = 154 Hz), −18.6 (2B, d, *J* = 156 Hz), −21.7 (1B, d, *J* = 166 Hz), −25.4 (1B, d, *J* = 151 Hz). IR (film, cm^−1^): 3381 (ν_N-H_), 3040 (ν_C-H_), 2976 (ν_C-H_), 2945 (ν_C-H_), 2862 (ν_C-H_), 2568 (br ν_B-H_), 1601 (ν_N=C_), 1506, 1485, 1457, 1445, 1382, 1361, 1248. Supplementary Materials HRMS: *m/z* for C_12_H_37_B_18_CoN_2_: calcd 502.3715 [M+K]^+^, obsd 502.3714 [M+K]^+^.

**[8-EtC(NC_4_H_8_O)=HN-3,3′-Co(1,2-C_2_B_9_H_10_)(1′,2′-C_2_B_9_H_11_)]** (**10**)

Yield 0.23 g (93%). ^1^H NMR (ppm): *δ* 7.17 (1H, s, N*H*), 4.17 (2H, s, C*H*_carb_), 4.08 (2H, s, C*H*_carb_), 3.79 (8H, m, NC*H*_2_ + OC*H*_2_), 3.06 (2H, q, *J* = 7.5 Hz, C*H*_2_), 1.17 (3H, t, *J* = 7.5 Hz, C*H*_3_), 3.9–0.6 (17H, br s, B*H*). ^13^C NMR (ppm): *δ* 170.4 (NH=*C*), 69.3 (O*C*H_2_), 68.4 (O*C*H_2_), 55.1 (*C*H_carb_), 52.1 (*C*H_carb_), 51.2 (N*C*H_2_), 48.4 (N*C*H_2_), 24.2 (*C*H_2_), 13.8 (*C*H_3_). ^11^B NMR (ppm): *δ* 13.0 (1B, s), 8.6 (1B, d, *J* = 131 Hz), 2.6 (1B, d, *J* = 140 Hz), −1.0 (1B, d, *J* = 143 Hz), −4.3 (2B, d, *J* = 137 Hz), −5.4 (2B, d, *J* = 123 Hz), −6.0 (2B, d, *J* = 121 Hz), −7.8 (2B, d, *J* = 153 Hz), −16.4 (2B, d, *J* = 156 Hz), −18.6 (2B, d, *J* = 158 Hz), −21.7 (1B, d, *J* = 147 Hz), −25.3 (1B, d, *J* = 136 Hz). IR (film, cm^−1^): 3377 (ν_N-H_), 3338 (ν_N-H_), 3041 (ν_C-H_), 2977 (ν_C-H_), 2929 (ν_C-H_), 2860 (ν_C-H_), 2588 (ν_B-H_), 2563 (ν_B-H_), 2534 (ν_B-H_), 1604 (ν_N=C_), 1588, 1506, 1456, 1382, 1363, 1249. Supplementary Materials HRMS: *m/z* for C_11_H_35_B_18_CoN_2_O: calcd 488.3765 [M+Na]^+^, obsd 488.3748 [M+Na]^+^.

### 4.3. Single Crystal X-ray Diffraction Study

X-ray experiments for compounds **7**, **8** and **9** were carried out using SMART APEX2 CCD diffractometer (λ(Mo-Kα)=0.71073 Å, graphite monochromator, *ω*-scans) at 120 K. Collected data were processed by the SAINT and SADABS programs incorporated into the APEX2 program package [103]. The structures were solved by the direct methods and refined by the full-matrix least-squares procedure against *F*^2^ in anisotropic approximation. The refinement was carried out with the SHELXTL program [104]. Compound **9** crystallizes in the form of dihydrate. All water molecules are significantly disordered and were eliminated from the refinement using common SQUIZZE option. The CCDC numbers (2114706, 2114707 and 2114708, for **7**, **8** and **9**, respectively) contain the supplementary crystallographic data for this paper. These data can be obtained free of charge via www.ccdc.cam.ac.uk/data_request/cif (accessed on 9 October 2021).

***Crystallographic data for 7****:* C_9_H_33_B_18_CoN_2_ are orthorhombic, space group *Pna*2_1_: *a* = 27.1787(4) Å, *b* = 7.08410(10) Å, *c* = 11.5151(2) Å, *V* = 2217.08(6) Å^3^, *Z* = 4, *M* = 422.88, *d*_cryst_ = 1.267 g·cm^−3^. *wR*2 = 0.0619 calculated on *F*^2^*_hkl_* for all 5112 independent reflections with 2*θ* < 56.0°, (*GOF* = 1.078, *R* = 0.0277 calculated on *F_hkl_* for 4862 reflections with *I* > 2*σ*(*I*)).

***Crystallographic data for 8****:* C_11_H_37_B_18_CoN_2_ are orthorhombic, space group *Pca*2_1_: *a* = 13.3717(4) Å, *b* = 16.2810(5) Å, *c* = 11.2565(3) Å, *V* = 2450.59(12) Å^3^, *Z* = 4, *M* = 450.93, *d*_cryst_ = 1.222 g·cm^−3^. *wR*2 = 0.0734 calculated on *F*^2^*_hkl_* for all 5343 independent reflections with 2*θ* < 54.2°, (*GOF* = 1.025, *R* = 0.0326 calculated on *F_hkl_* for 4406 reflections with *I* > 2*σ*(*I*)).

***Crystallographic data for 9****:* C_12_H_37_B_18_CoN_2_·2H_2_O are tetragonal, space group *I*4_1_/*a*: *a* = *b* = 39.0901(11) Å, *c* = 6.9515(3) Å, *V* = 10,622.1(8) Å^3^, *Z* = 16, *M* = 498.98, *d*_cryst_ = 1.248 g·cm^−3^. *wR*2 = 0.1626 calculated on *F*^2^*_hkl_* for all 5836 independent reflections with 2*θ* < 54.3°, (*GOF* = 1.028, *R* = 0.0684 calculated on *F_hkl_* for 3384 reflections with *I* > 2*σ*(*I*)).

## Supplementary Materials

The following are available online. Copies of 1H, 13C-NMR, 11B NMR, (HH)gCOSY NMR, NOESY NMR and main crystallographic data for compounds 7, 8 and 9.
