# Supplementary material for: Synthesis of Boronated Amidines by Addition of Amines to Nitrilium Derivative of Cobalt Bis(Dicarbollide)
Source: Molecules. 2021 Oct 29;26(21):6544. doi: 10.3390/molecules26216544 (PMC8588172; doi:10.3390/molecules26216544)
Supplement: Supplementary file 1 [file molecules-26-06544-s001.zip › molecules-1435092-supplementary.pdf]

Supplementary Materials

to the article

Synthesis of Boronated Amidines by Addition of Amines to Nitrilium Derivative of Cobalt Bis(Dicarbollide)

Ekaterina V. Bogdanova [1,2], Marina Yu. Stogniy[1,2], Kyrill Yu. Suponitsky[1,3], Igor B. Sivaev[1], Vladimir I. Bregadze[1]

[1] A.N. Nesmeyanov Institute of Organoelement Compounds, Russian Academy of Sciences, 28 Vavilov Str., 119991, Moscow, Russia

[2] M.V. Lomonosov Institute of Fine Chemical Technology, MIREA – Russian Technological University, 86 Vernadsky Av., 119571, Moscow, Russia

[3] Basic Department of Chemistry of Innovative Materials and Technologies, G.V. Plekhanov Russian University of Economics, 36 Stremyannyi Line, 117997 Moscow, Russia

The NMR spectra at 400.1 MHz (^{1}H), 128.4 MHz (^{11}B) and 100.0 MHz (^{13}C) were recorded with a Varian Inova-400 spectrometers. All spectra were processed in MestRenova version 6.0.2-5475. When processing ^{11}B NMR {^{1}H} spectra, the baseline alignment was applied to improve the quality of integration.

Spectral data for [(8-EtC(NHMe)=HN-3,3'-Co(1,2-$C_2B_9H_{10}$)(1',2'-$C_2B_9H_{11}$] (1a, 1b)

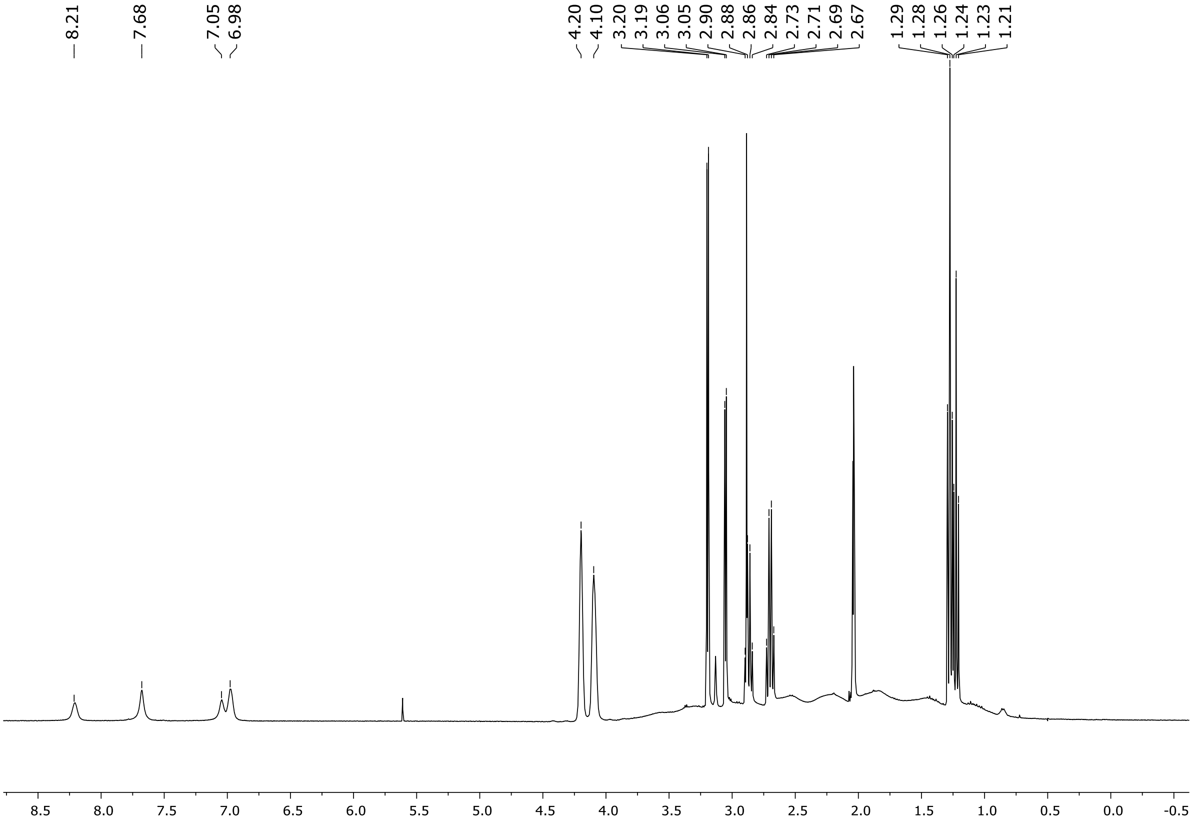

Figure S1. ^{1}H NMR spectrum of compounds **1a** and **1b** (acetone-d_6)

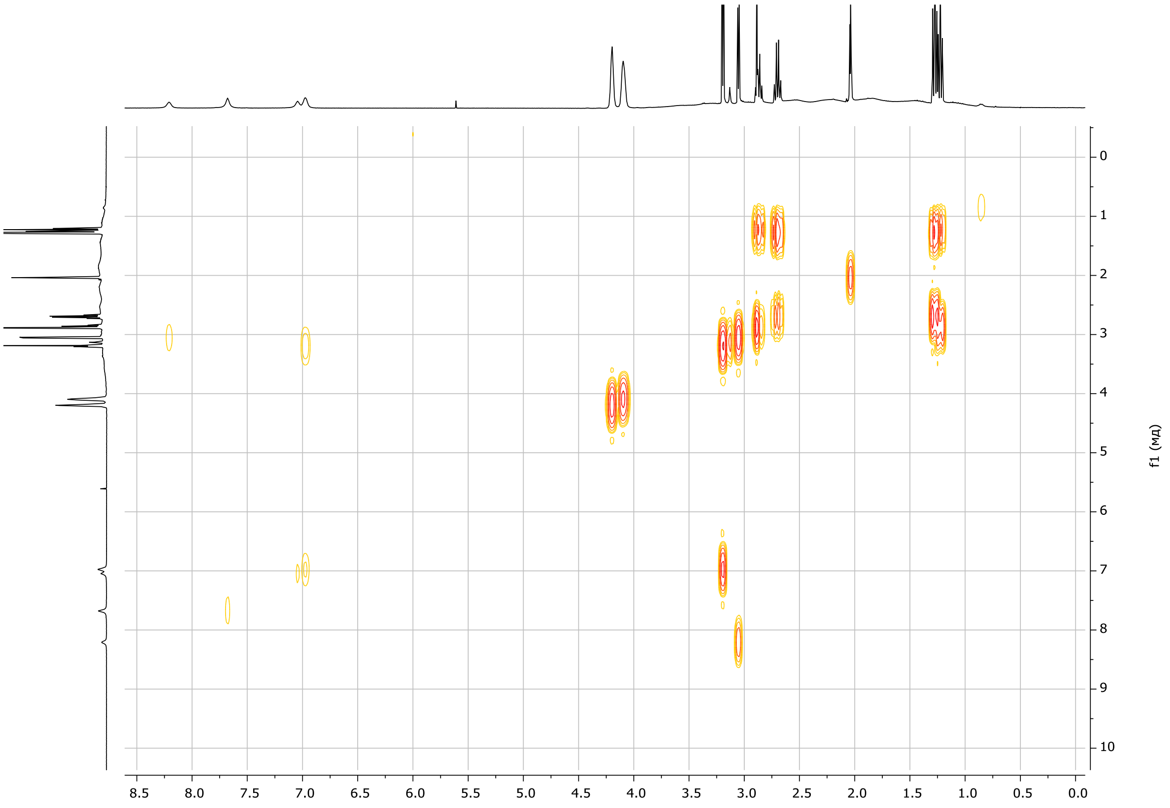

Figure S2. (HH)gCOSY NMR spectrum of compounds **1a** and **1b** (acetone-d_6)

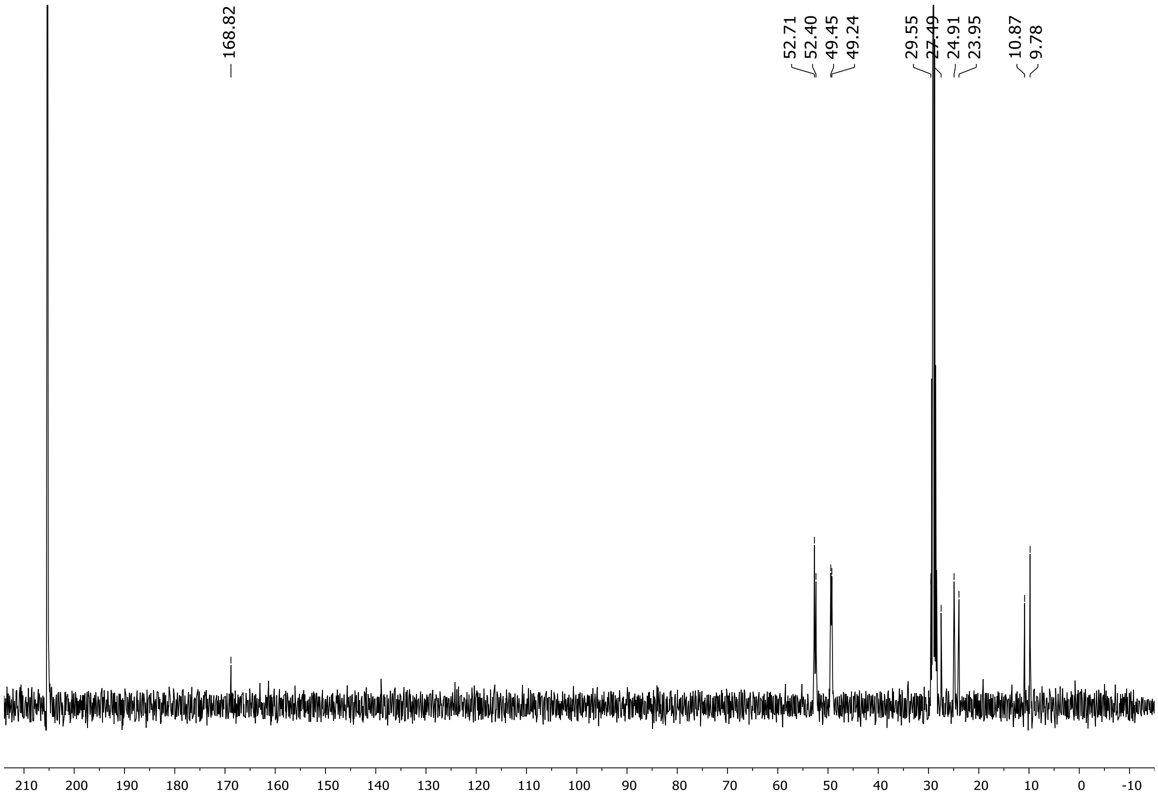

Figure S3. ^{13}C NMR spectrum of compounds **1a** and **1b** (acetone-d_6)

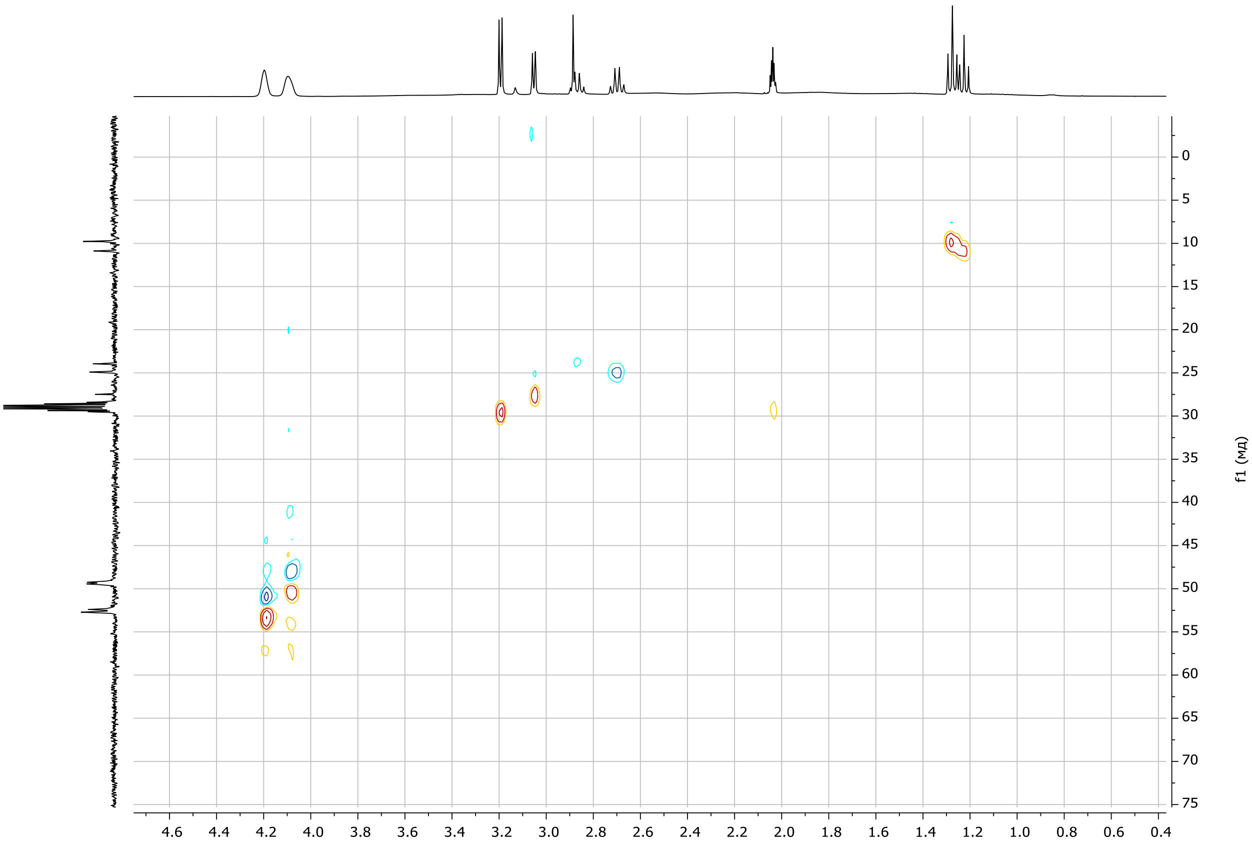

Figure S4. (HC)HSQC NMR spectrum of compounds **1a** and **1b** (acetone-d_6)

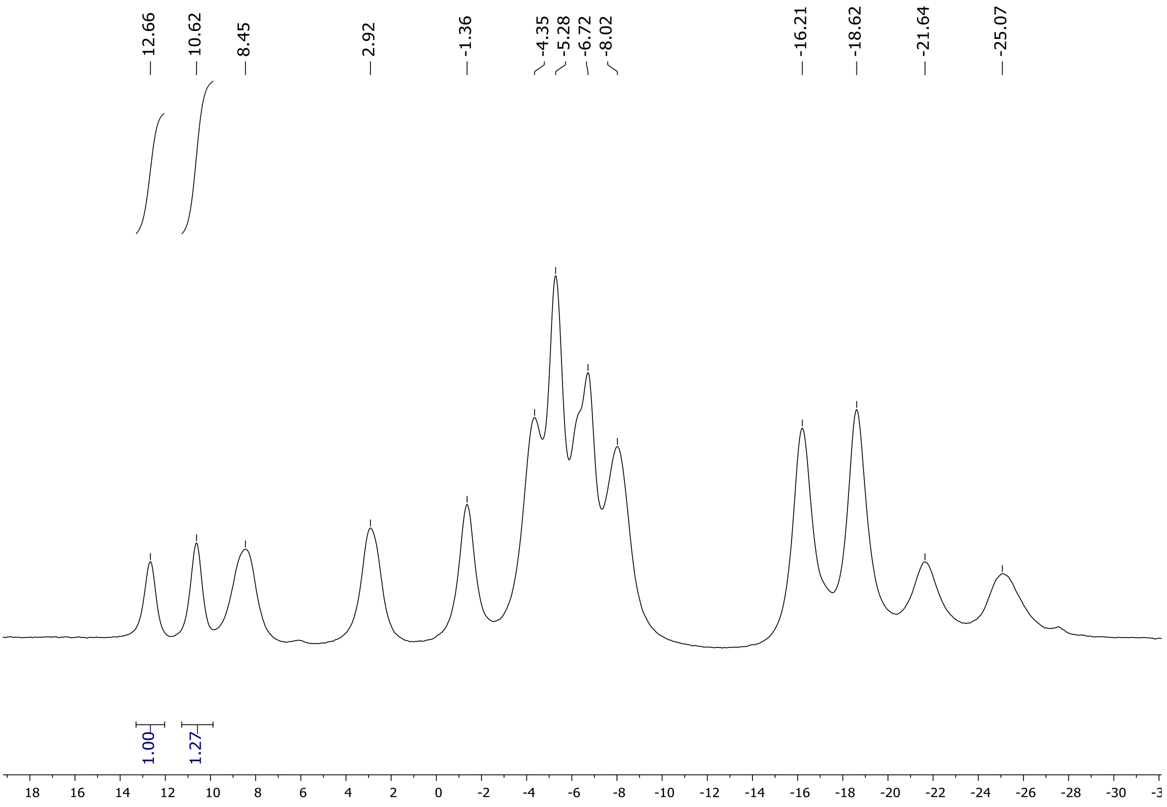

Figure S5. $^{11}B\{^{1}H\}$ NMR spectrum of compounds **1a** and **1b** (acetone-d_6)

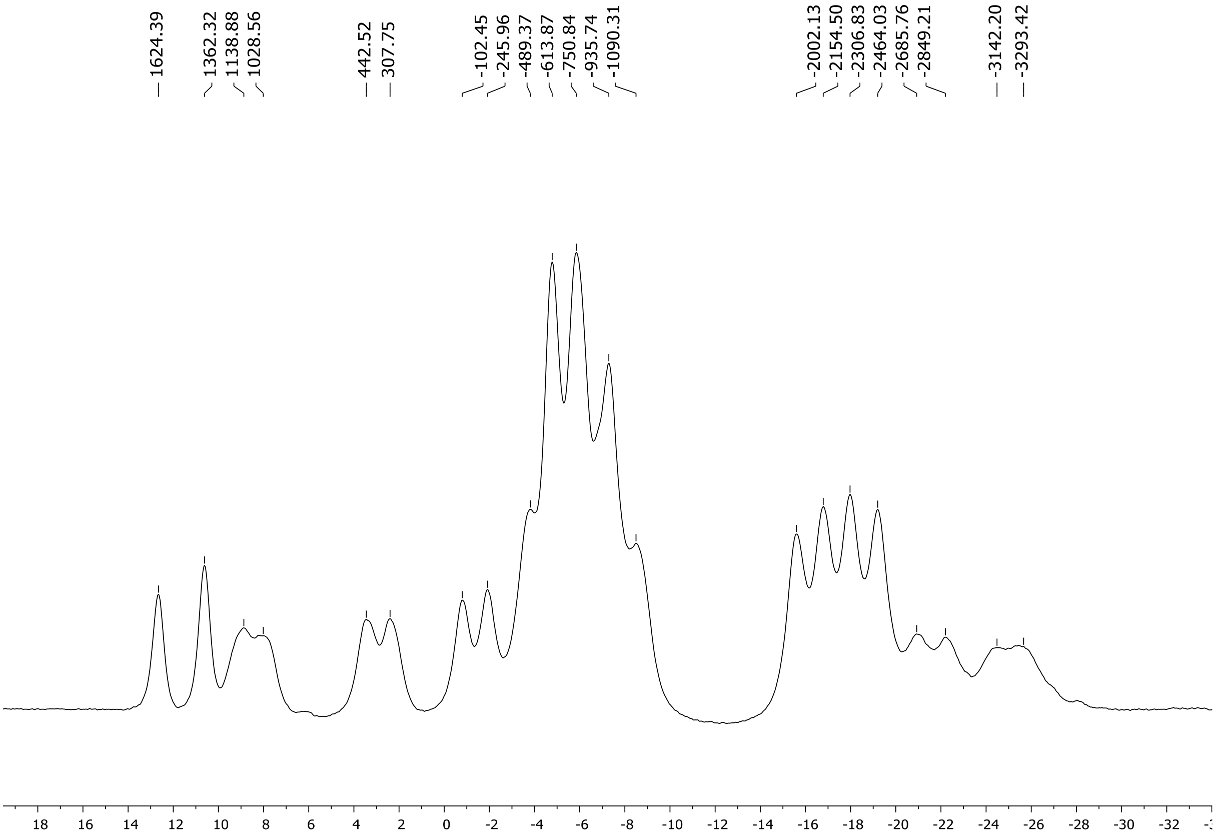

Figure S6. ^{11}B NMR spectrum of compounds **1a** and **1b** (acetone-d_6)

Spectral data for [(8-EtC(NHEt)=HN-3,3'-Co(1,2-$C_2B_9H_{10}$)(1',2'-$C_2B_9H_{11}$] (2a, 2b)

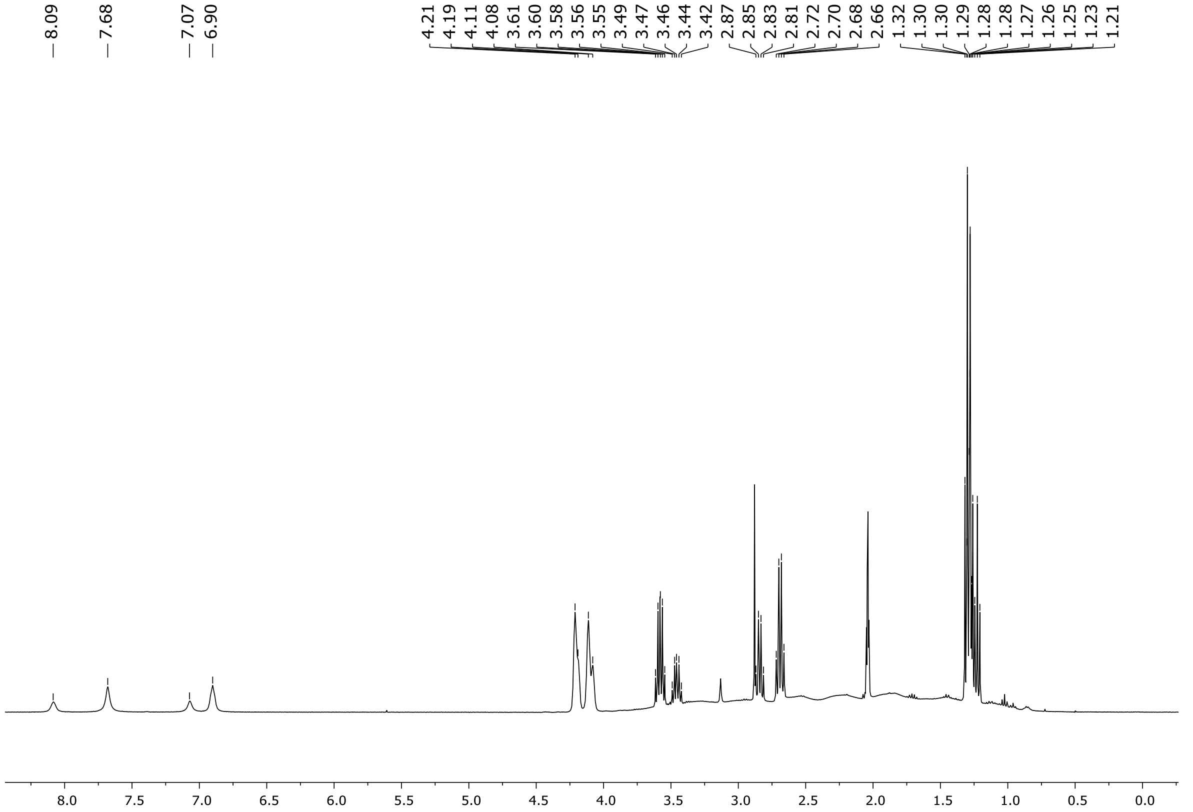

Figure S7. ^{1}H NMR spectrum of compounds **2a** and **2b** (acetone-d_6)

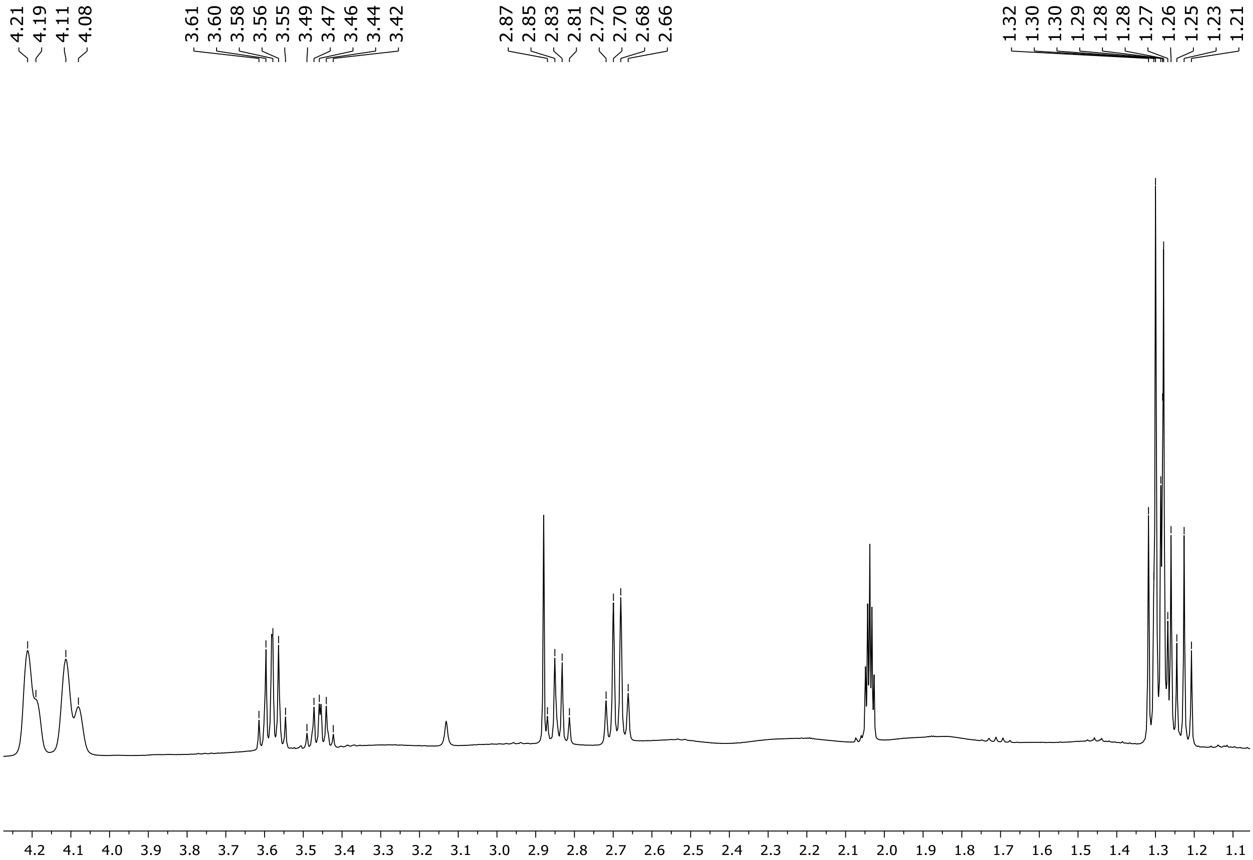

Figure S8. The fragment of ^{1}H NMR spectrum of compounds **2a** and **2b** (acetone-d_6)

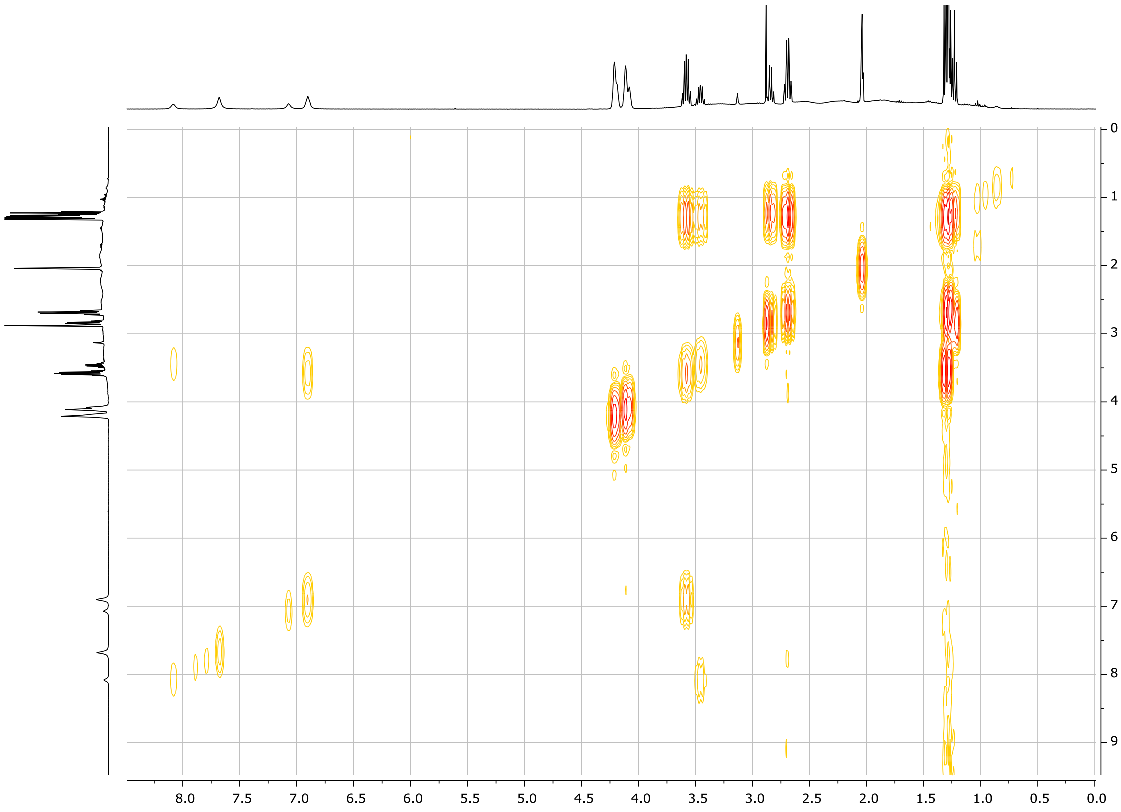

Figure S9. (HH)gCOSY NMR spectrum of compounds **2a** and **2b** (acetone-d_6)

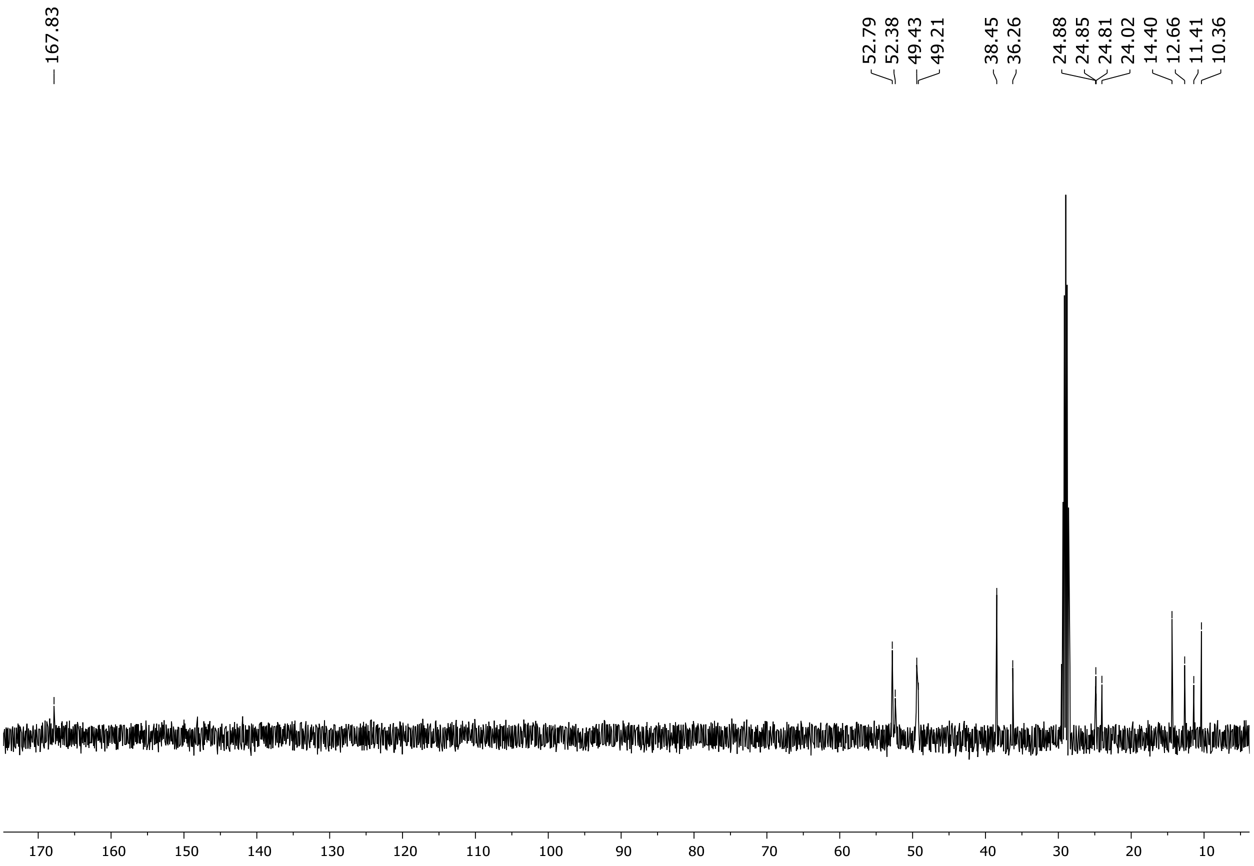

Figure S10. ^{13}C NMR spectrum of compounds **2a** and **2b** (acetone-d_6)

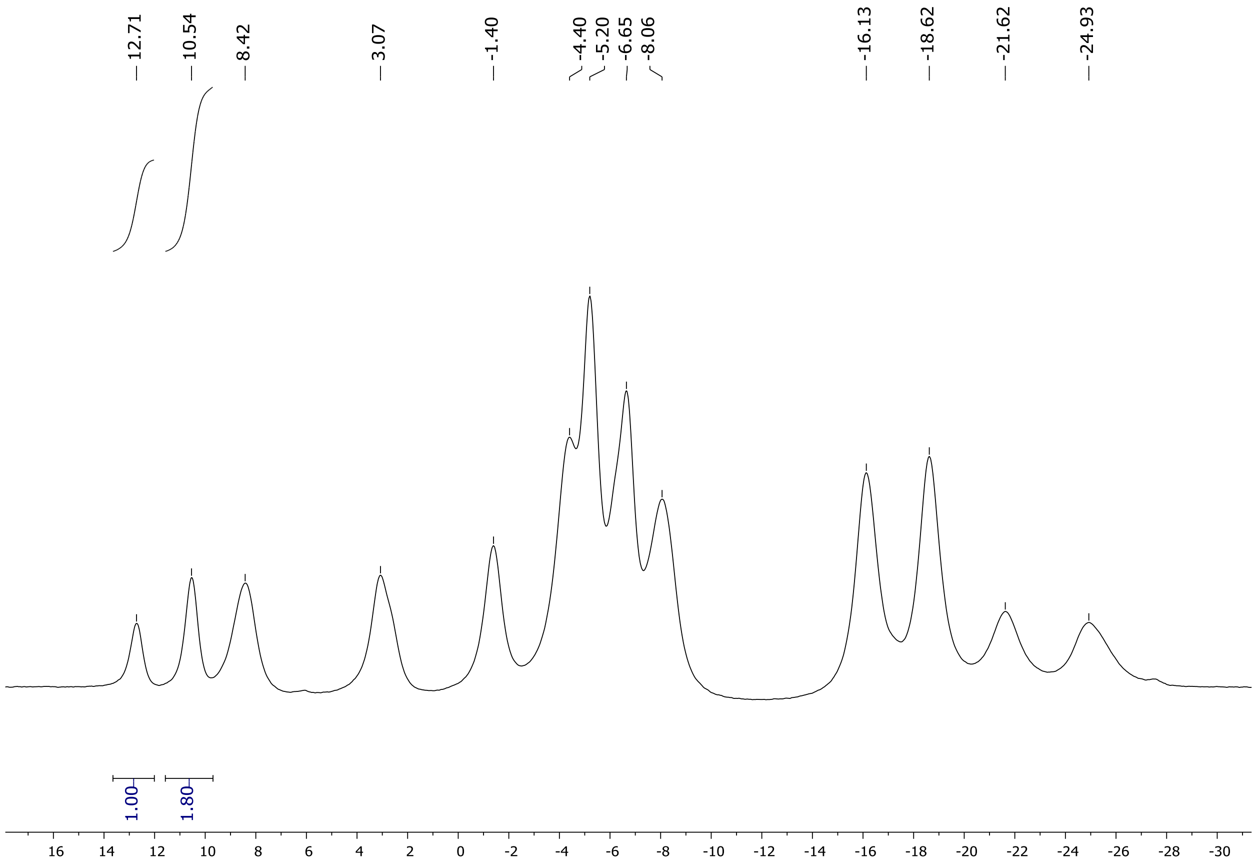

Figure S11. $^{11}B\{^{1}H\}$ NMR spectrum of compounds **2a** and **2b** (acetone-d_6)

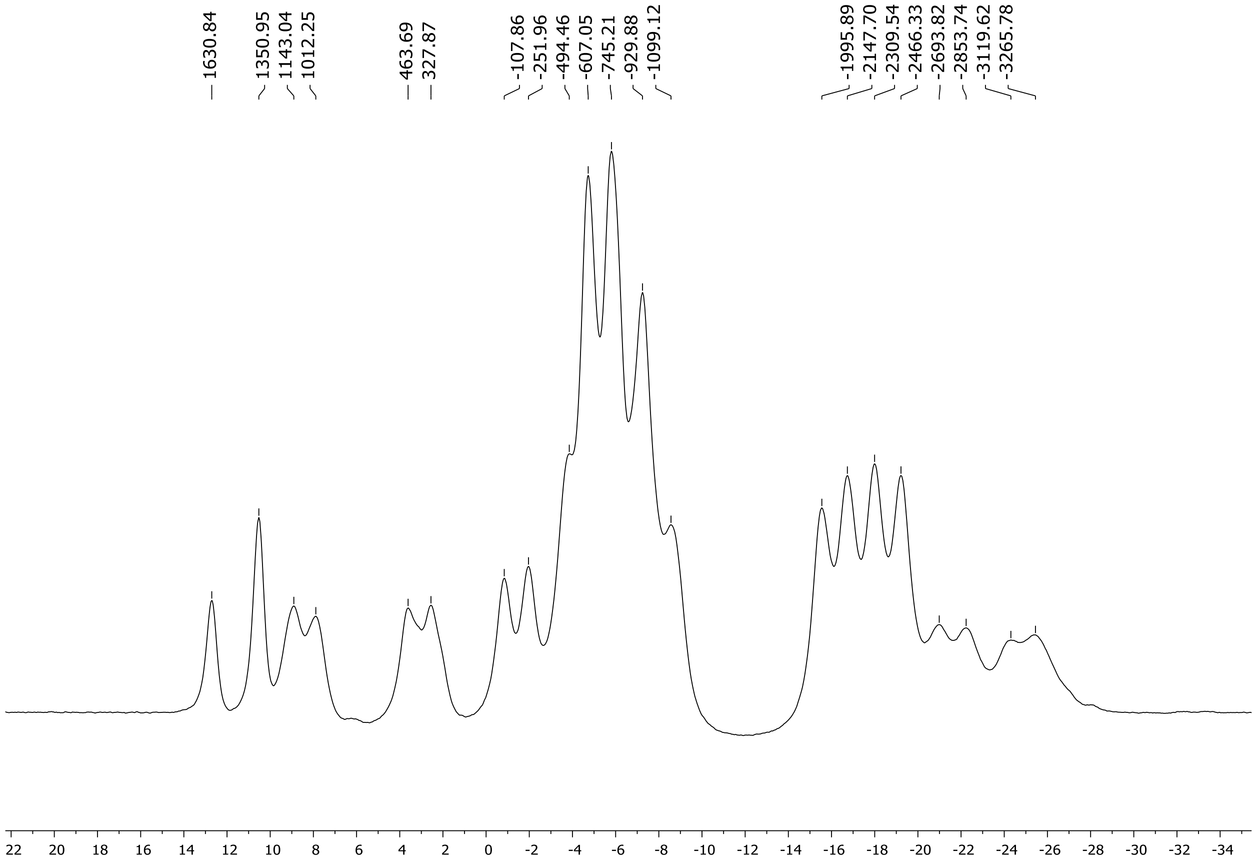

Figure S12. ^{11}B NMR spectrum of compounds **2a** and **2b** (acetone-d_6)

Spectral data for [(8-EtC(NHPr)=HN-3,3'-Co(1,2-$C_2B_9H_{10}$)(1',2'-$C_2B_9H_{11}$] (3a, 3b)

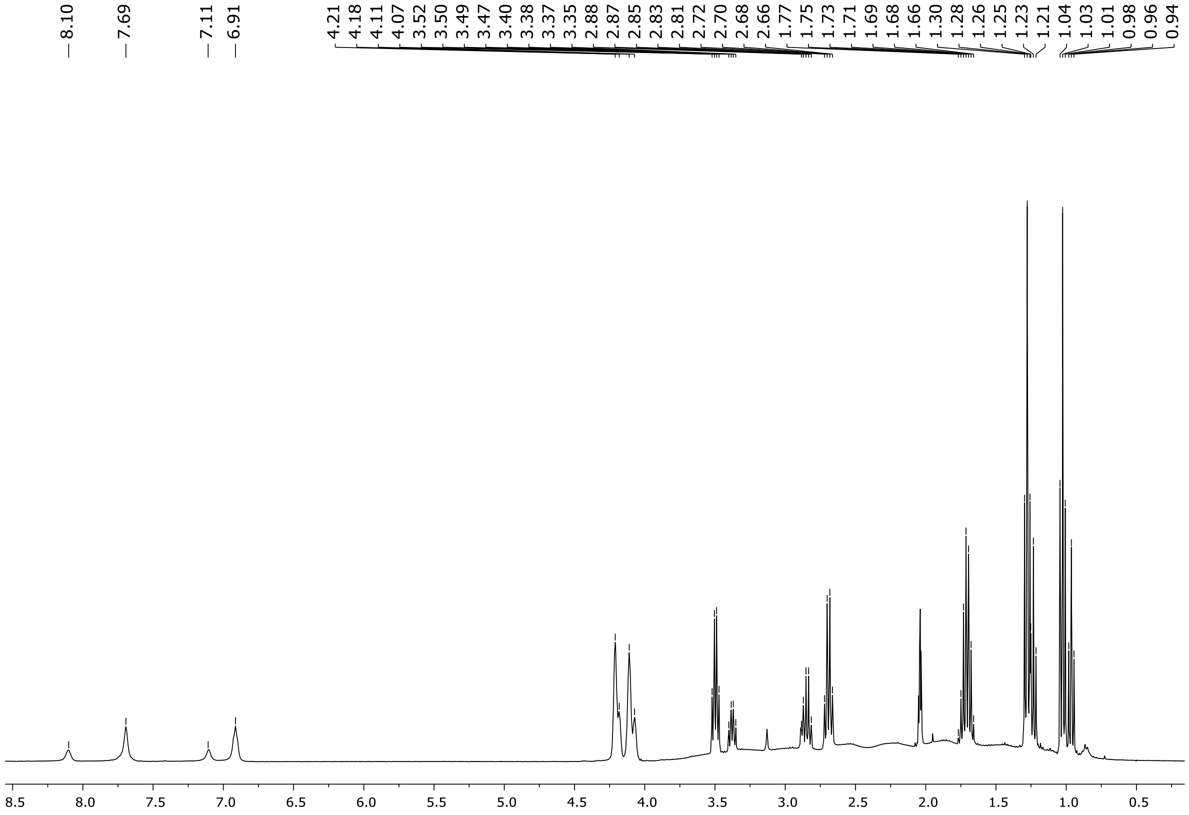

Figure S13. ^{1}H NMR spectrum of compounds **3a** and **3b** (acetone-d_6)

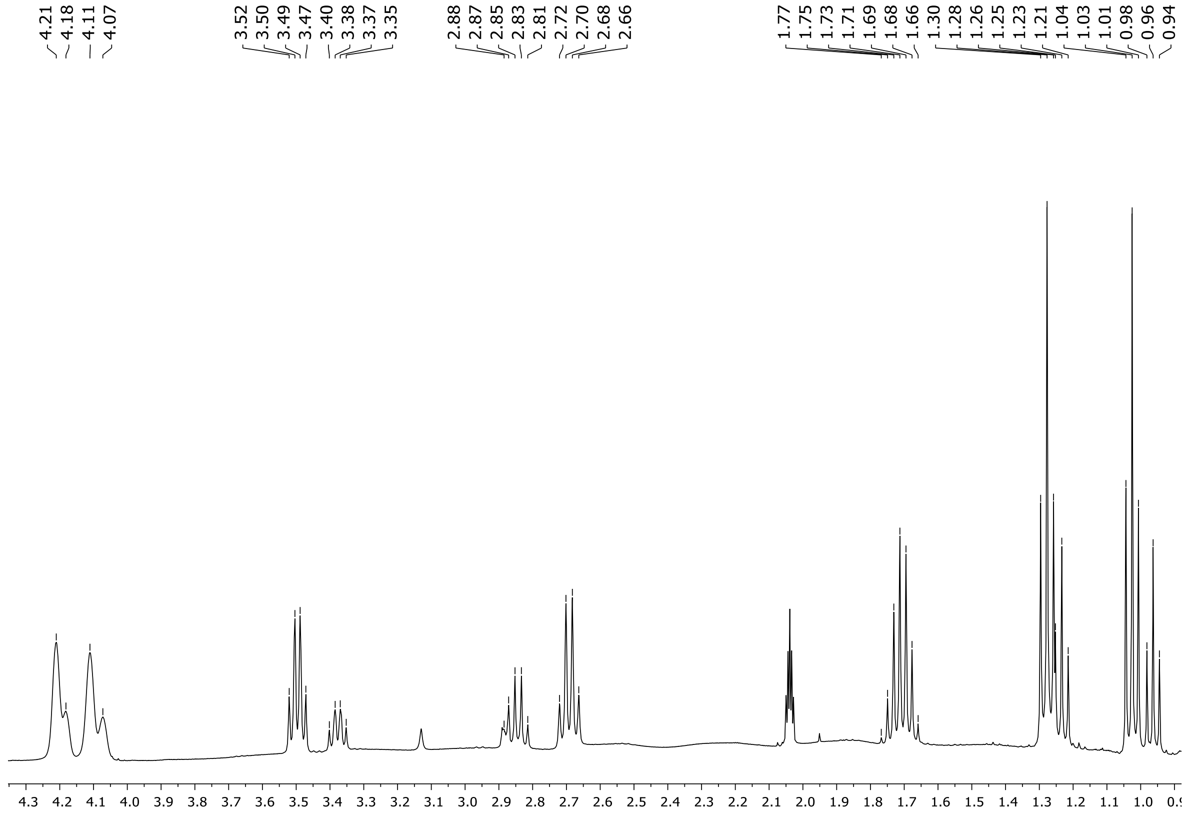

Figure S14. The fragment of ^{1}H NMR spectrum of compounds **3a** and **3b** (acetone-d_6)

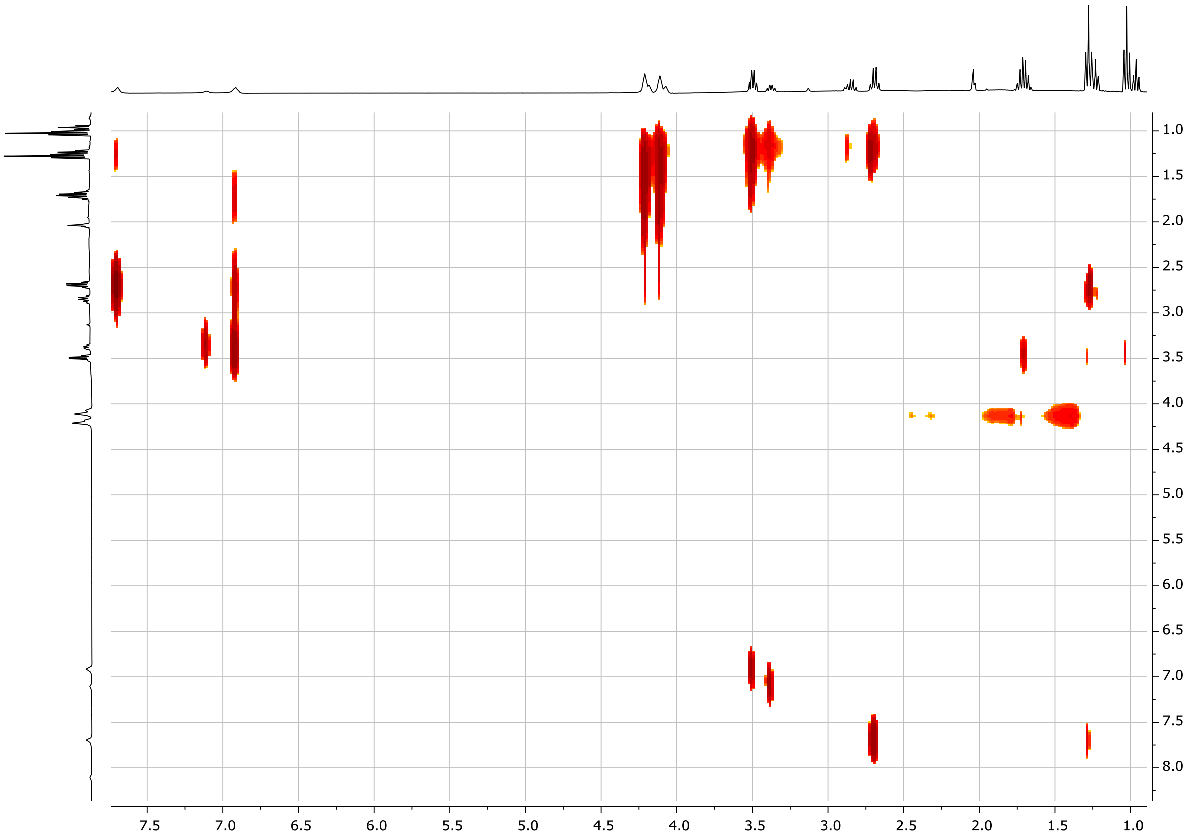

Figure S15. NOESY NMR spectrum of compounds **3a** and **3b** (acetone-d_6) with diagonal peak suppression

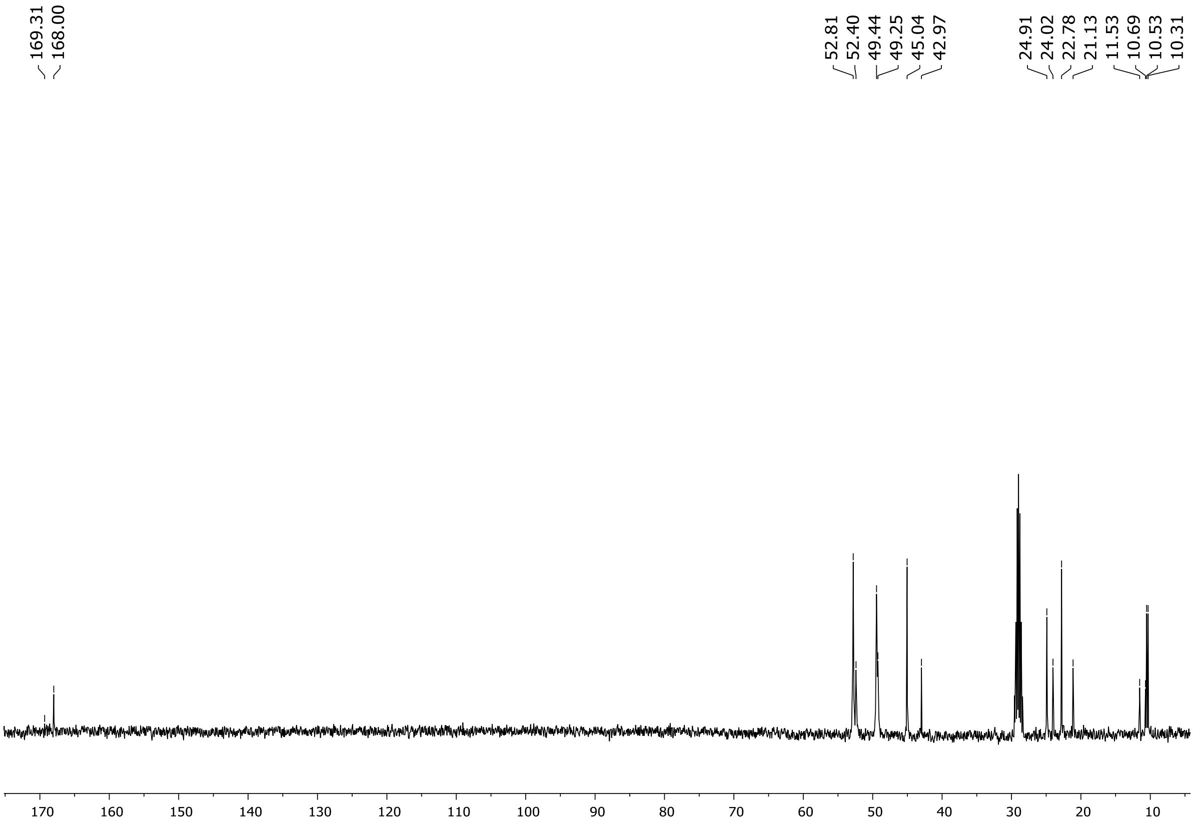

Figure S16. ^{13}C NMR spectrum of compounds **3a** and **3b** (acetone-d_6)

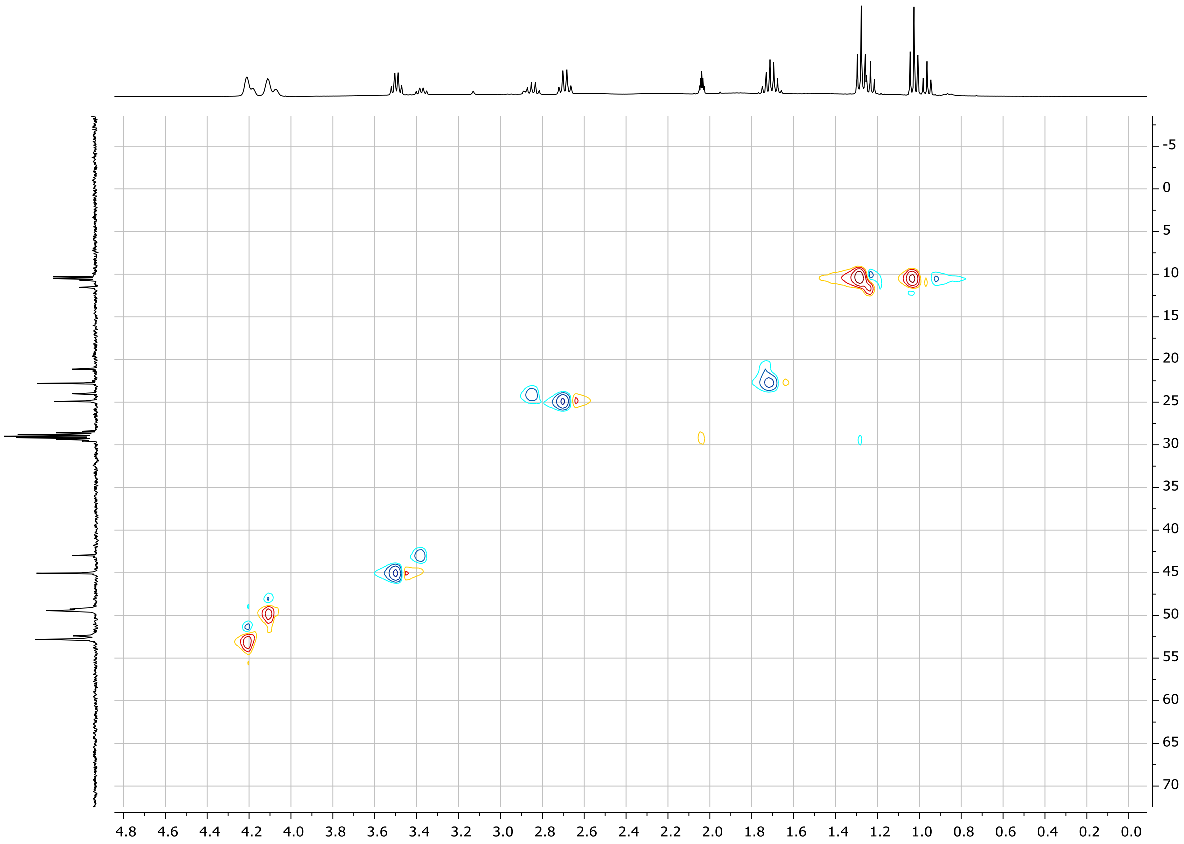

Figure S17. (HC)HSQC NMR spectrum of compounds **3a** and **3b** (acetone-d_6)

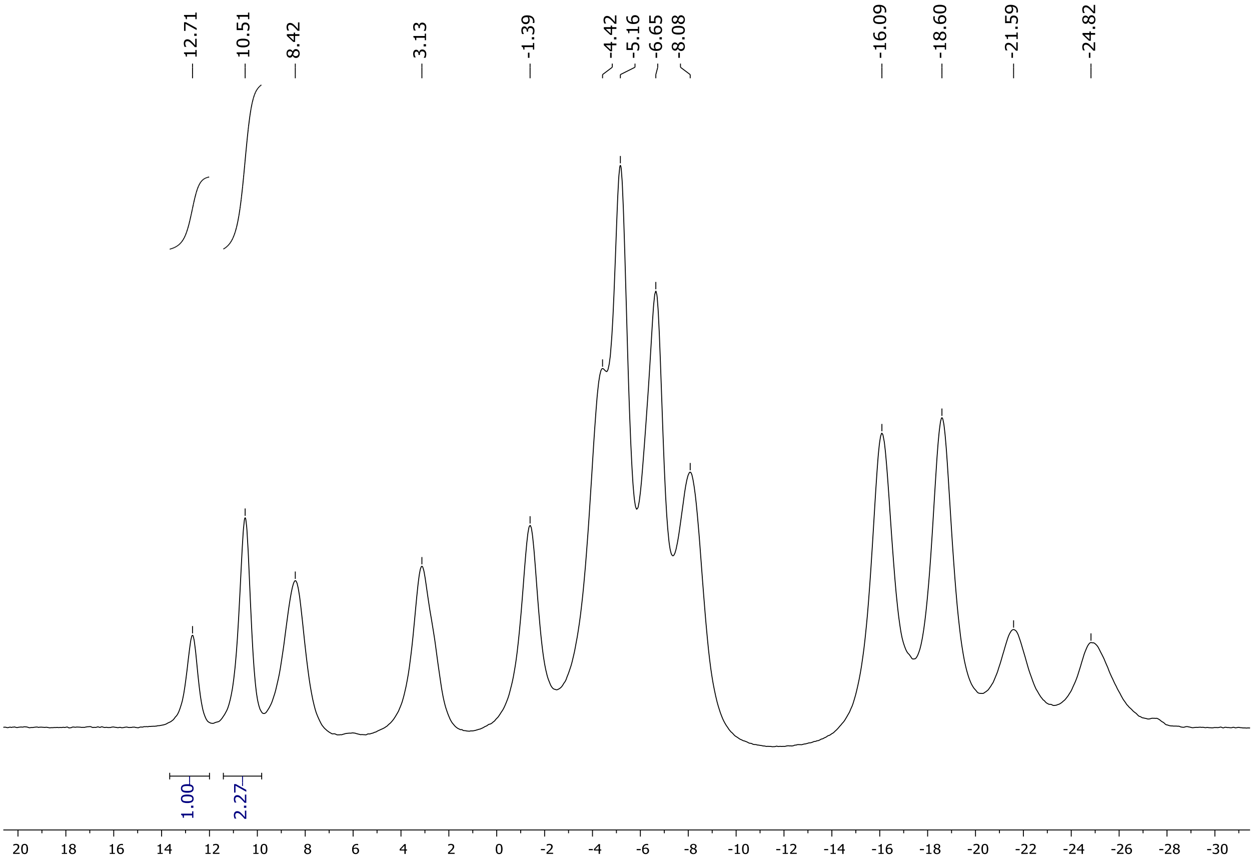

Figure S18. $^{11}B\{^1H\}$ NMR spectrum of compounds **3a** and **3b** (acetone-d_6)

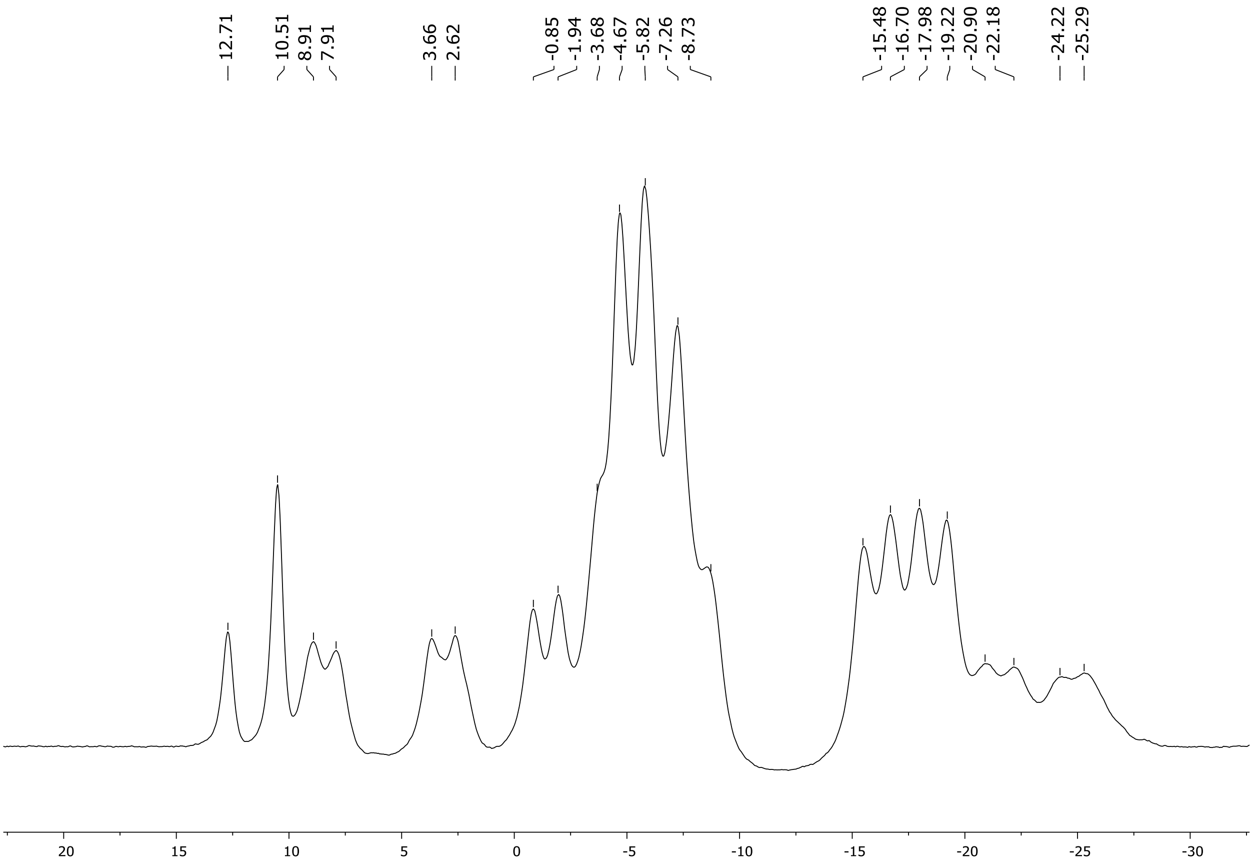

Figure S19. ^{11}B NMR spectrum of compounds **3a** and **3b** (acetone-d_6)

Spectral data for [(8-EtC(NHCH$_2$CH$_2$OMe)=HN-3,3'-Co(1,2-C$_2$B$_9$H$_{10}$)(1',2'-C$_2$B$_9$H$_{11}$] (4a, 4b)

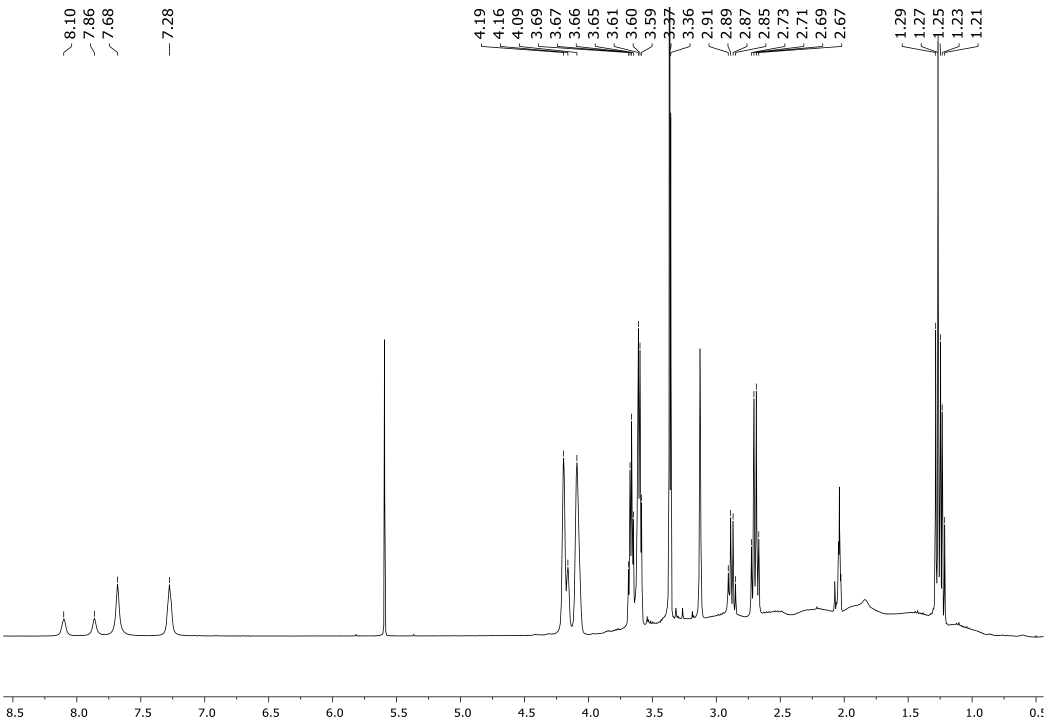

Figure S20. ^{1}H NMR spectrum of compounds **4a** and **4b** (acetone-d$_6$)

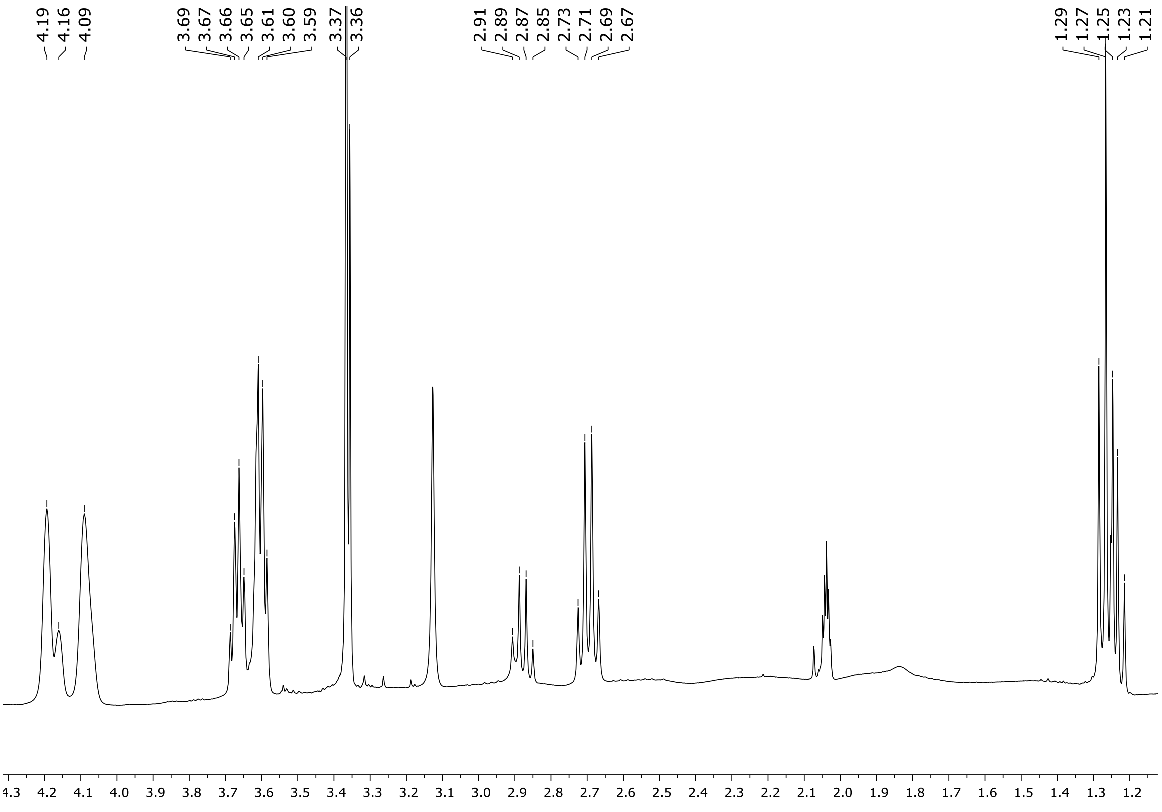

Figure S21. The fragment of ^{1}H NMR spectrum of compounds **4a** and **4b** (acetone-d$_6$)

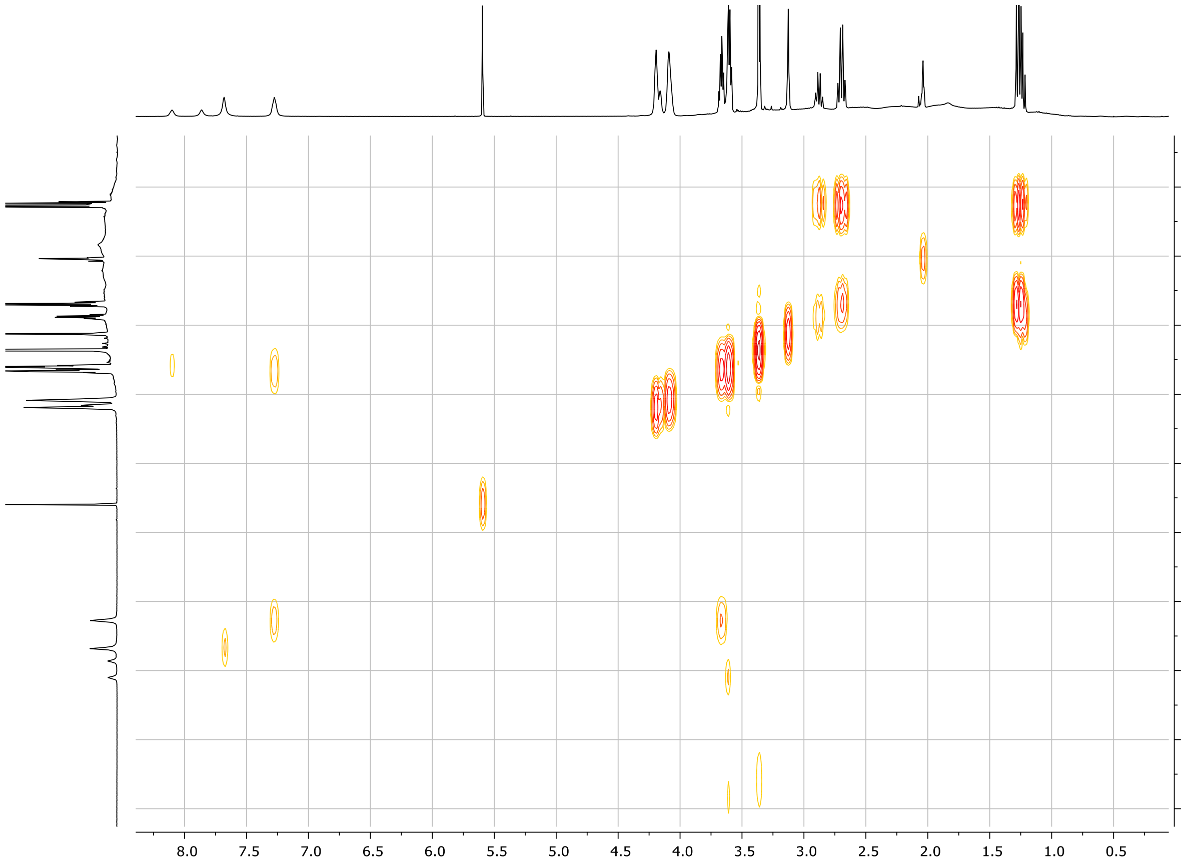

Figure S22. (HH)gCOSY NMR spectrum of compounds **4a** and **4b** (acetone-d_6)

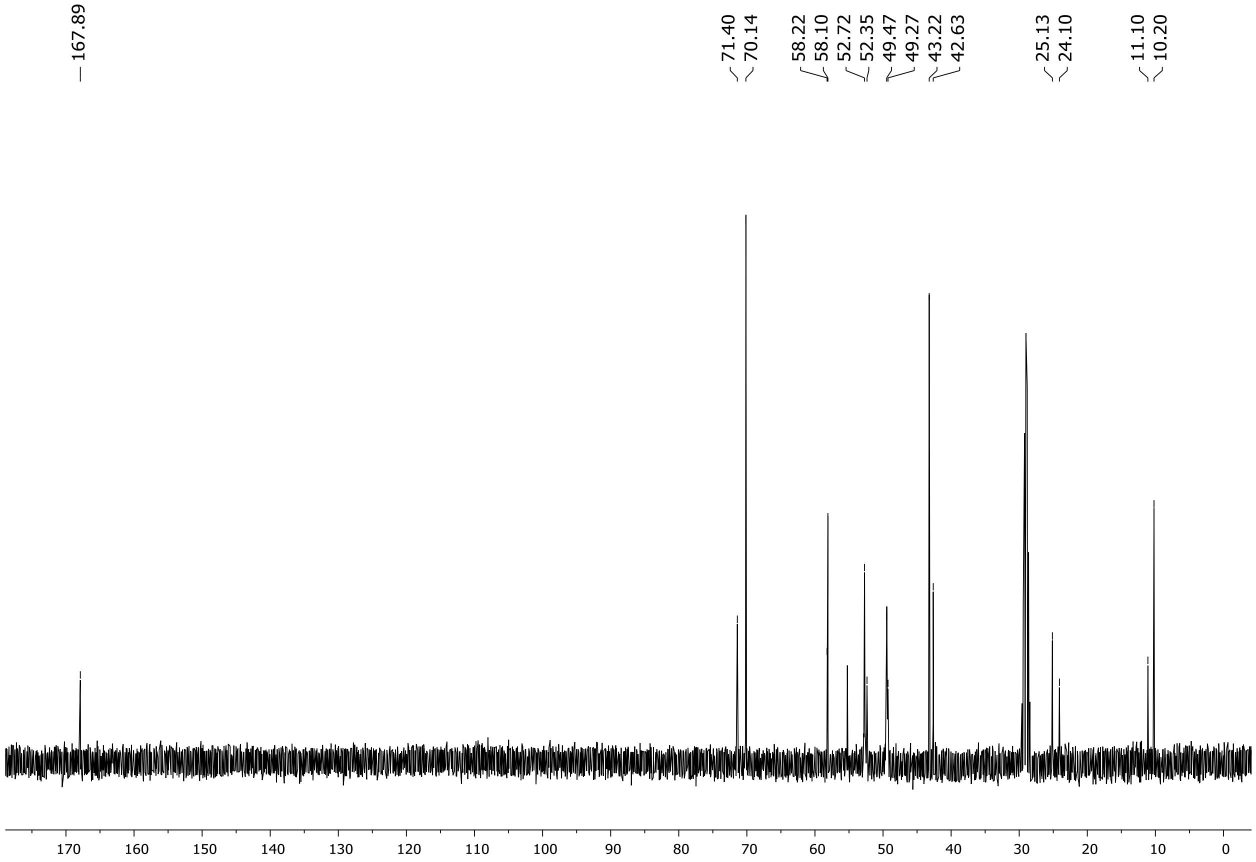

Figure S23. ^{13}C NMR spectrum of compounds **4a** and **4b** (acetone-d_6)

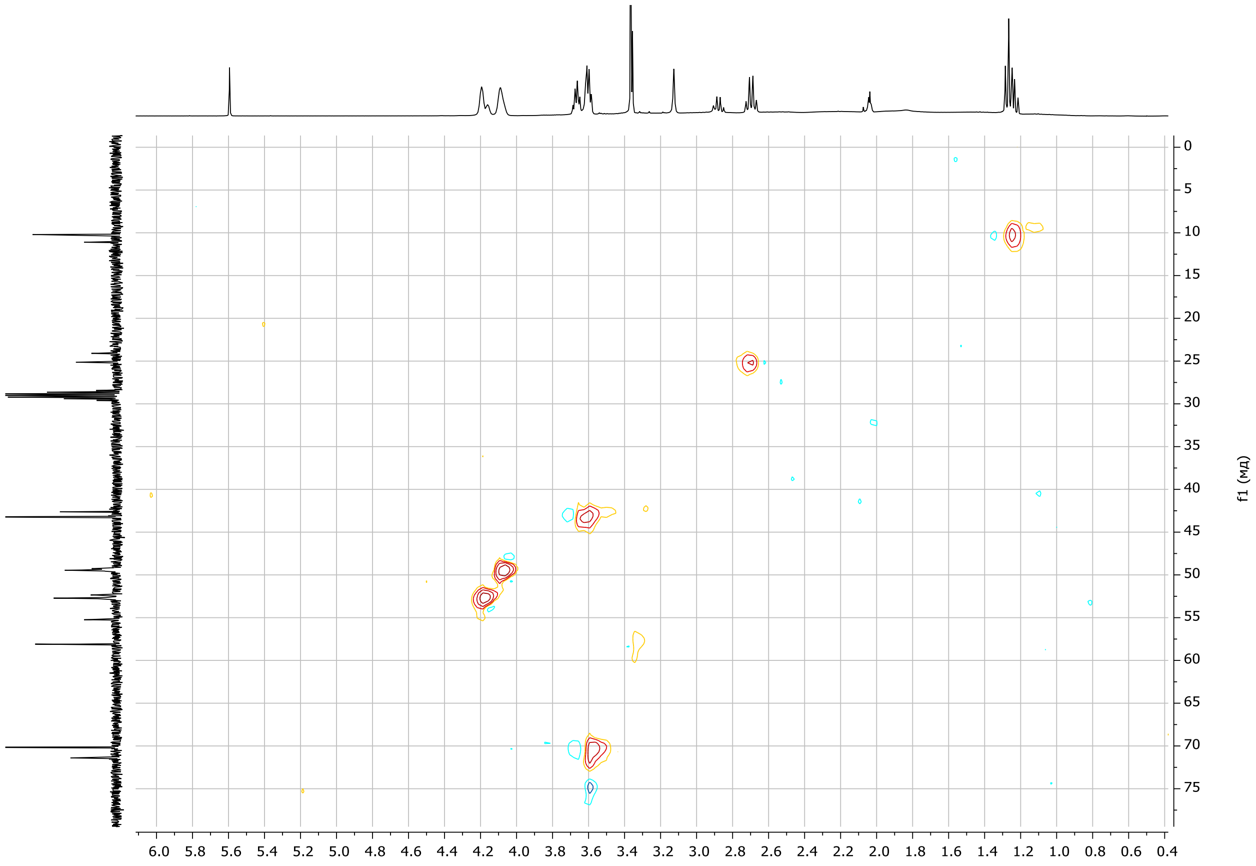

Figure S24. (HC)HSQC NMR spectrum of compounds **4a** and **4b** (acetone-d$_6$)

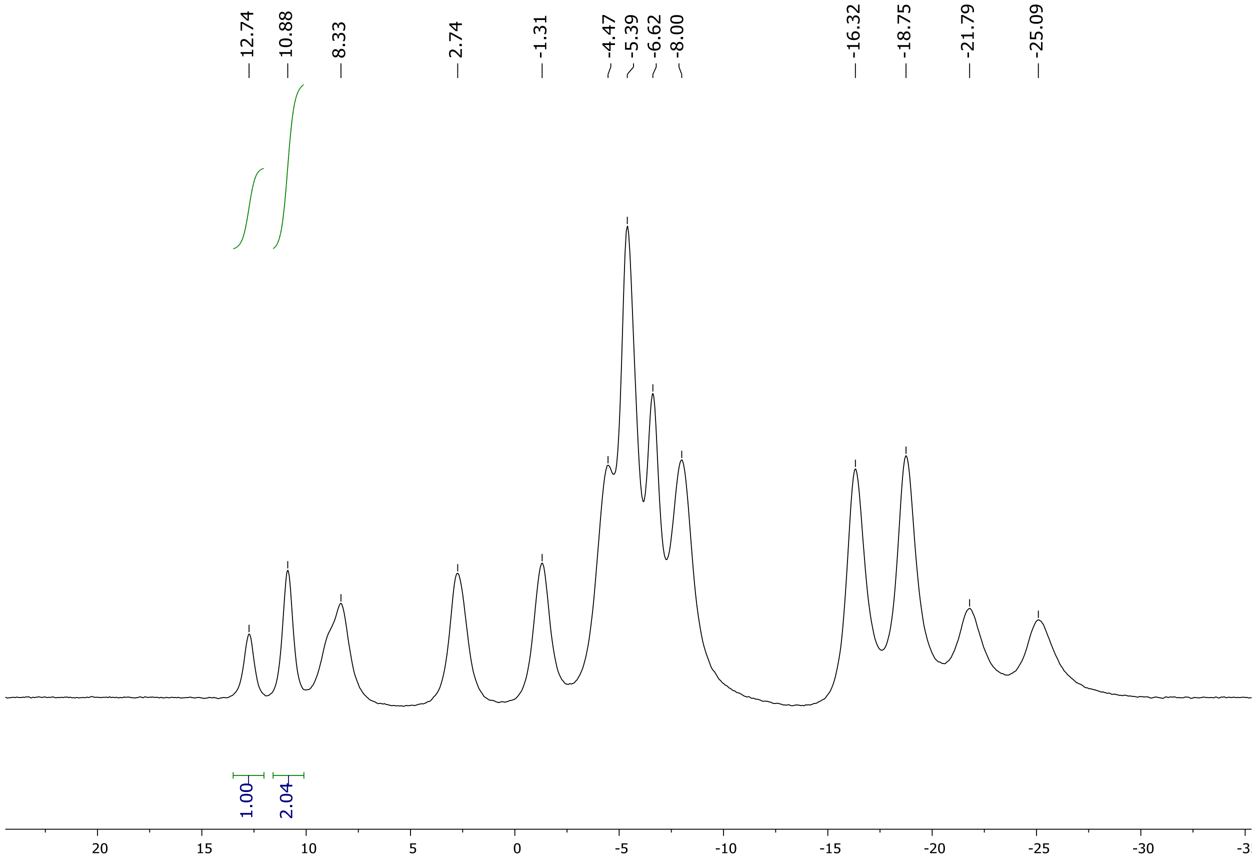

Figure S25. $^{11}B\{^1H\}$ NMR spectrum of compounds **4a** and **4b** (acetone-d$_6$)

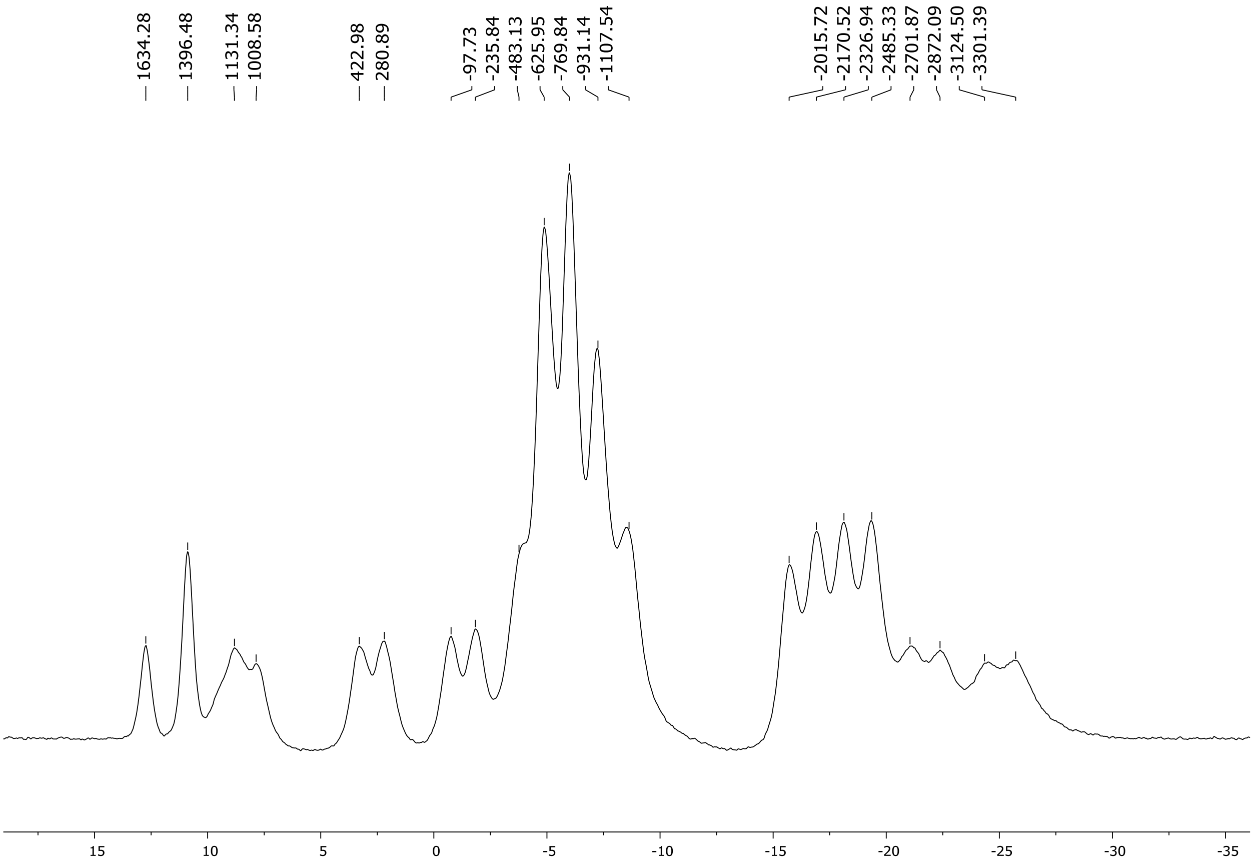

Figure S26. $^{11}B\{^1H\}$ NMR spectrum of compounds **4a** and **4b** (acetone-d_6)

Spectral data for [(8-EtC(NHCH$_2$CH$_2$CH$_2$OH)=HN-3,3'-Co(1,2-C$_2$B$_9$H$_{10}$)(1',2'-C$_2$B$_9$H$_{11}$] (5a, 5b)

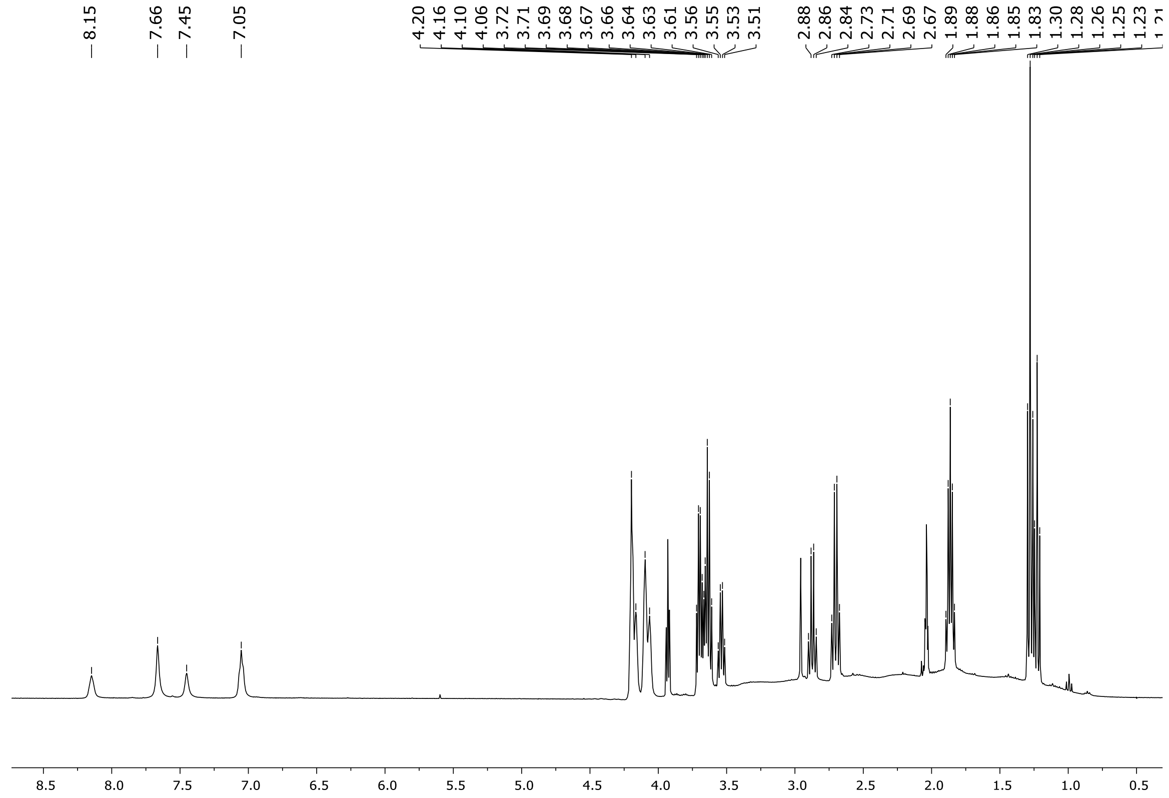

Figure S27. ^{1}H NMR spectrum of compounds **5a** and **5b** (acetone-d$_6$)

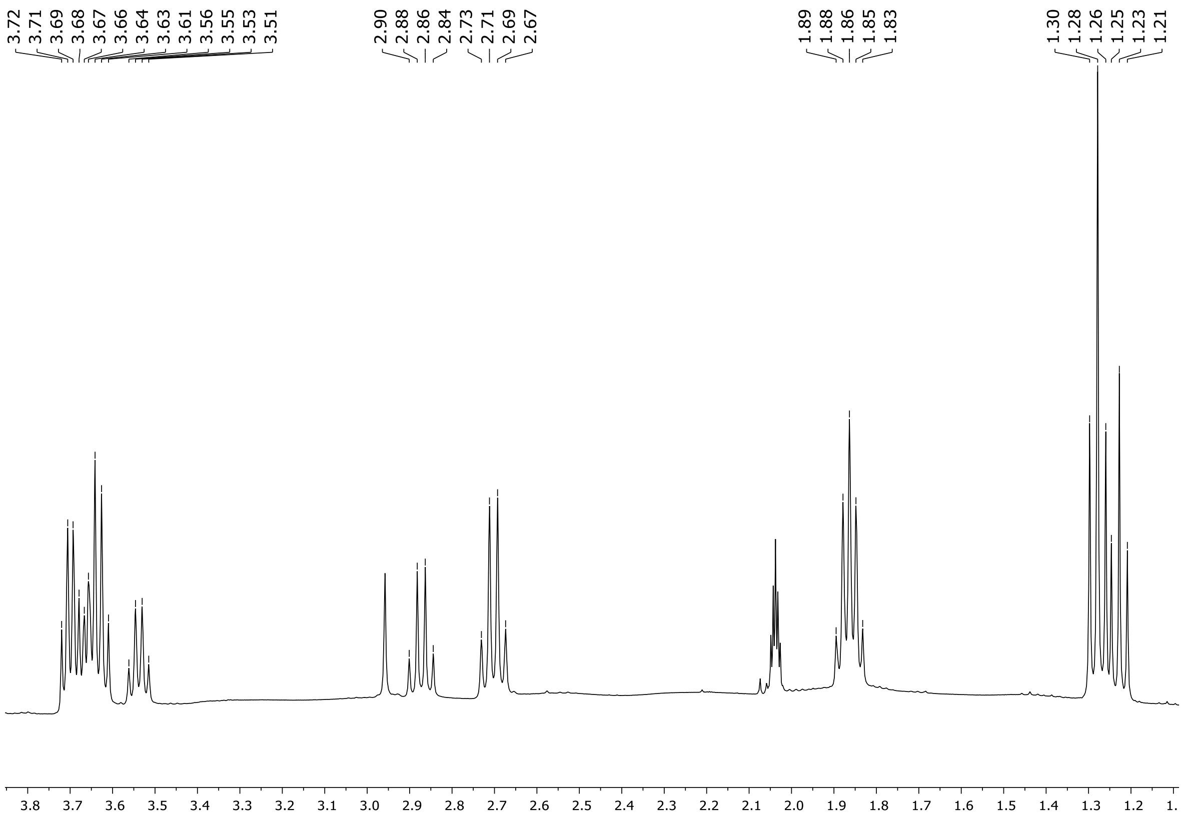

Figure S28. The fragment of ^{1}H NMR spectrum of compounds **5a** and **5b** (acetone-d$_6$)

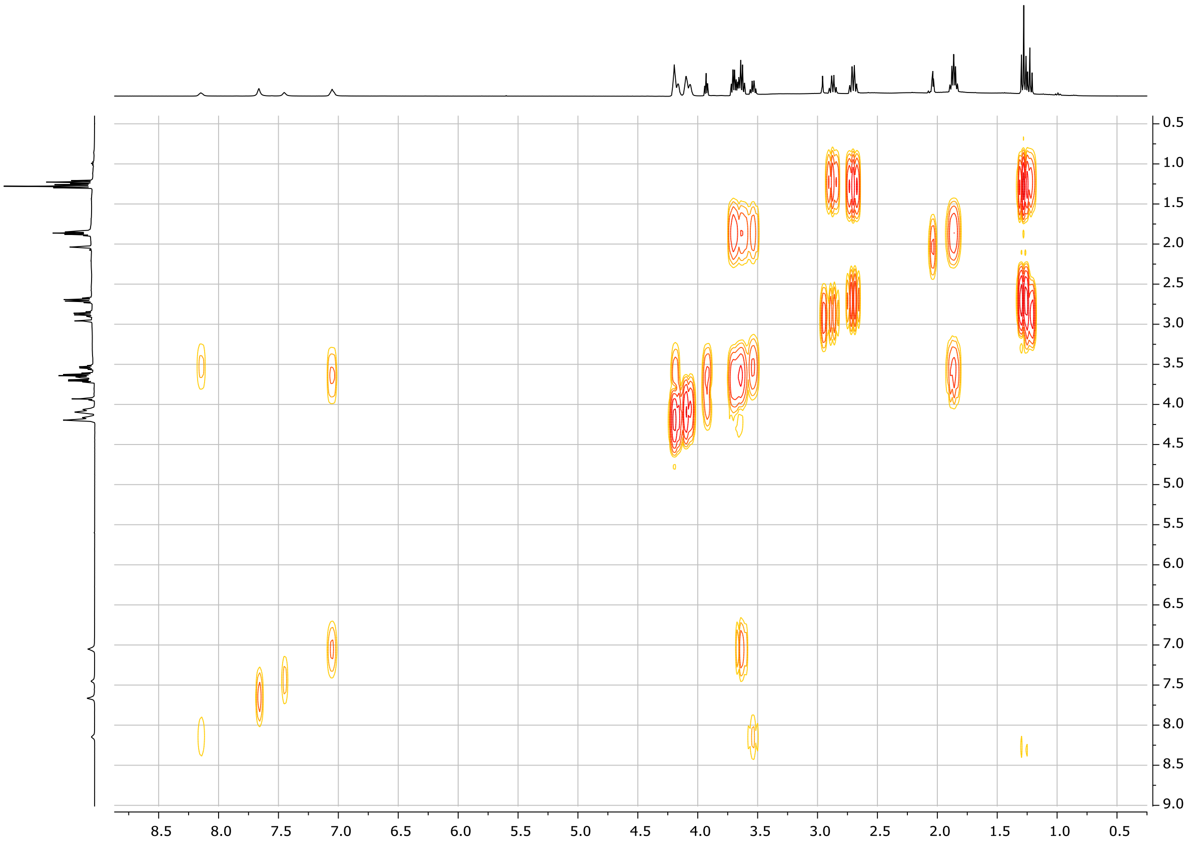

Figure S29. (HH)gCOSY NMR spectrum of compounds **5a** and **5b** (acetone-d_6)

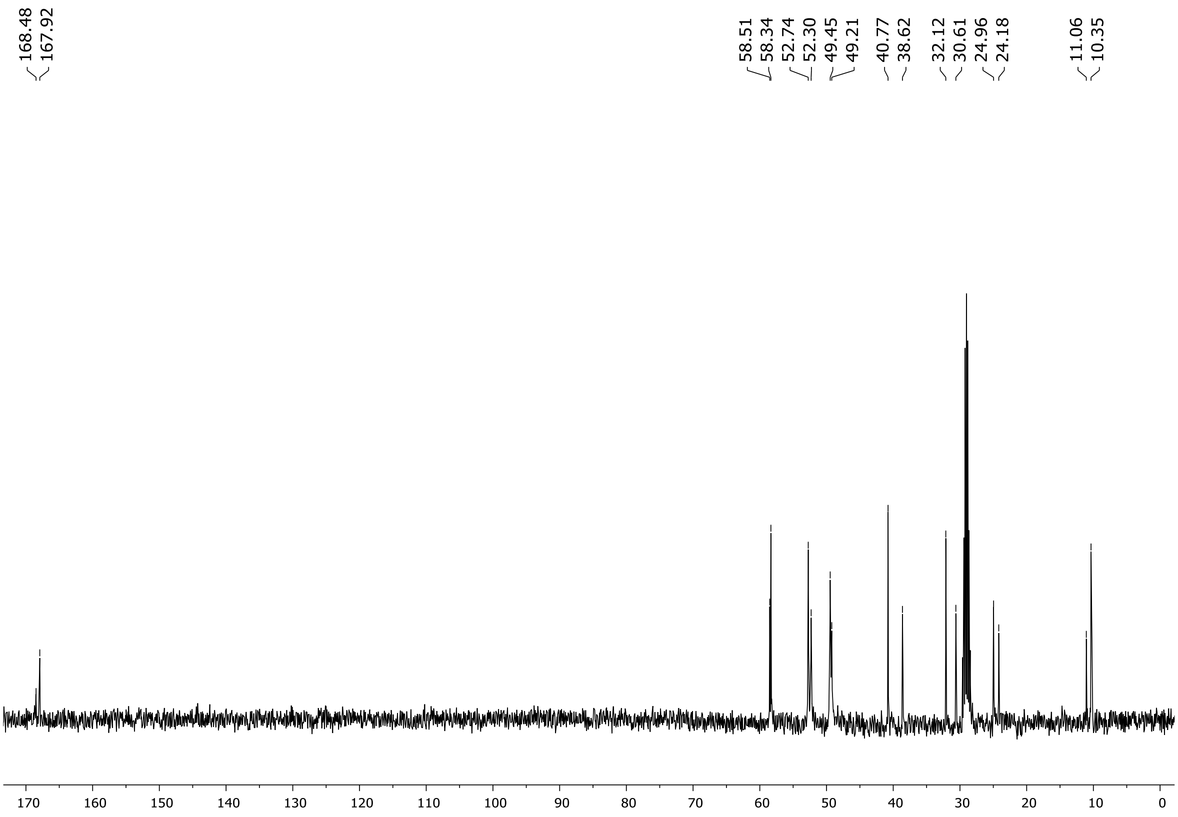

Figure S30. ^{13}C NMR spectrum of compounds **5a** and **5b** (acetone-d_6)

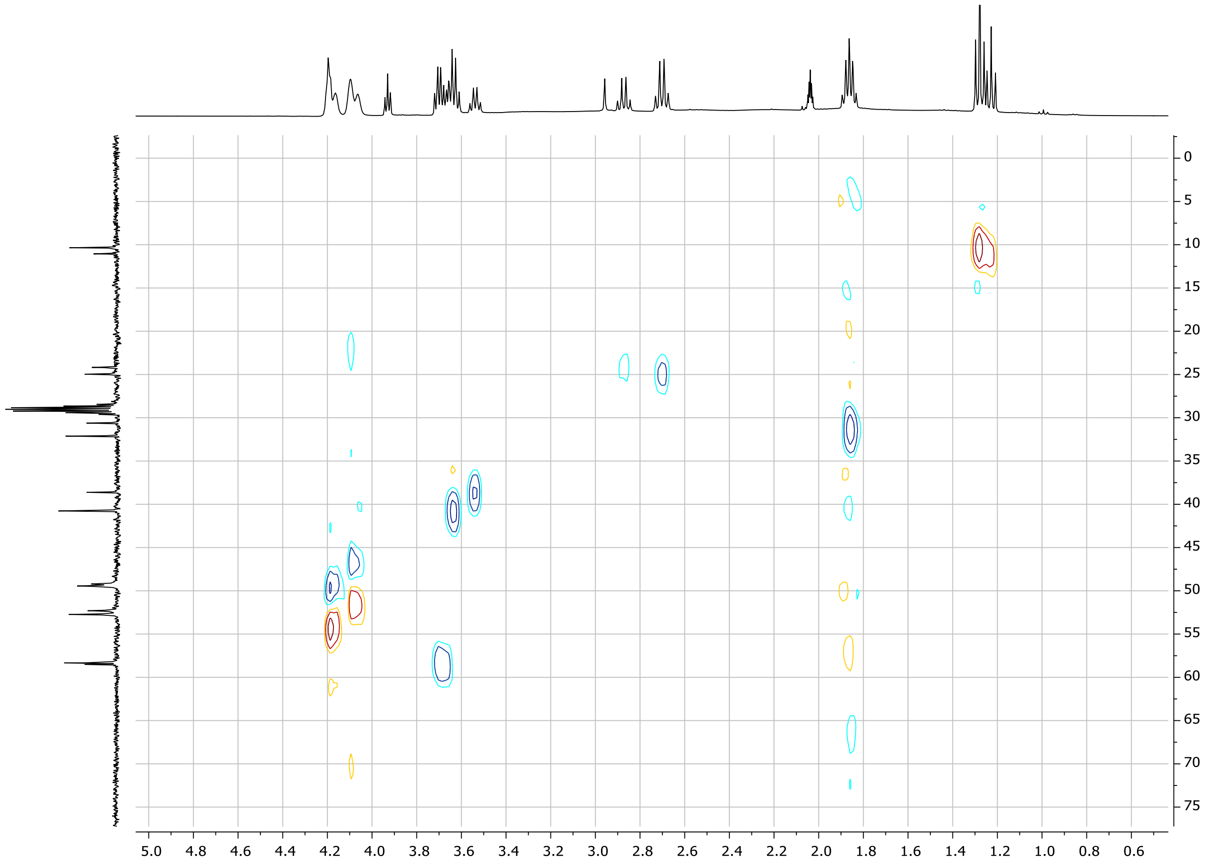

Figure S31. (HC)HSQC NMR spectrum of compounds **5a** and **5b** (acetone-d_6)

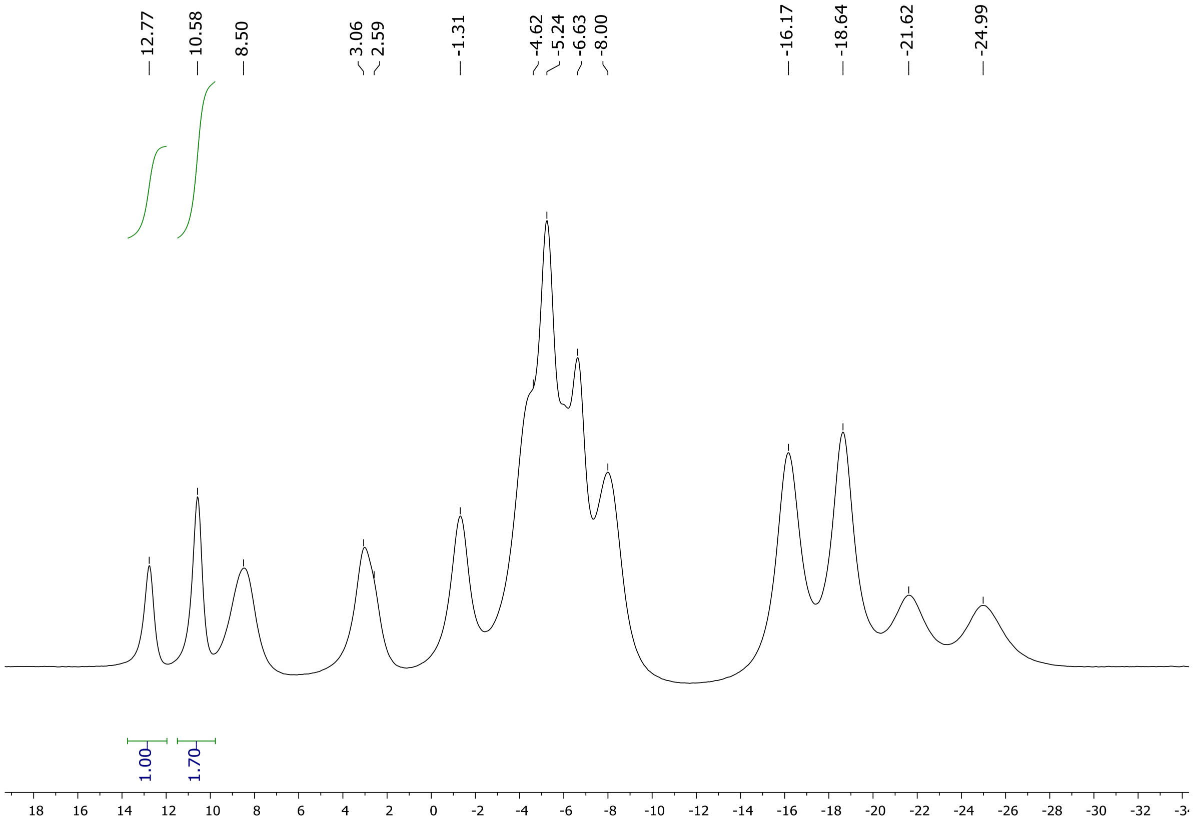

Figure S32. $^{11}B\{^{1}H\}$ NMR spectrum of compounds **5a** and **5b** (acetone-d_6)

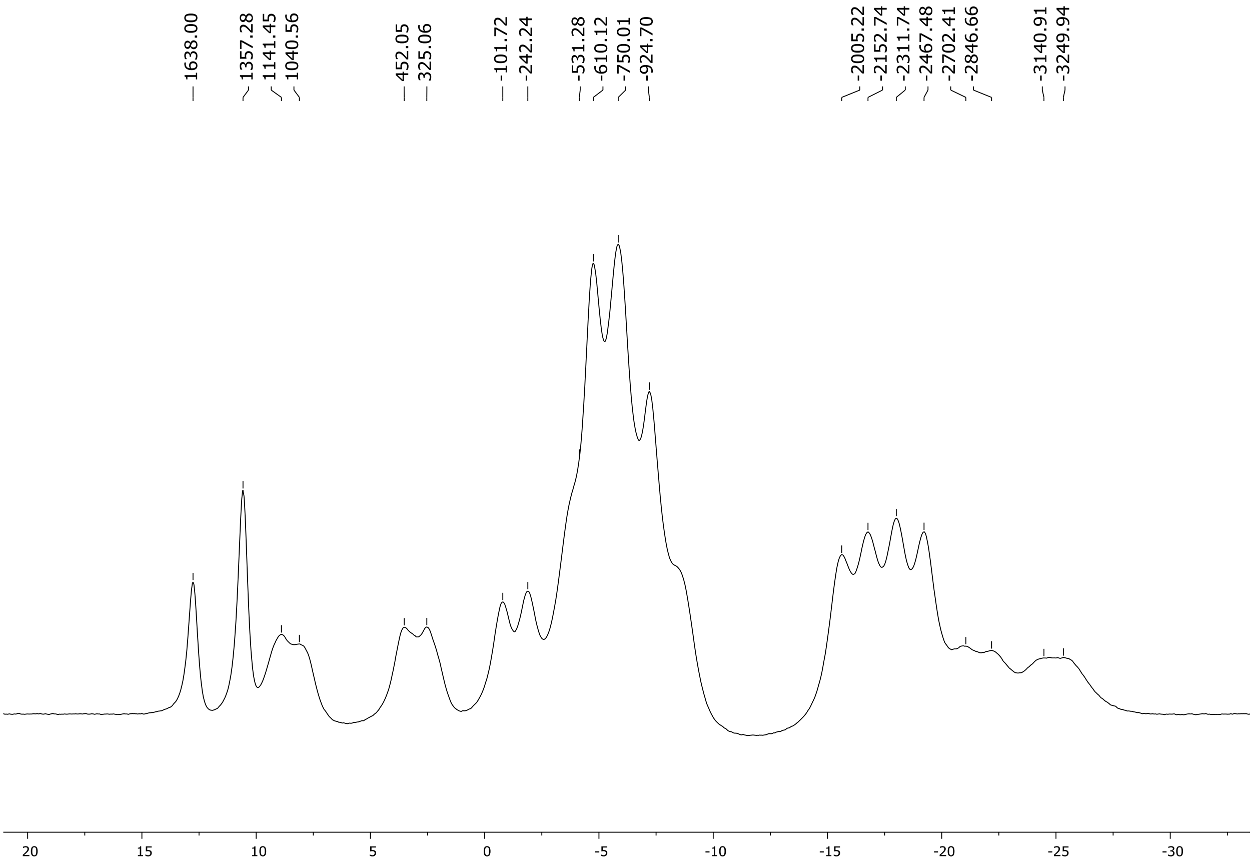

Figure S33. ^{11}B NMR spectrum of compounds **5a** and **5b** (acetone-d_6)

Spectral data for [(8-EtC(NHCH2CH2NMe2)=HN-3,3'-Co(1,2-C2B9H10)(1',2'-C2B9H11] (6a, 6b)

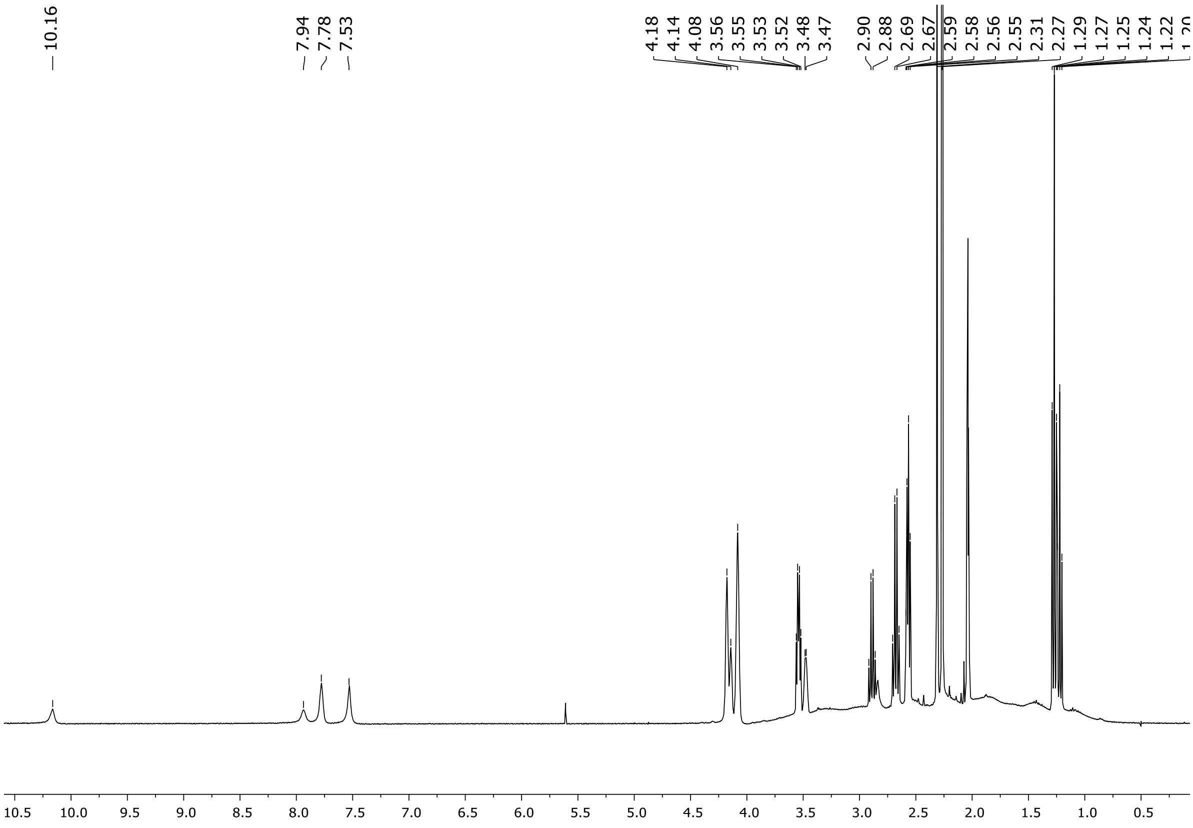

Figure S34. ^{1}H NMR spectrum of compounds **6a** and **6b** (acetone-d_6)

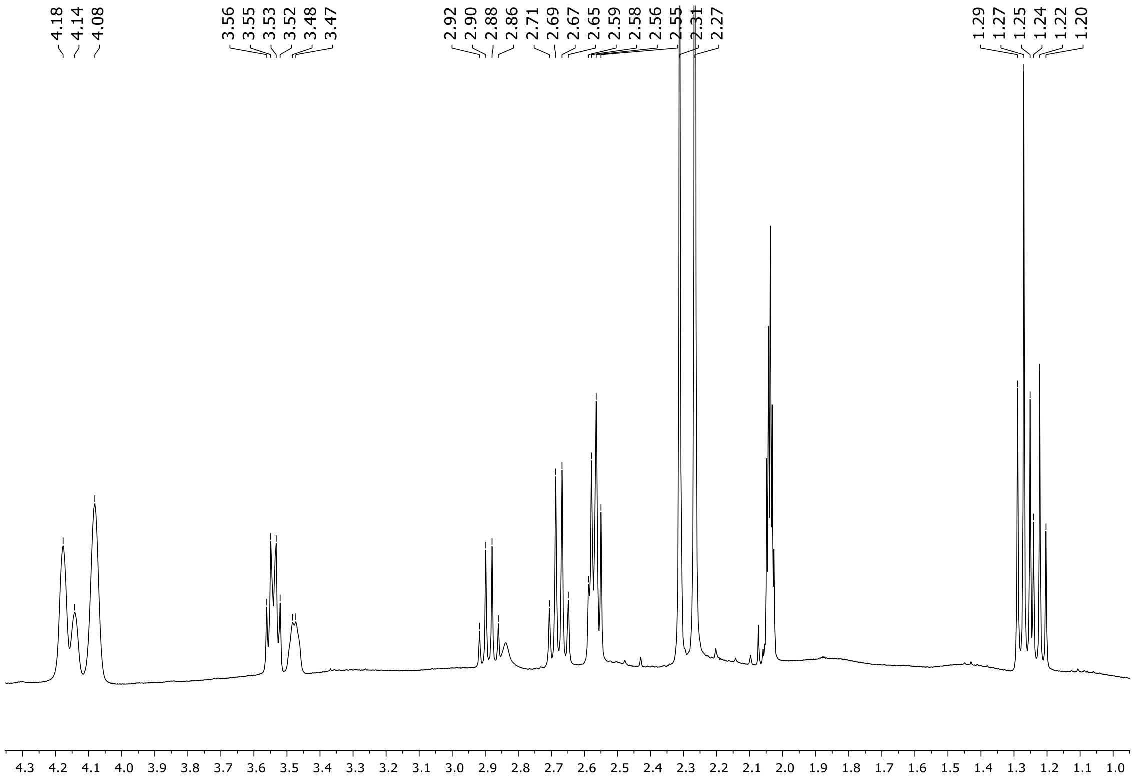

Figure S35. The fragment of ^{1}H NMR spectrum of compounds **6a** and **6b** (acetone-d_6)

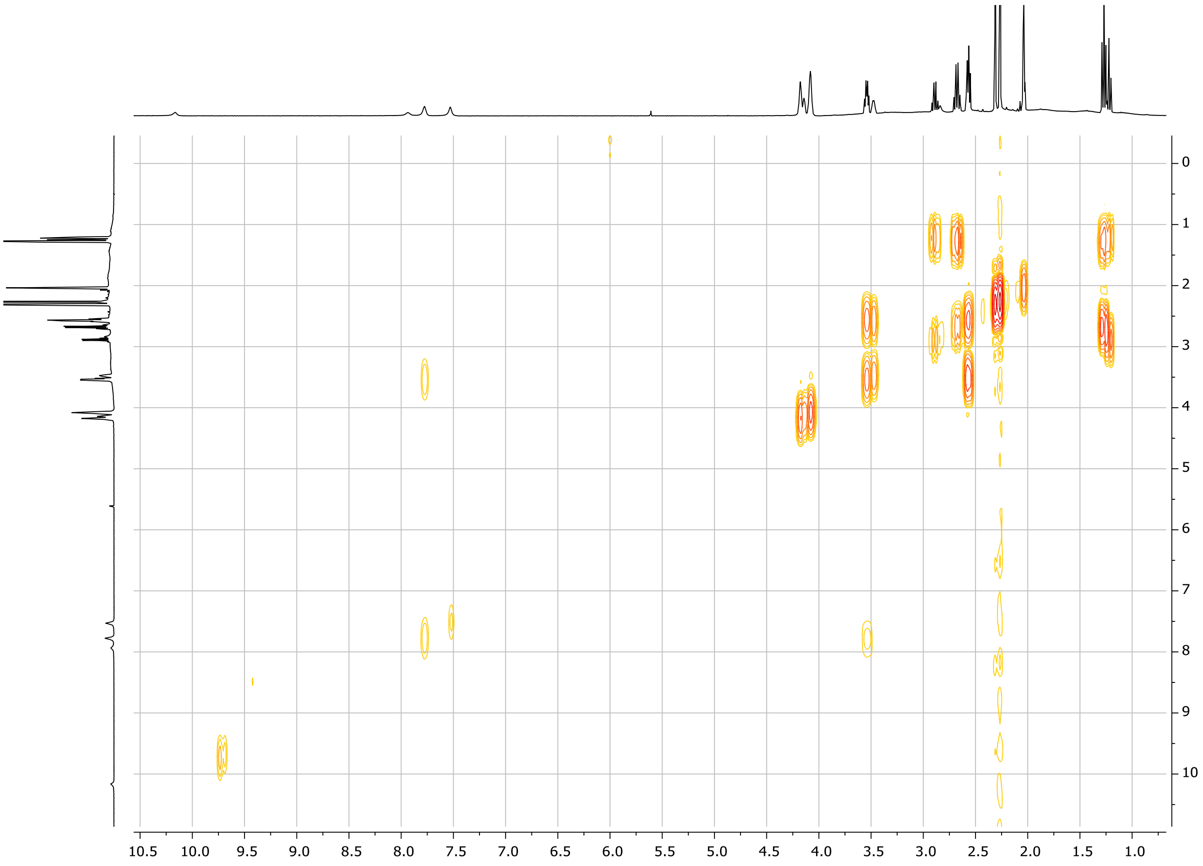

Figure S36. (HH)gCOSY NMR spectrum of compounds **6a** and **6b** (acetone-d_6)

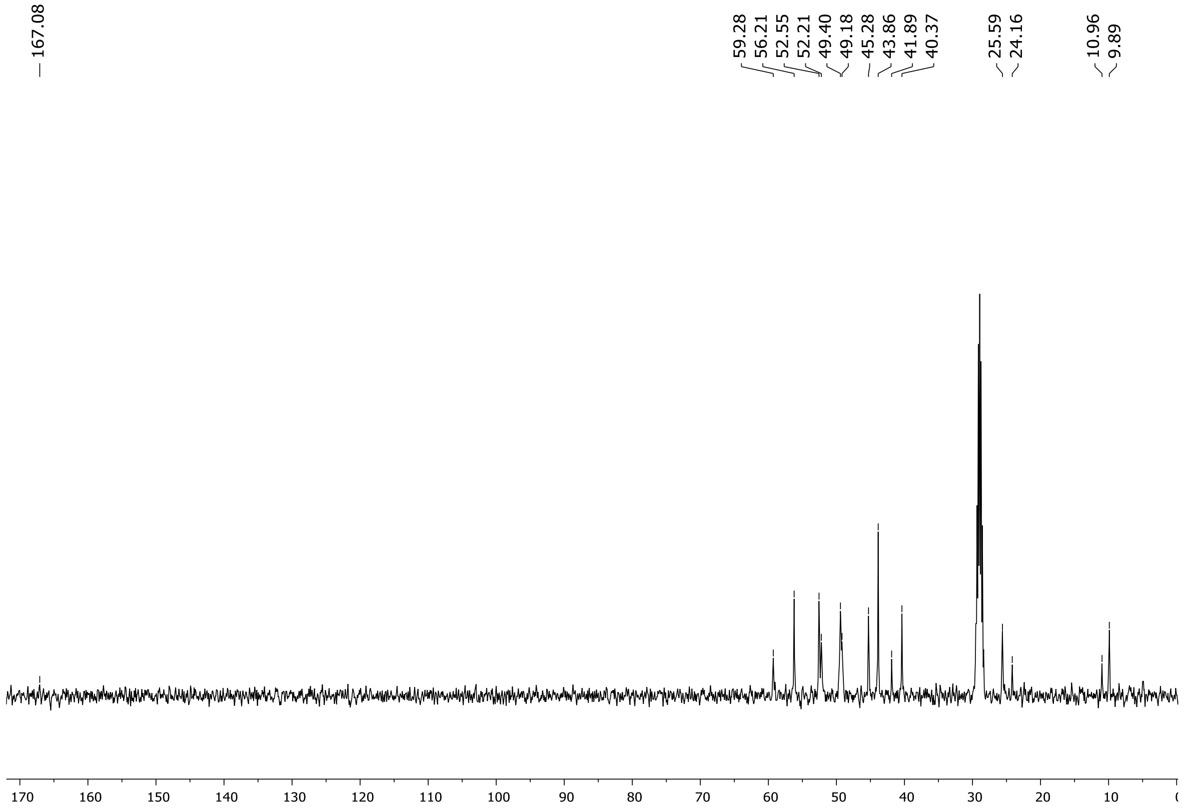

Figure S37. ^{13}C NMR spectrum of compounds **6a** and **6b** (acetone-d_6)

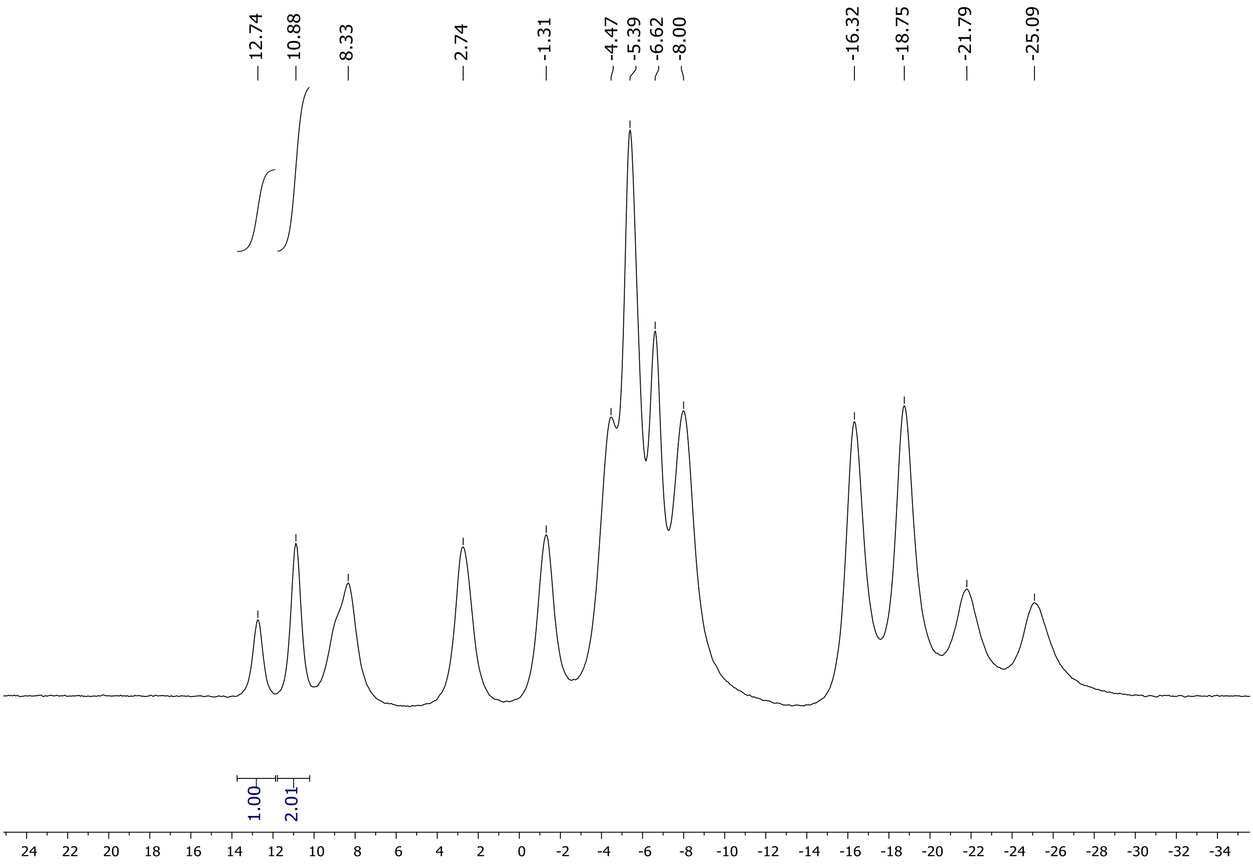

Figure S38. $^{11}B\{^{1}H\}$ NMR spectrum of compounds **6a** and **6b** (acetone-d_6)

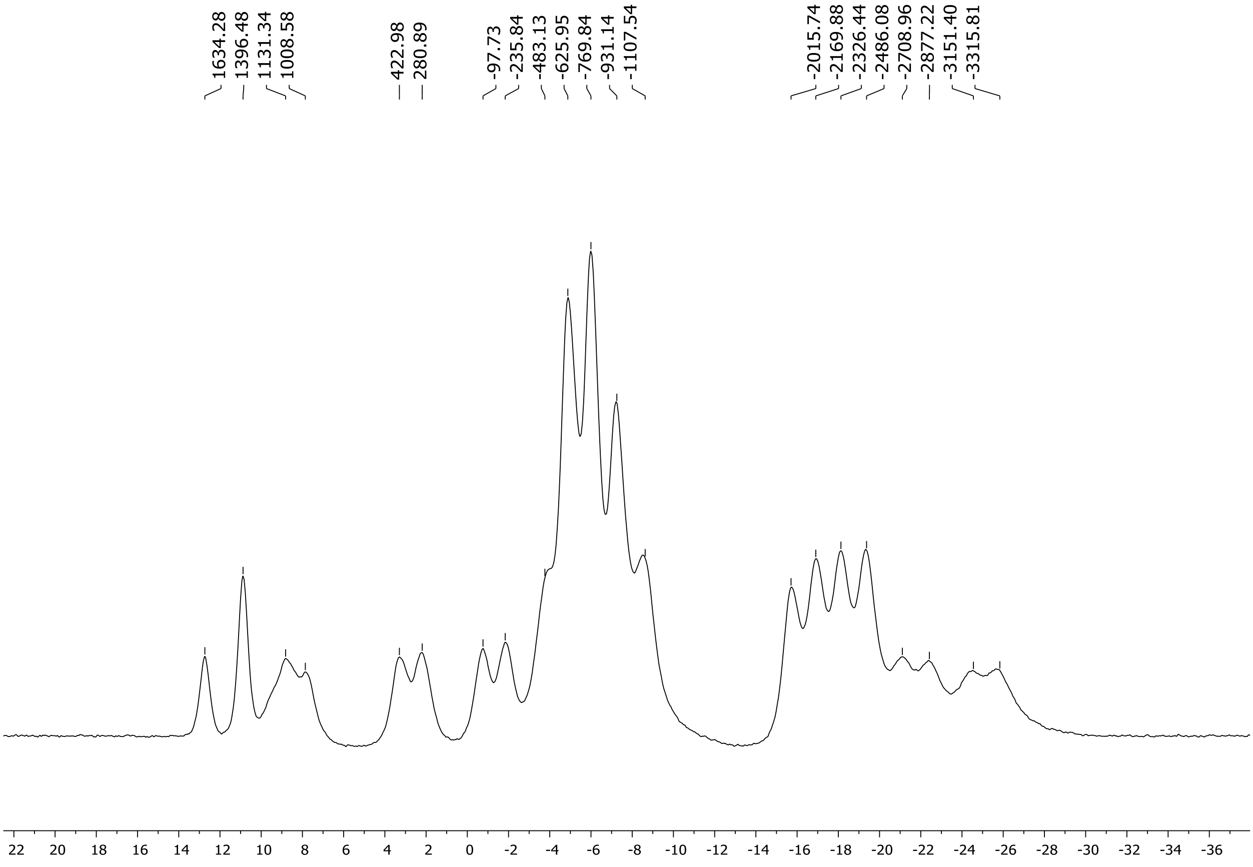

Figure S39. $^{11}B\{^{1}H\}$ NMR spectrum of compounds **6a** and **6b** (acetone-d_6)

Spectral data for [(8-EtC(NMe$_2$)=HN-3,3'-Co(1,2-C$_2$B$_9$H$_{10}$)(1',2'-C$_2$B$_9$H$_{11}$] (7)

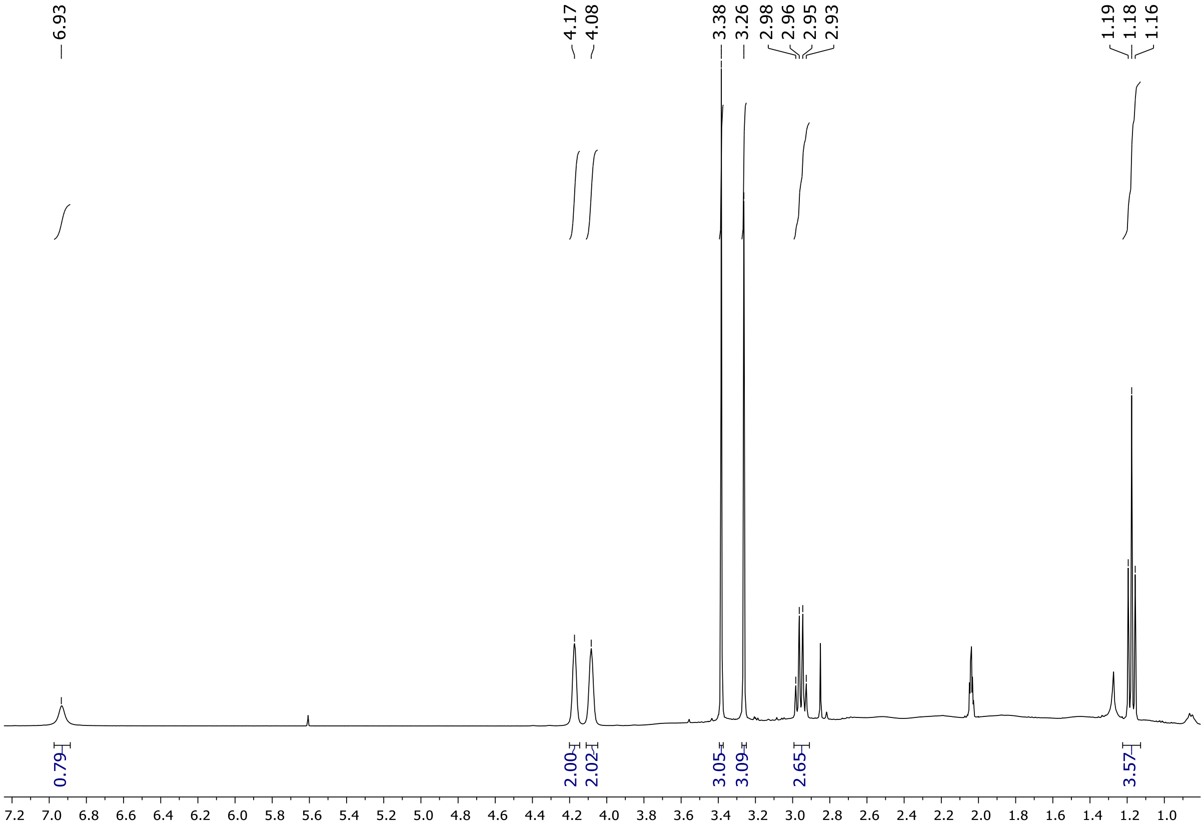

Figure S40. ^{1}H NMR spectrum of compound **7** (acetone-d$_6$)

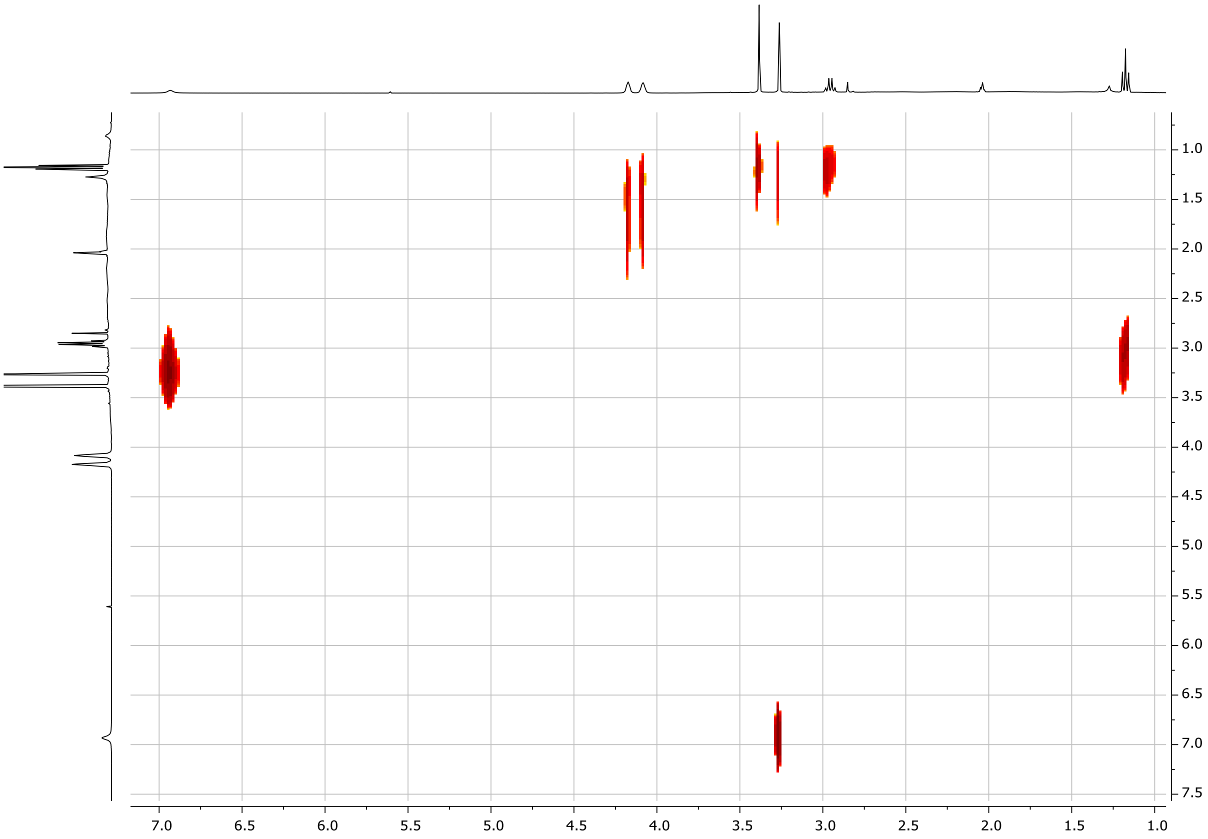

Figure S41. NOESY NMR spectrum of compound **7** (acetone-d$_6$) with diagonal peak suppression

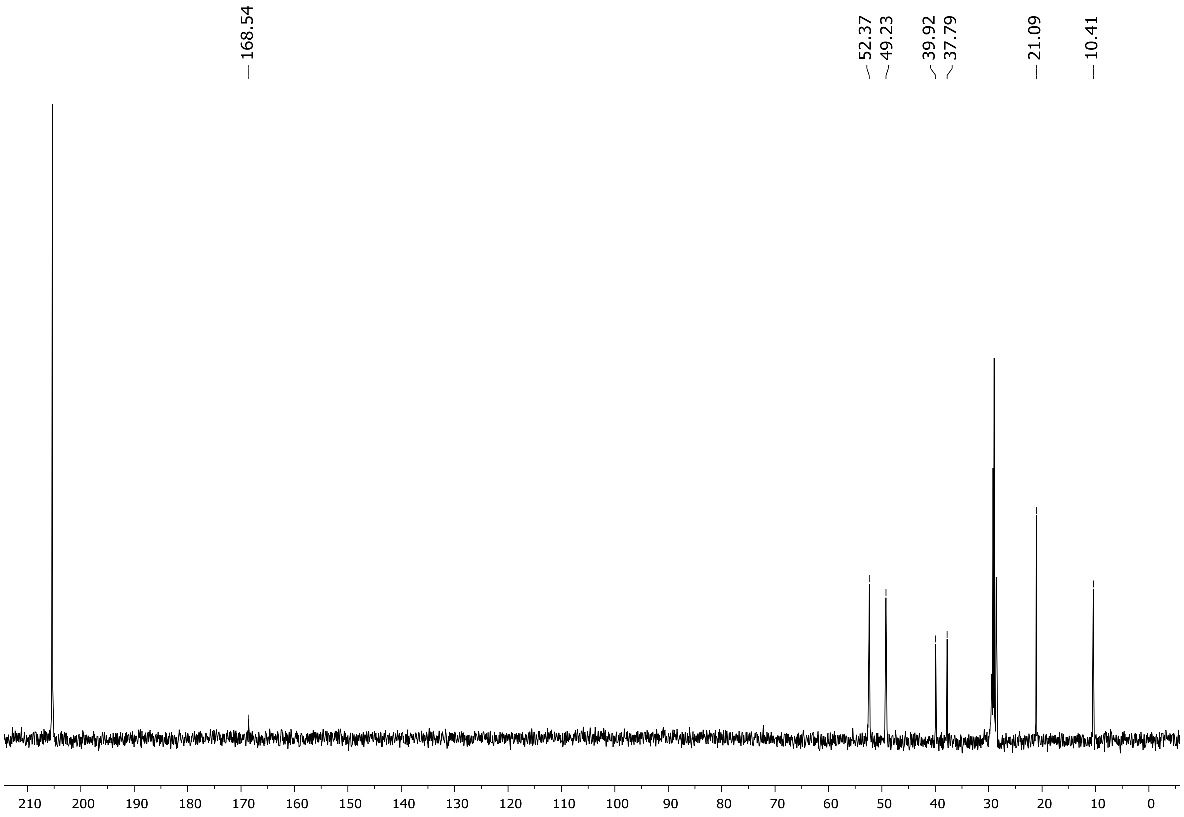

Figure S42. ^{13}C NMR spectrum of compound **7** (acetone-d_6)

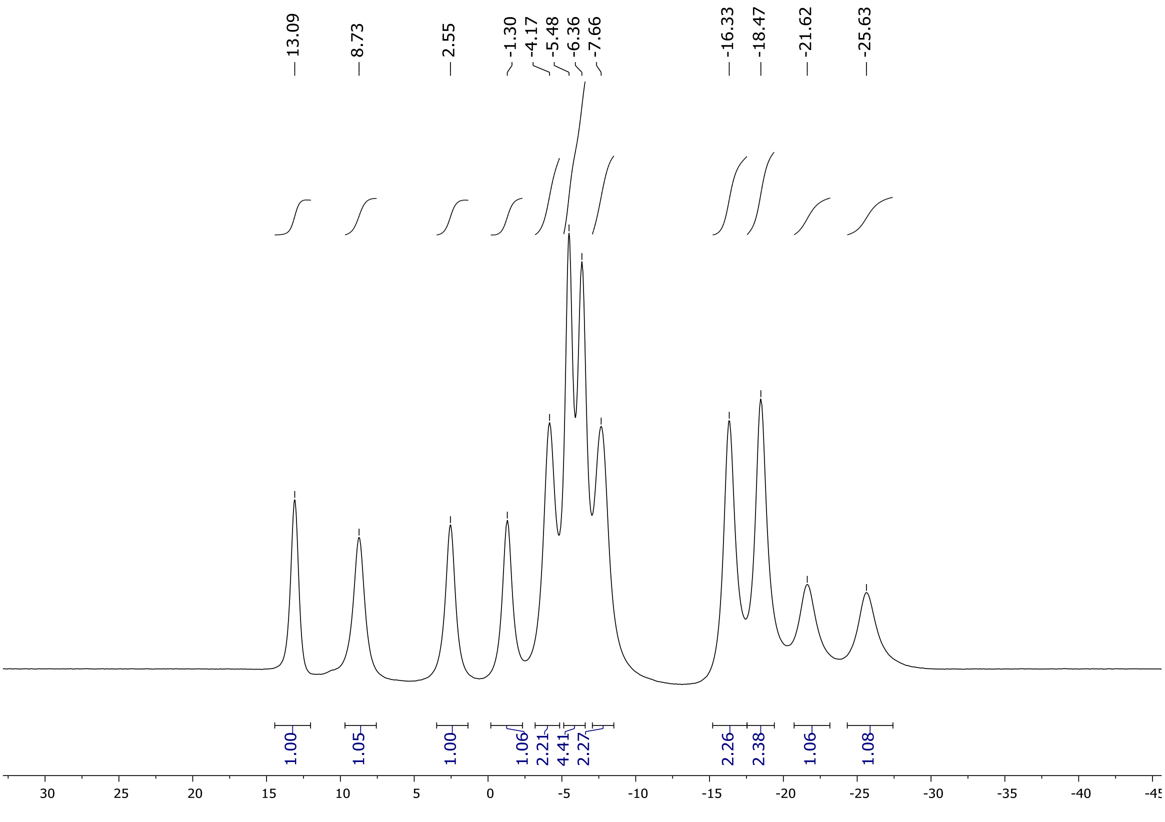

Figure S43. $^{11}B\{^1H\}$ NMR spectrum of compound **7** (acetone-d_6)

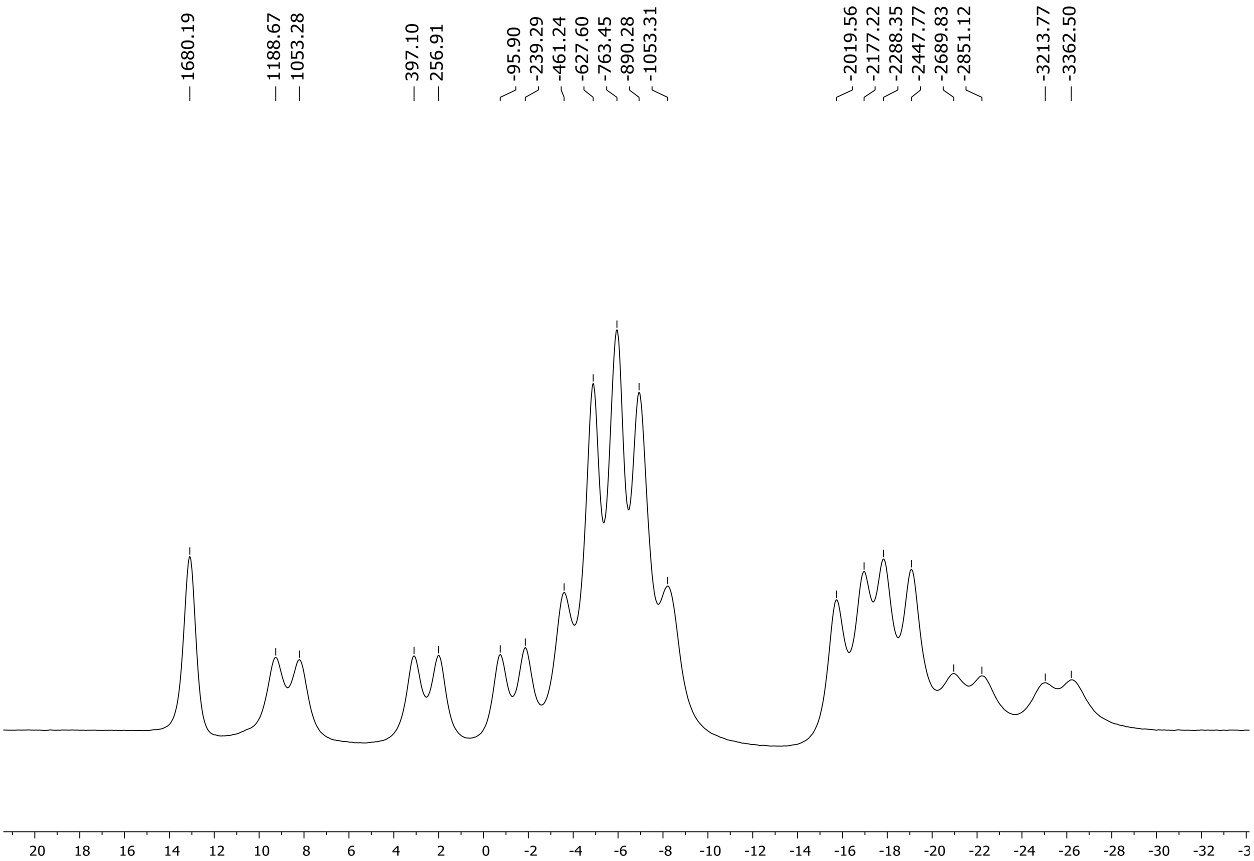

Figure S44. ^{11}B NMR spectrum of compound **7** (acetone-d_6)

Spectral data for [(8-EtC(NEt$_2$)=HN-3,3'-Co(1,2-$C_2B_9H_{10}$)(1',2'-$C_2B_9H_{11}$] (8)

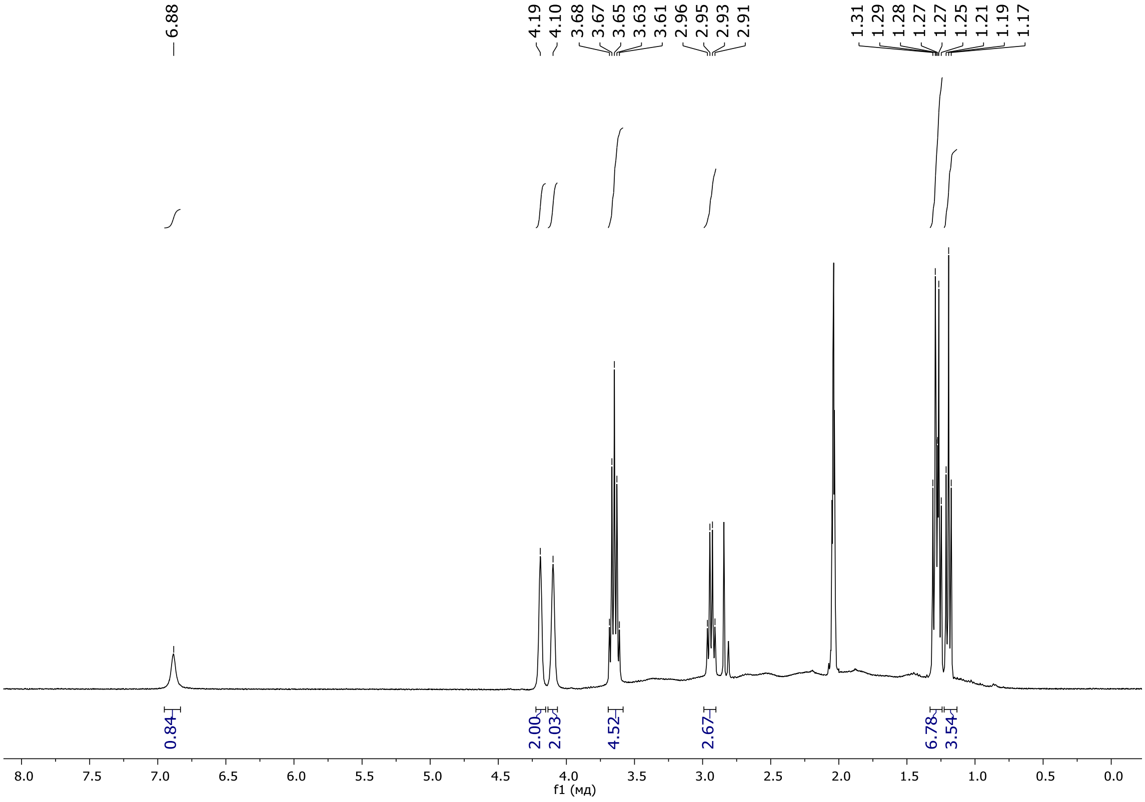

Figure S45. ^{1}H NMR spectrum of compound **8** (acetone-d$_6$)

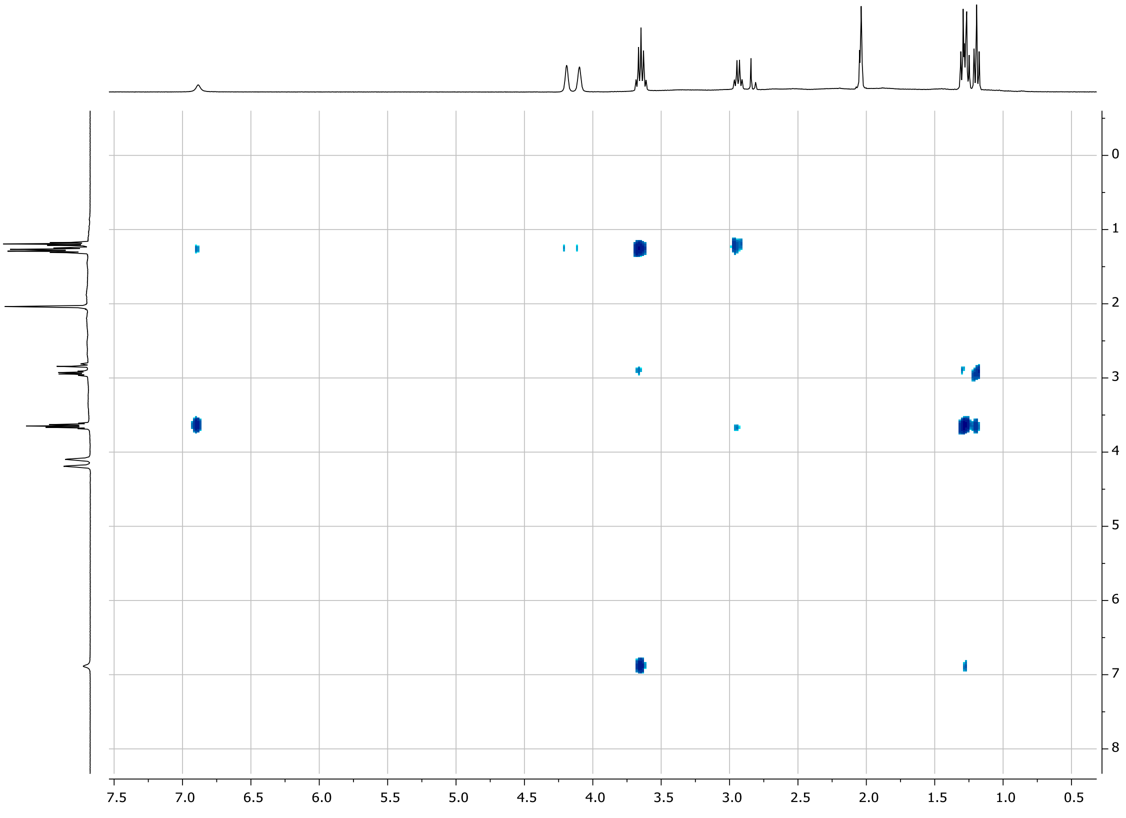

Figure S46. NOESY NMR spectrum of compound **8** (acetone-d$_6$) with diagonal peak suppression

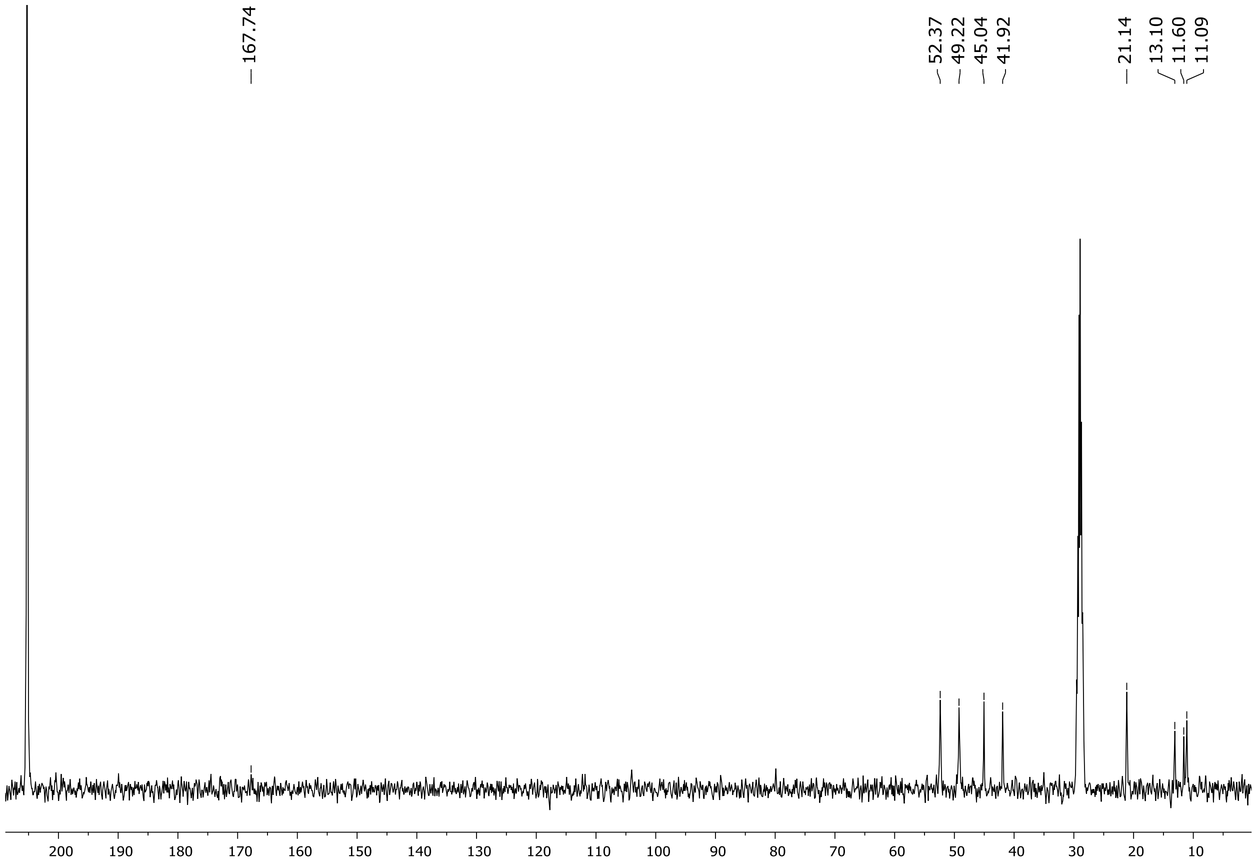

Figure S47. ^{13}C NMR spectrum of compound **8** (acetone-d_6)

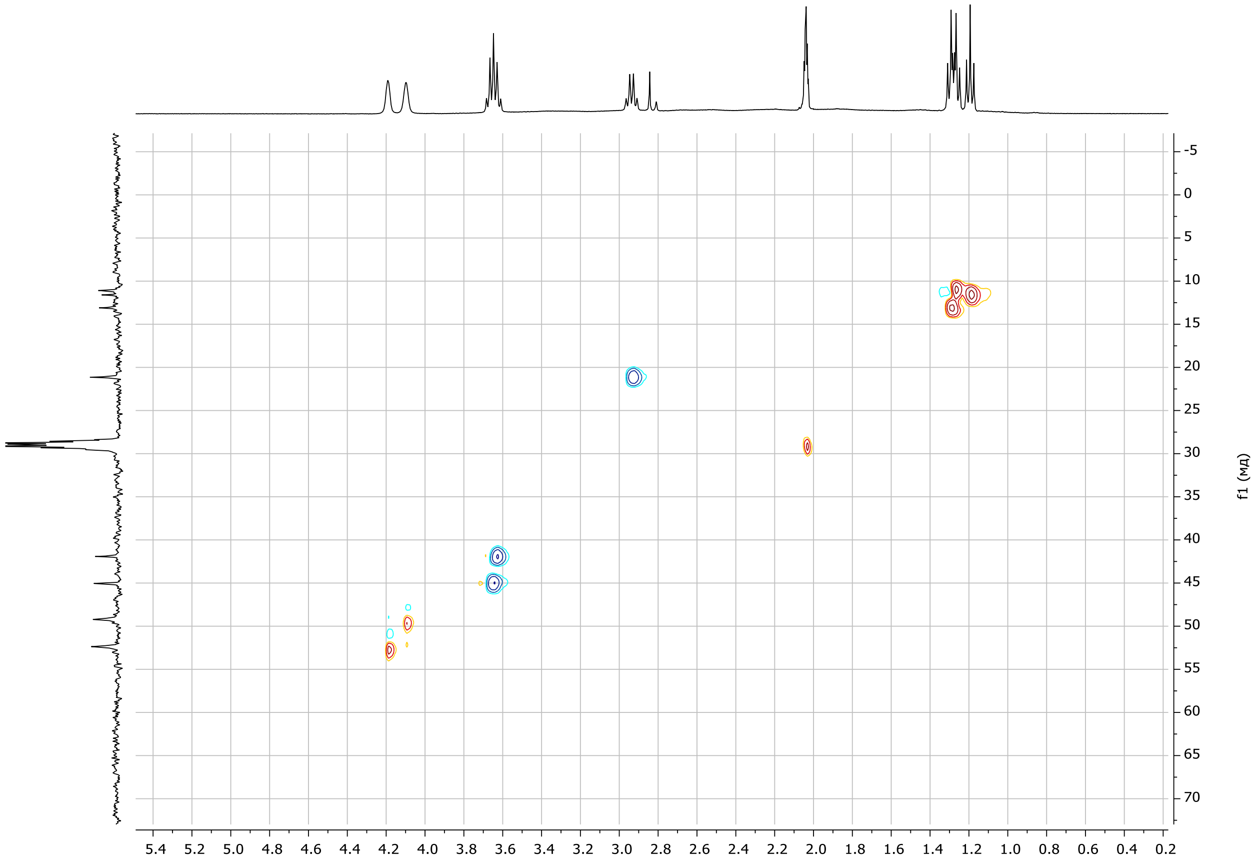

Figure S48. (HC)HSQC NMR spectrum of compound **8** (acetone-d_6)

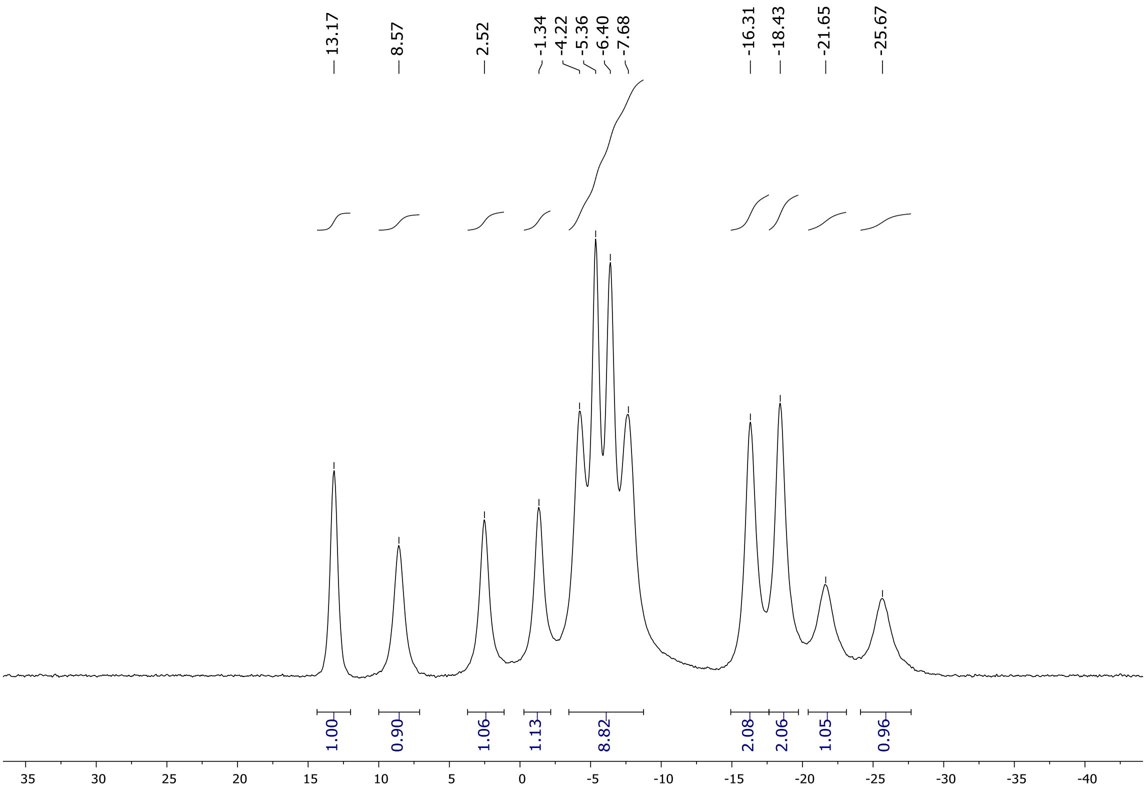

Figure S49. $^{11}B\{^{1}H\}$ NMR spectrum of compound **8** (acetone-d_6)

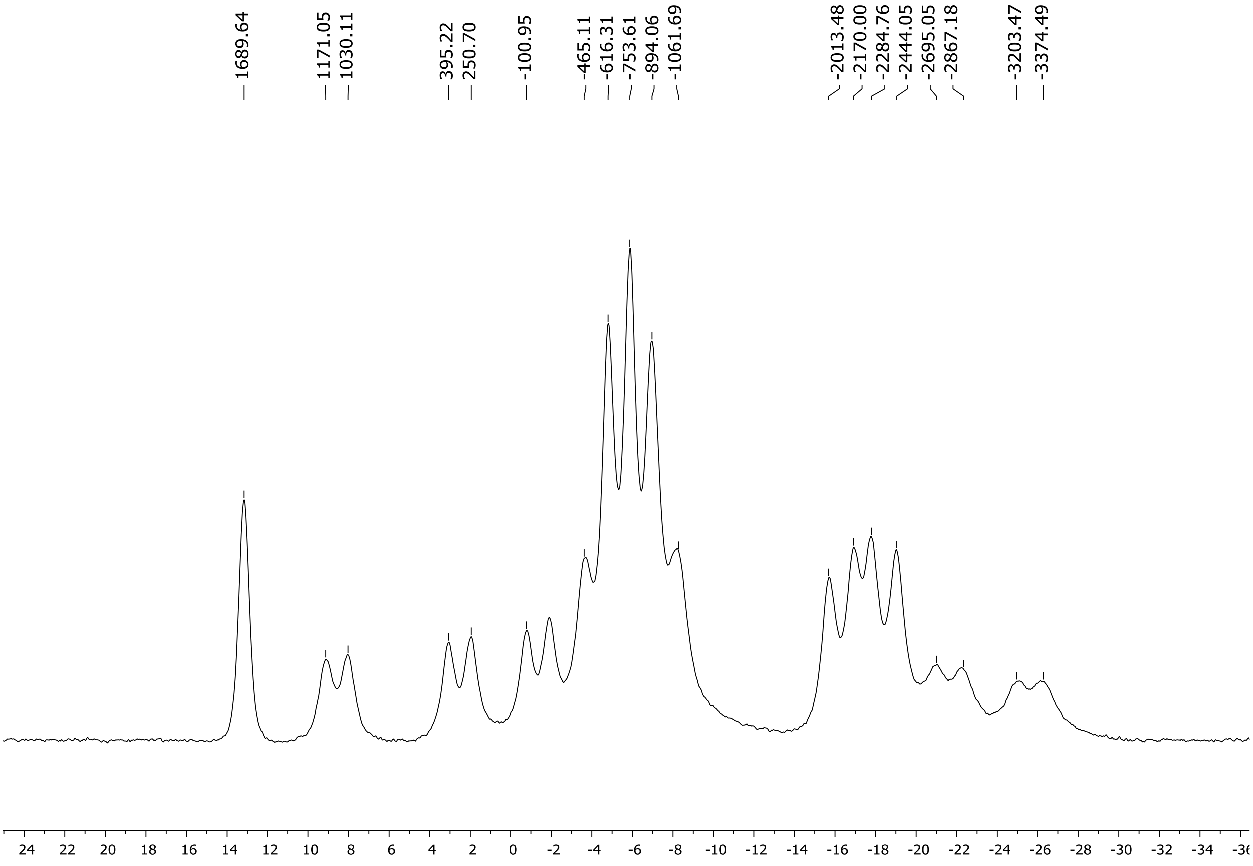

Figure S50. ^{11}B NMR spectrum of compound **8** (acetone-d_6)

Spectral data for [(8-EtC(NC_5H_{10})=HN-3,3'-Co(1,2-$C_2B_9H_{10}$)(1',2'-$C_2B_9H_{11}$)] (9)

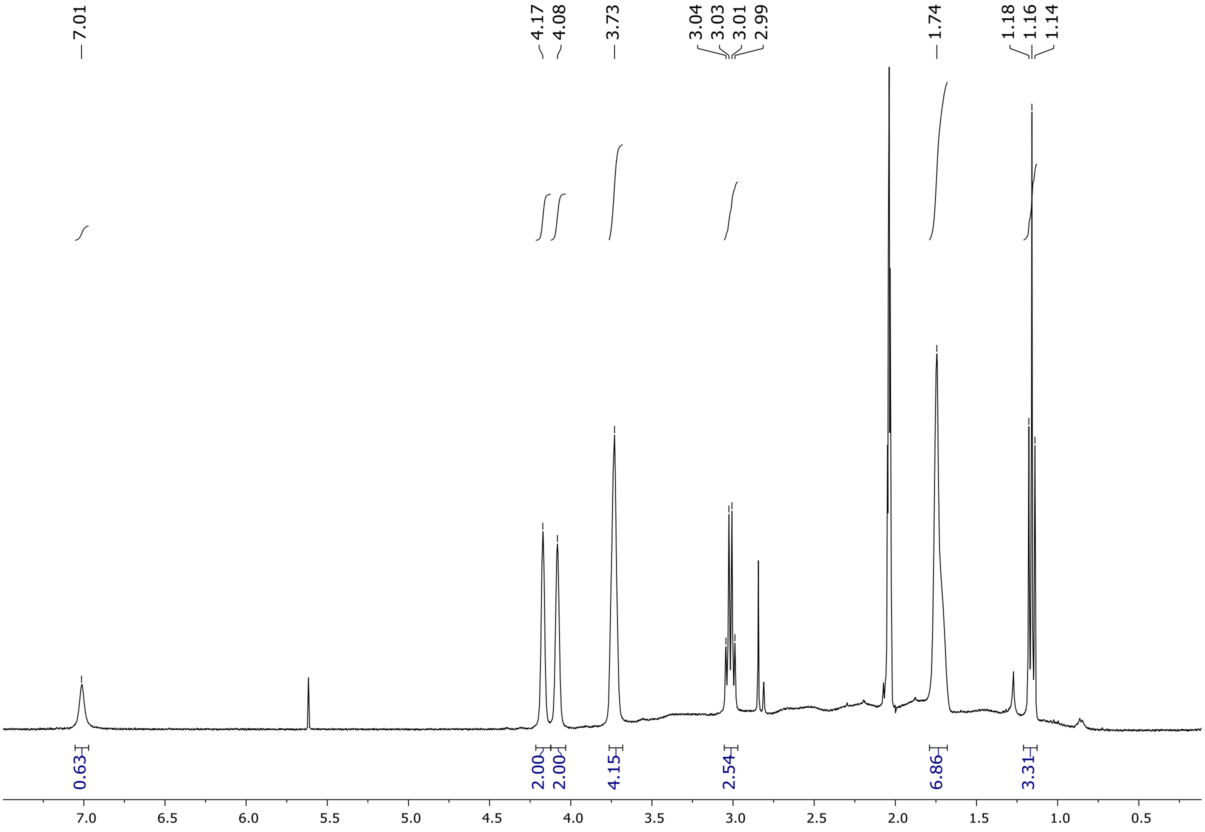

Figure S51. ^{1}H NMR spectrum of compound **9** (acetone-d_6)

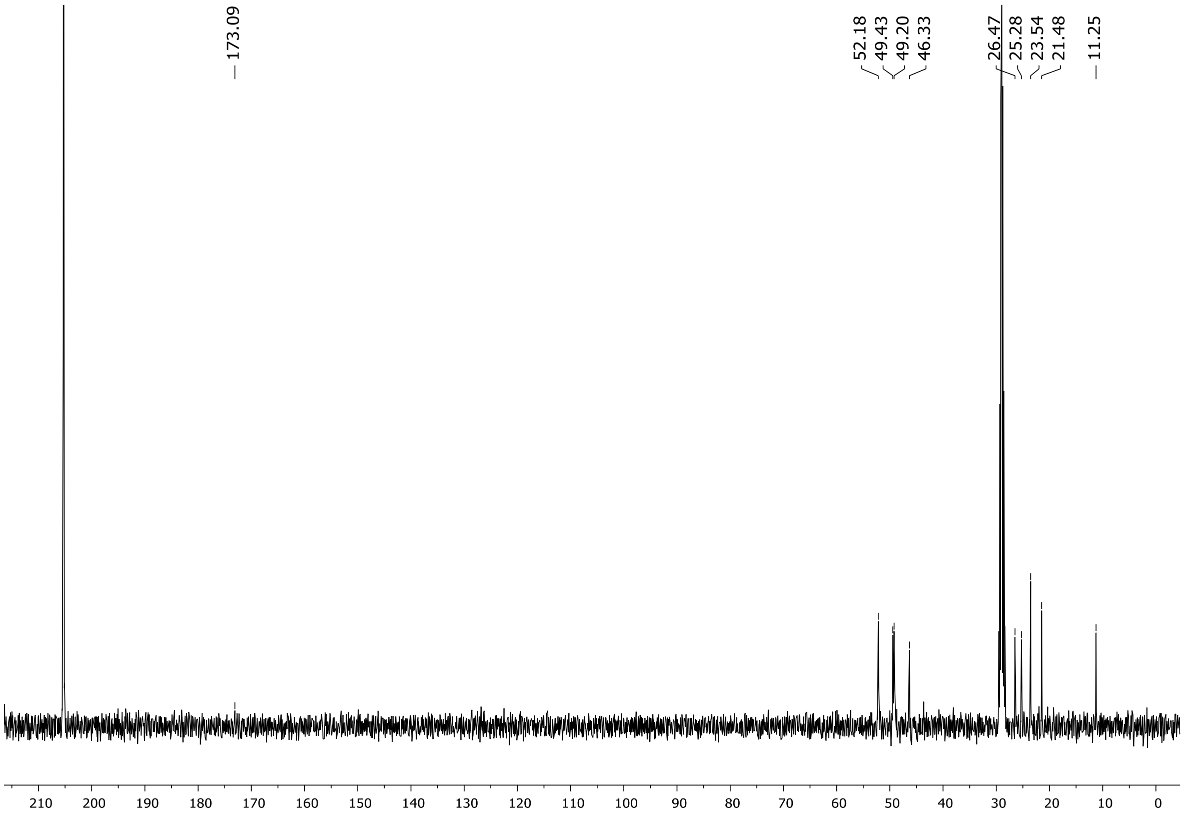

Figure S52. ^{13}C NMR spectrum of compound **9** (acetone-d_6)

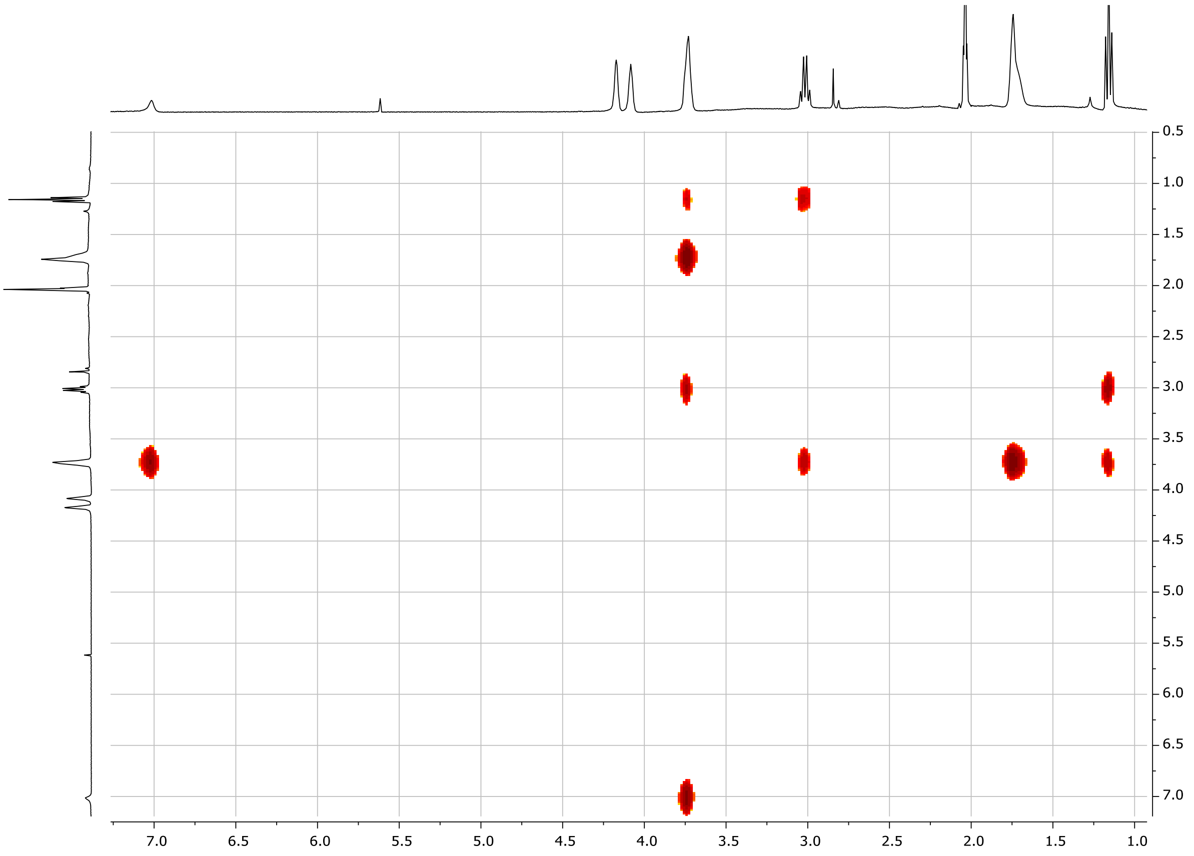

Figure S53. NOESY NMR spectrum of compound **9** (acetone-d_6) with diagonal peak suppression

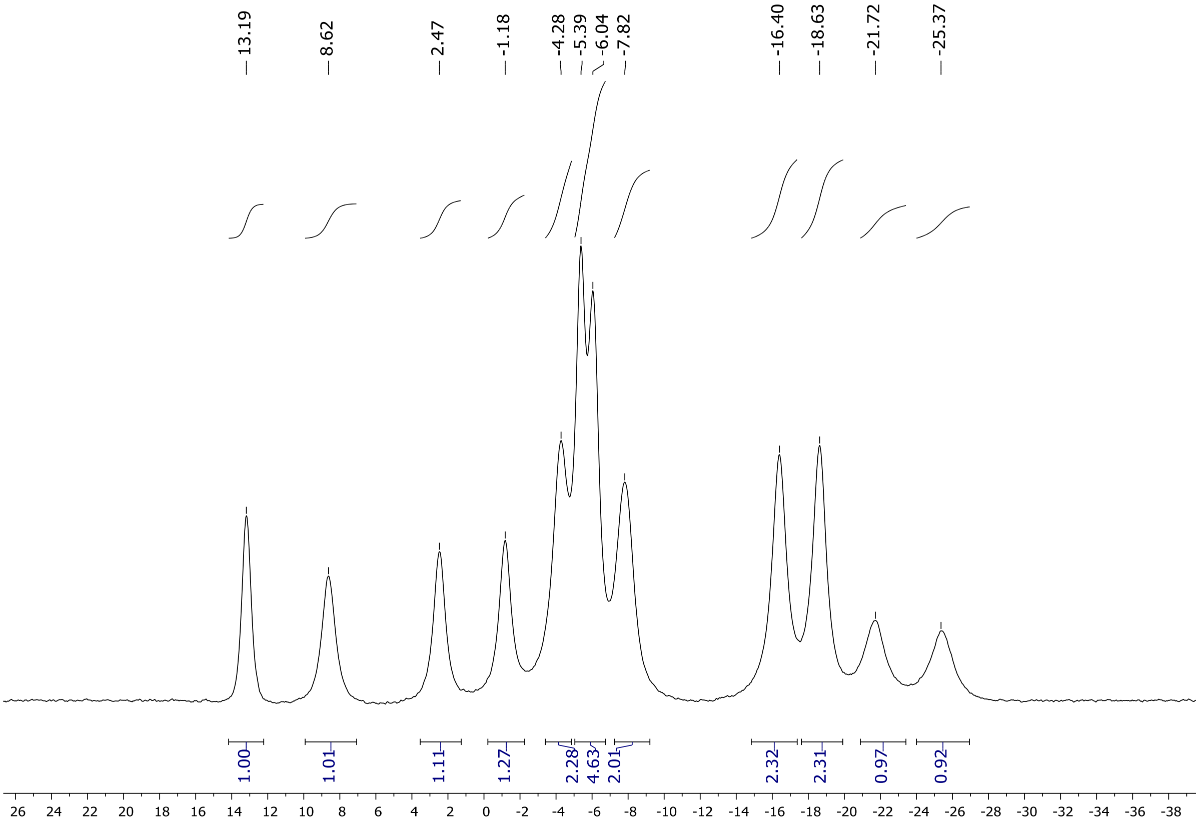

Figure S54. $^{11}B\{^1H\}$ NMR spectrum of compound **9** (acetone-d_6)

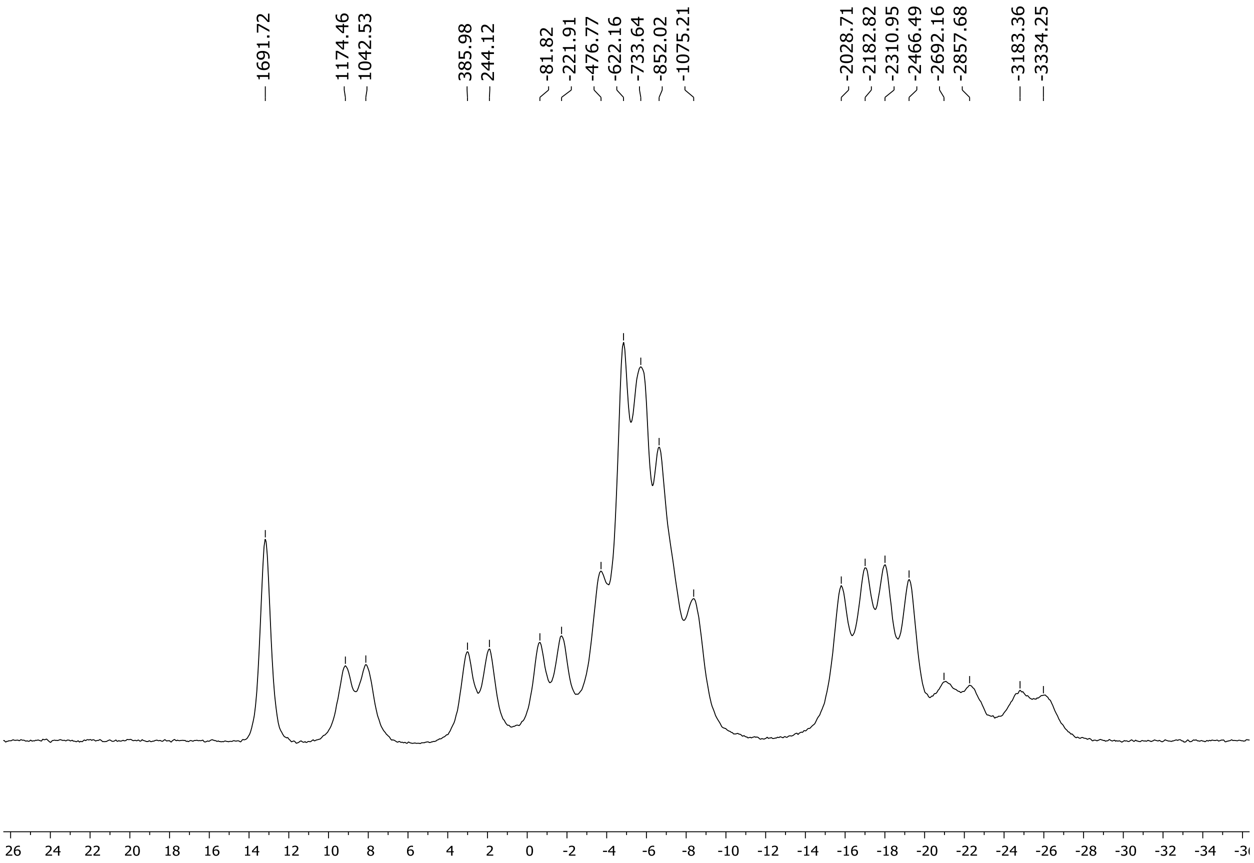

Figure S55. ^{11}B NMR spectrum of compound **9** (acetone-d$_6$)

Spectral data for [(8-EtC(NC_4H_8O)=HN-3,3'-Co(1,2-$C_2B_9H_{10}$)(1',2'-$C_2B_9H_{11}$] (10)

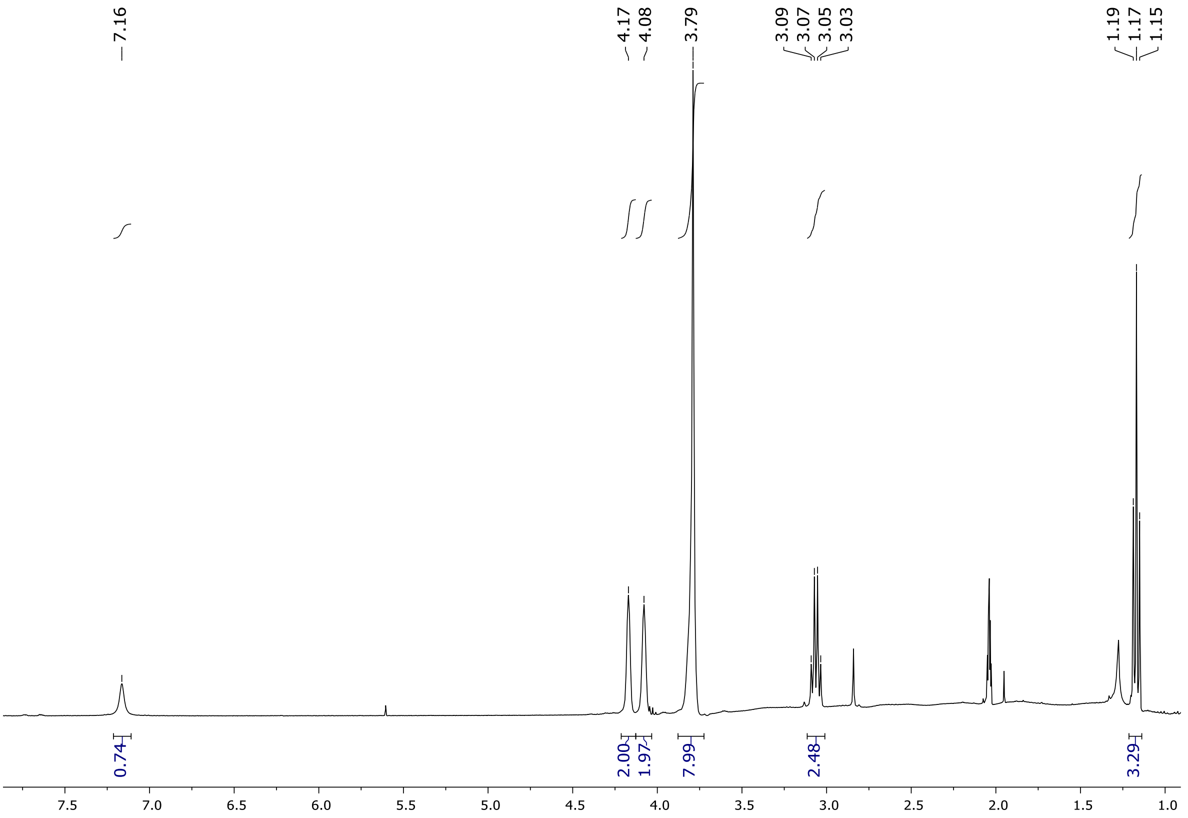

Figure S56. ^{1}H NMR spectrum of compound **10** (acetone-d_6)

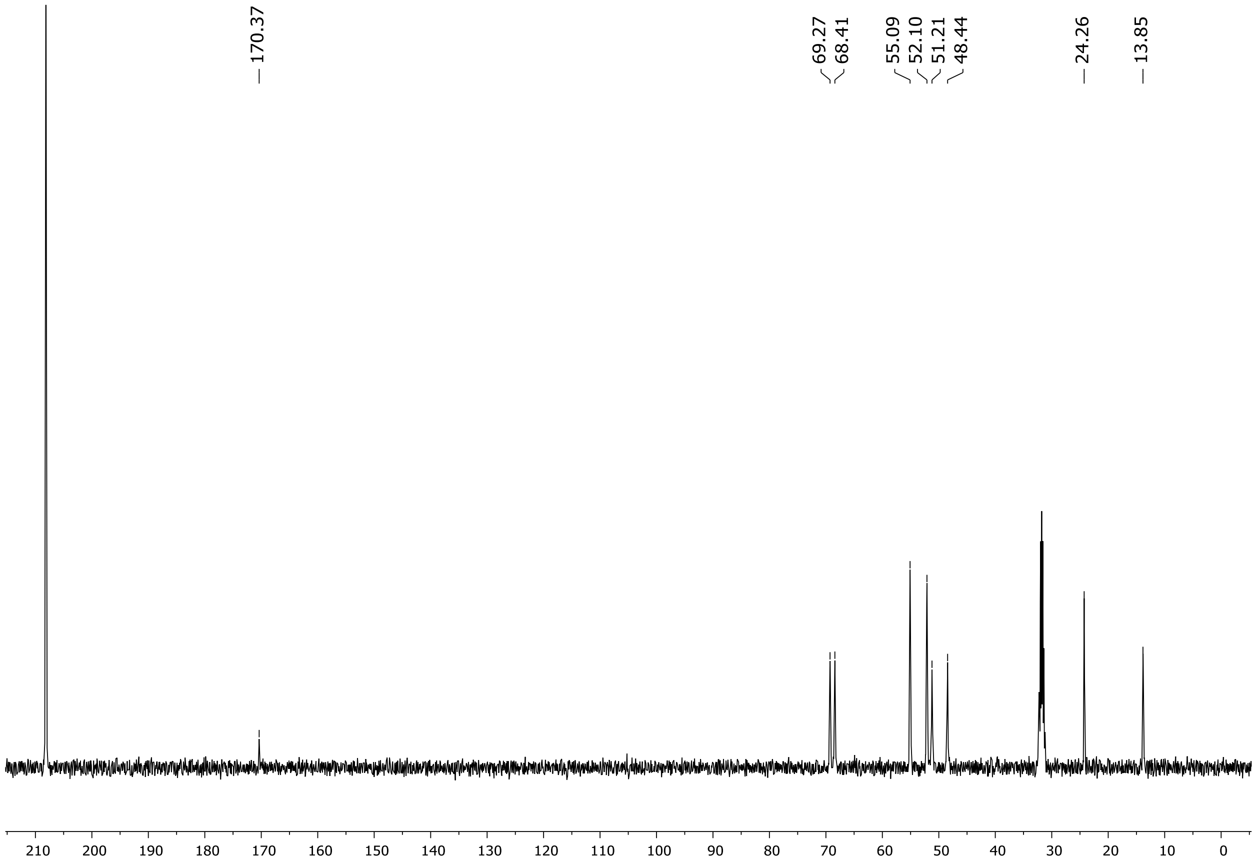

Figure S57. ^{13}C NMR spectrum of compound **10** (acetone-d_6)

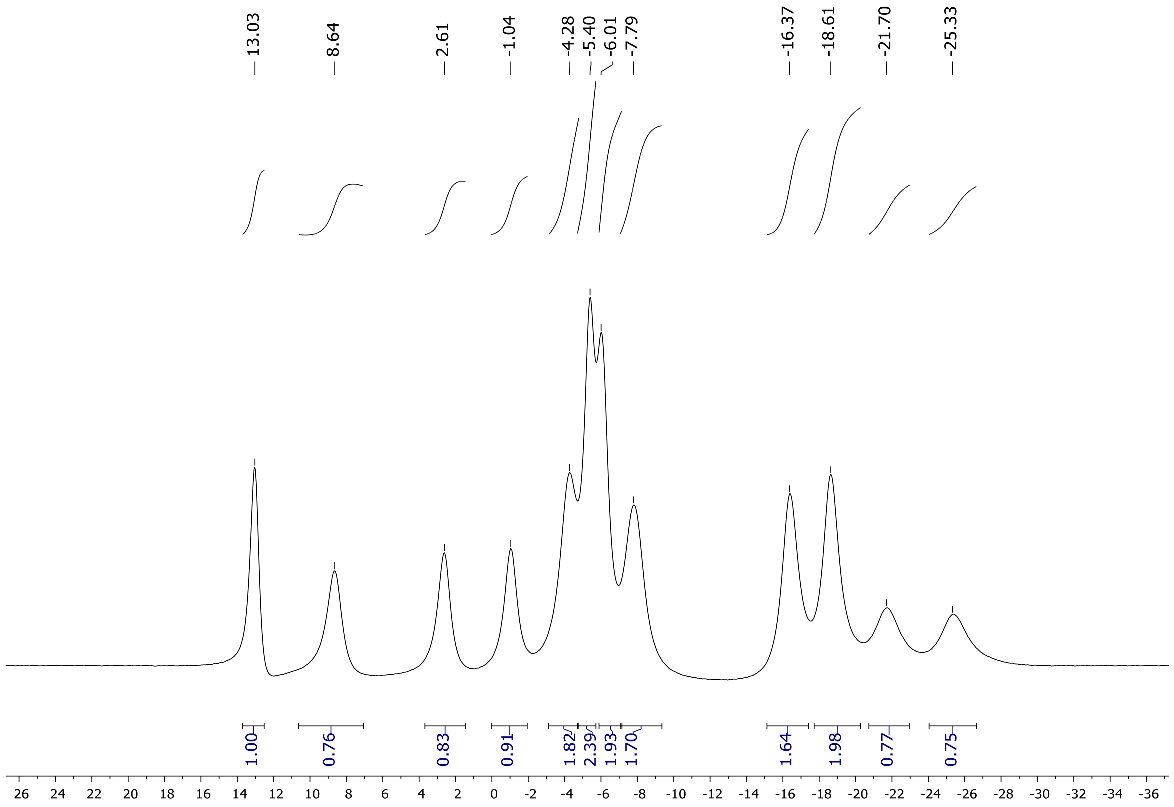

Figure S58. $^{11}B\{^1H\}$ NMR spectrum of compound **10** (acetone-d_6)

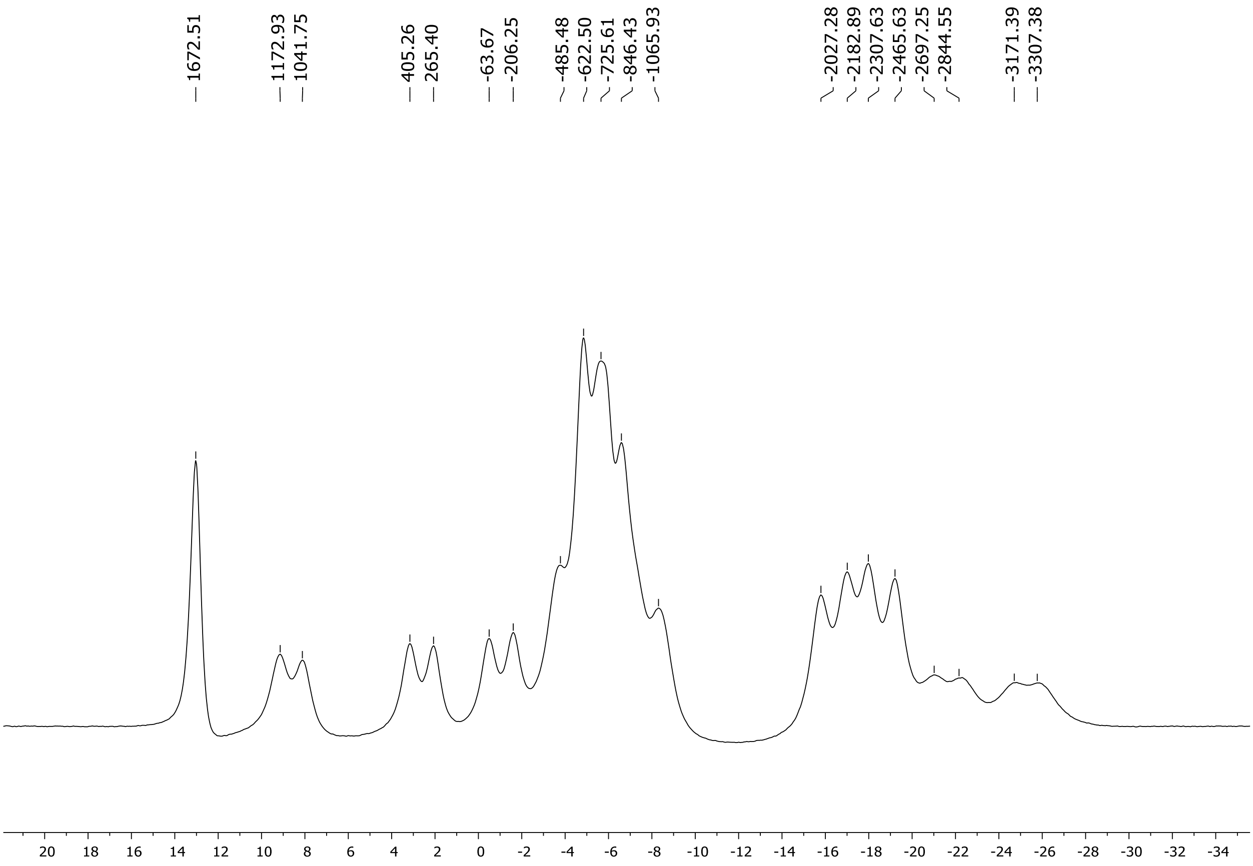

Figure S59. ^{11}B NMR spectrum of compound **10** (acetone-d_6)

Table S1. Crystallographic data for the SH197 and SH218.

	**7**	**8**	**9**
formula	$C_9H_{33}B_{18}CoN_2$	$C_{11}H_{37}B_{18}CoN_2$	$C_{12}H_{37}B_{18}CoN_2,\cdot 2H_2O$
fw	422.88	450.93	498.98
temperature, K	120	120	120
crystal system	Orthorhombic	Monoclinic	Tetragonal
space group	$Pna2_1$	$Pca2_1$	$I4_1/a$
a, Å	27.1787(4)	13.3717(4)	39.0901(11)
b, Å	7.08410(10)	16.2810(5)	39.0901(11)
c, Å	11.5151(2)	11.2565(3)	6.9515(3)
V, Å^3	2217.08(6)	2450.59(12)	10622.1(8)
Z	4	4	16
ρ_{calc}, g·cm^{-3}	1.267	1.222	1.248
F(000)	872	936	4160
μ, mm^{-1}	0.774	0.705	0.662
θ range, deg.	2.97 – 28.01	1.97 – 27.11	2.95 – 27.13
reflections collected	23976	21123	22402
independent reflections / R_{int}	5112/0.0350	5343/0.0508	5836/0.1199
Completeness to theta θ, %	99.8	99.9	99.3
refined parameters	362	380	299
GOF (F^2)	1.078	1.024	1.028
reflections with $I>2\sigma(I)$	4862	4406	3384
$R_1(F)$ ($I>2\sigma(I)$)[a]	0.0277	0.0326	0.0684
$wR_2(F^2)$ (all data)[b]	0.0619	0.0734	0.1626
Largest diff. peak/hole, e·Å^{-3}	0.286/-0.288	0.219/-0.241	0.646/-0.474

[a] $R_1 = \sum||F_o| - |F_c||/\sum(F_o)$; [b] $wR_2 = (\sum[w(F_o^2 - F_c^2)^2]/\sum[w(F_o^2)^2])^{½}$

Table S2. Selected bond lengths (Å) and torsion angles (Deg.) defining geometry and relative orientation of the substituent in compounds 7-9.

Bond or torsion angle	**7**	**8**	**9**
B8-N1	1.519(3)	1.522(4)	1.516(6)
N1-C3	1.312(3)	1.318(4)	1.313(5)
C3-N2	1.330(3)	1.329(4)	1.316(6)
C3-C4	1.493(4)	1.491(4)	1.508(6)
B7-B8-N1-C3	-23.8(5)	-27.4(6)	162.6(4)
B8-N1-C3-N2	171.4(3)	170.3(3)	-164.2(4)
N1-C3-N2-C6	-2.1(4)	-1.1(5)	6.2(8)

Table S3. Shortened H…H inramolecular contacts in compounds 7-9.

Contact	H…H Distance
N1-H1N…H8'-B8'	2.23(5)
N1-H1N…H8'-B8'	2.24(7)
N1-H1N…H4'-B4'	1.76
